# EEG-based dataset explicitly targets the transitions between sitting and standing for exploring neural activation patterns in motor imagery and execution

**DOI:** 10.1093/gigascience/giag065

**Published:** 2026-05-29

**Authors:** Benjakarn Uengsawapak, Supavit Kongwudhikunakorn, Suktipol Kiatthaveephong, Wipamas Polpakdee, Rattanaphon Chaisaen, Chanitsada Chuenchit, Poramate Manoonpong, Gun Bhakdisongkhram, Theerawit Wilaiprasitporn

**Affiliations:** School of Information Science and Technology (IST), Vidyasirimedhi Institute of Science and Technology (VISTEC), 555 Moo 1 Payupnai, Wangchan, Rayong 21210, Thailand; School of Information Science and Technology (IST), Vidyasirimedhi Institute of Science and Technology (VISTEC), 555 Moo 1 Payupnai, Wangchan, Rayong 21210, Thailand; School of Information Science and Technology (IST), Vidyasirimedhi Institute of Science and Technology (VISTEC), 555 Moo 1 Payupnai, Wangchan, Rayong 21210, Thailand; School of Information Science and Technology (IST), Vidyasirimedhi Institute of Science and Technology (VISTEC), 555 Moo 1 Payupnai, Wangchan, Rayong 21210, Thailand; School of Information Science and Technology (IST), Vidyasirimedhi Institute of Science and Technology (VISTEC), 555 Moo 1 Payupnai, Wangchan, Rayong 21210, Thailand; Sirindhorn International Institute of Technology, Thammasat University, 99 Moo 18, Paholyothin Road, Khlong Nueng, Khlong Luang, Pathum Thani 12120, Thailand; School of Information Science and Technology (IST), Vidyasirimedhi Institute of Science and Technology (VISTEC), 555 Moo 1 Payupnai, Wangchan, Rayong 21210, Thailand; School of Physical Medicine and Rehabilitation, Institute of Medicine, Suranaree University of Technology, 111 University Avenue, Suranaree, Muang, Nakhon Ratchasima 30000, Thailand; School of Information Science and Technology (IST), Vidyasirimedhi Institute of Science and Technology (VISTEC), 555 Moo 1 Payupnai, Wangchan, Rayong 21210, Thailand

**Keywords:** EEG dataset, lower-limb motor imagery, lower-limb motor execution, sit-to-stand transition, stand-to-sit transition, brain–computer interface (BCI), event-related desynchronization (ERD), movement-related cortical potential (MRCP)

## Abstract

This study presents the first publicly accessible electroencephalography (EEG) dataset explicitly targeting sit-to-stand and stand-to-sit transitions during both motor execution (ME) and motor imagery (MI) tasks. Twenty-two healthy participants performed sitting and standing transitions under well-controlled experimental conditions while 60-channel EEG, electrooculography (EOG), and electromyography (EMG) signals were synchronously recorded. The dataset enables the exploration of neural activation patterns associated with lower-limb movements and supports the development of EEG-based brain–computer interface (BCI) algorithms for mobility assistance and rehabilitation. To validate the dataset, benchmark classification was conducted on three baseline deep learning methods—CTNet, EEGNet, and TCANet. Given the high inter-subject variability inherent to EEG, leave-one-subject-out cross-validation is used to ensure no subject bias during evaluation. Results demonstrated consistent decoding performance with mean accuracies of approximately 81% for ME and 73% for MI, indicating the reliability and usability of the dataset. Additionally, analyses of movement-related cortical potentials (MRCPs) and event-related desynchronization/synchronization (ERD/ERS) patterns revealed distinct neural signatures across the transition phases. This dataset provides a comprehensive foundation for studying lower-limb motor control, neural dynamics, and the advancement of MI-based BCIs for rehabilitation and assistive technologies.

## Data description

### Background and purpose

Motor imagery (MI), the mental simulation of movement without physical execution, is a central yet challenging paradigm in electroencephalography (EEG)-based brain–computer interfaces (BCIs). MI-BCIs harness intentional brain activity to control external devices, such as assistive tools and computers, purely through thought. This paradigm shows strong potential in rehabilitation, neuroprosthetics, and assistive technologies, particularly for individuals with motor impairments, including stroke survivors and patients with neurodegenerative diseases [1]. Typically, MI-BCIs involve imagery of specific movements, such as hand or foot actions, that activate sensorimotor brain regions. These activations produce distinctive EEG patterns in the 8–30 Hz frequency range, characterized by event-related desynchronization (ERD)—a reduction in amplitude before or during the event—and event-related synchronization (ERS)—an increase in amplitude afterward. These rhythms form the foundation for decoding motor intentions for neurorehabilitation applications.

Beyond hand and foot movements, investigating the neural signals associated with sit-to-stand (sit–stand) and stand-to-sit (stand–sit) transitions provides valuable insights for developing mobility rehabilitation protocols and assistive devices. Recent studies have demonstrated the feasibility of utilizing EEG signals during sit–stand and stand–sit imagery to design MI-BCI systems that support lower-limb movement by accurately decoding motor intentions [2–6]. Building on these findings, MI-BCIs for these tasks hold promise for real-world applications, allowing smoother transitions between sitting and standing positions.

Despite its potential, sit-stand and stand-sit transitions remain underrepresented in MI-BCI research. Current publicly available MI-BCI EEG datasets predominantly focus on traditional hand and foot movements due to their well-defined and distinctive neural patterns [7, 8]. While these datasets have supported the development of advanced EEG-based classification algorithms, none offers EEG recordings of transitional sit-stand or stand-sit motor imagery. The lower-limb movement datasets have been well presented in the study by Triana-Guzman et al. [5]; however, their main objective lies on classifying completed movement task. In practical BCI scenarios, distinguishing transition-related neural activity from initial resting states and detecting early movement intention are critical challenges. This gap underscores the need for dedicated datasets focusing on these transitions to better capture lower-limb dynamics and expand the application of MI-BCIs in rehabilitation.

To advance research in this area, we present an EEG dataset comprising recordings from 22 participants during motor imagery and motor execution (ME) of sit-stand and stand-sit transitions, featuring up to 60 EEG channels. In addition to EEG, electrooculography (EOG) was collected to support artifact removal related to eye movements during standard preprocessing, and electromyography (EMG) was recorded concurrently to precisely determine movement onset during ME trials. To the best of our knowledge, this is the first publicly accessible EEG dataset explicitly designed to target sit-stand and stand-sit transitions. It provides a valuable resource for researchers investigating lower-limb motor intentions reflected in EEG activity and developing EEG-based algorithms dedicated to lower-limb MI-BCIs. This dataset directly contributes a significant step in publicly available MI-BCI resources to advance lower-limb research and applications.

## Experimental design

### Participants

Twenty-three healthy participants (aged 22–28 years; fifteen males) with no known neurophysiological abnormalities were recruited for this study. One participant (S05) was excluded due to poor signal quality, resulting in a final cohort of twenty-two participants. Prior to the experiment, research staff provided both verbal and written explanations of the study objectives, protocol, questionnaire, and experimental setup to ensure participants’ understanding. A written consent form was obtained from all participants in accordance with the Declaration of Helsinki. Participants received monetary compensation for their involvement. The experimental protocol and environment were reviewed and approved by the Ethics Committee of Suranaree University of Technology, Thailand (EC-65-0031). The demographic of the subject and the questionnaire used in this study are provided in the supplementary material.

### Environment

**Figure 1:**
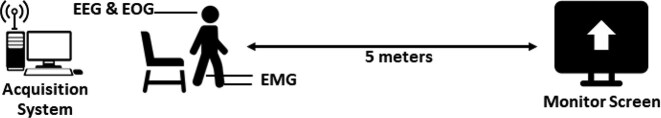
Experimental setup for data collection. A monitor displaying visual cues was placed in front of the participant, while the data acquisition system for synchronous EEG, EOG, and EMG recording operated by research staff was behind the participant.

All experiments were conducted in a quiet, controlled environment with only the participant and research staff present. The experimental setup is illustrated in Fig. 1. For the sitting state, participants were seated approximately 5 meters from a 65-inch wall-mounted 4K LED monitor, oriented toward the monitor displaying visual cues. For the standing state, participants positioned themselves directly in front of the chair. The data acquisition system comprising a desktop computer running Windows 11, equipped with a 24-inch monitor, keyboard, and mouse, was placed on a table behind the participant. Research staff operated the system to synchronously record EEG, EOG, and EMG signals while ensuring the correct sequence of visual cues. To minimize fatigue effects, participants were instructed to rest adequately the night before, and all data collection sessions were scheduled during morning hours to ensure an optimal physiological state.

## Data acquisition

### Data collection protocol

**Figure 2:**
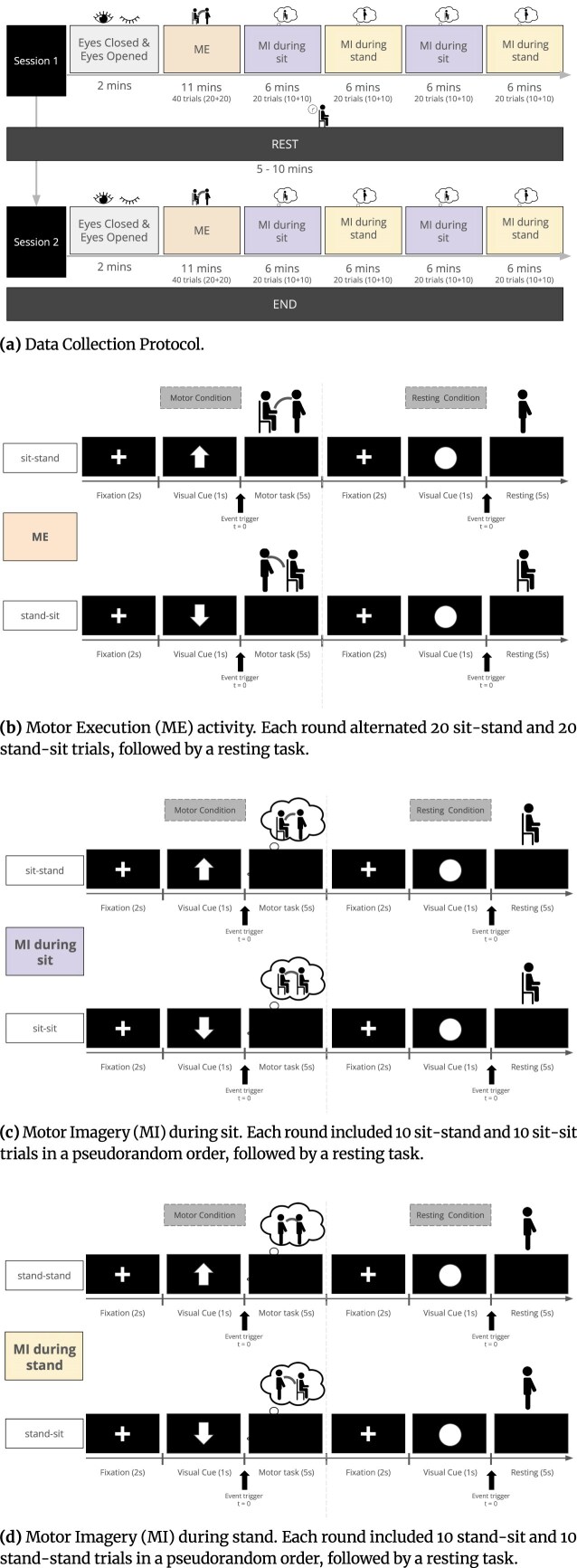
Overview of the data collection process and task-related instructions. Figure 2a shows overall protocol; highlighted regions are detailed in (b–d). Figure 2b shows visual cue instructions for motor execution (ME). Figure 2c shows visual cue instructions for motor imagery (MI) during sit. Figure 2d shows visual cue instructions for MI during stand.

Data acquisition was conducted in two identical sessions of motor tasks, separated by a 5–10 minute rest period. At the beginning of each session, baseline brain activity was recorded under two resting-state conditions: one minute with eyes closed (EC) followed by one minute with eyes open (EO). Participants then performed the ME task (40 trials per session), after which they completed the MI tasks. The MI tasks alternated between sitting and standing conditions and were repeated twice per session (20 trials per session). The overall procedure—including participant preparation, system setup, and data collection across both sessions—lasted approximately two hours per participant. The flow of the data collection protocol is visualized in Fig. 2a.

#### Motor execution

After collecting baseline EEG at the beginning of each session, participants performed the ME task, in which they carried out physical sit-stand (ME_SIT_STD) and stand-sit (ME_STD_SIT) movements. The task consisted of 40 trials, alternating between sit-stand and stand-sit transitions. For sit-stand trials, participants began seated on a chair, whereas for stand-sit trials, participants began standing in front of a chair. Each trial lasted 16 seconds, resulting in a total duration of 640 seconds (approximately 11 minutes), as shown in Fig. 2b.

The structure of each trial was as follows: a fixation cross appeared for 2 seconds to direct participants’ gaze and signal trial preparation. A visual cue was then presented for 1 second: an upward arrow instructed participants to stand up from a seated position, while a downward arrow instructed them to sit down from a standing position. After the cue disappeared, participants executed the instructed movement within 5 seconds. Another fixation cross then appeared for 2 seconds, followed by a 1-second presentation of a white circle, instructing participants to rest (ME_R) in their current posture for the subsequent 5 seconds. This marked the completion of one trial.

#### Motor imagery

Following the ME task, participants performed the MI task under two conditions: during sitting and standing. In contrast to the previous task, participants were instructed to mentally simulate the sit-stand and stand-sit transitions without executing any physical movement.

#### Motor imagery during sit

In the MI during sit condition (Fig. 2c), participants sat on a chair and observed sequential visual cues. Each 16-second trial began with a white cross for 2 seconds, prompting participants to focus and prepare for the upcoming cue. Subsequently, a 1-second visual cue appeared—a white upward arrow indicating participants should imagine standing up from sitting (MI_SIT_STD) or a white downward arrow indicating participants should imagine sitting down while already seated (MI_SIT_SIT). Participants imagined these movements for 5 seconds immediately after the cue disappeared. Another white cross appeared for 2 seconds, signaling preparation for the next cue, followed by a 1-second white circle instructing participants to rest. Participants then rested for 5 seconds (MI_R_SIT) while remaining seated to prevent fatigue.

MI during sit was divided into two nonconsecutive rounds, each consisting of 20 pseudorandomized trials (10 for MI_SIT_STD and 10 for MI_SIT_SIT). In the remaining part of this study, the MI_SIT_SIT task data is not analyzed, as they do not involve directional motor transitions and are expected to produce neural signatures closely resembling resting states. However, the data are provided to enable further exploration in potential studies, such as the detection of a user’s static postural intentions.

#### Motor imagery during stand

In the MI during stand condition (Fig. 2d), participants stood in front of a chair while observing sequential visual cues. Each 16-second trial began with a white cross displayed for 2 seconds, signaling participants to prepare for the upcoming instruction. Next, a 1-second visual cue appeared—a white downward arrow instructing participants to imagine sitting down from standing (MI_STD_SIT) or a white upward arrow instructing participants to imagine standing up while already standing (MI_STD_STD). Participants imagined these movements for 5 seconds immediately after the cue disappeared. Another white cross appeared for 2 seconds, signaling preparation for the next cue, followed by a 1-second white circle instructing participants to rest. Participants then rested for 5 seconds (MI_R_STD) while remaining standing to avoid fatigue.

MI during stand was divided into two nonconsecutive rounds, each consisting of 20 pseudorandomized trials (10 for MI_STD_SIT and 10 for MI_STD_STD). In the remaining part of this study, the MI_STD_STD task data is not analyzed, as they do not involve directional motor transitions and are expected to produce neural signatures closely resembling resting states. However, the data are provided to enable further exploration in potential studies, such as the detection of a user’s static postural intentions.

### EEG signals

Electroencephalography recordings were acquired using a biosignal amplifier (g.HIamp, g.Tec, Austria) with a sampling rate of 1,200 Hz from 62 electrodes (60 EEG, 2 EOG) arranged according to the international 10–20 system. Of these, EEG signals were recorded using 60 electrodes, while horizontal (hEOG) and vertical (vEOG) EOG signals were acquired from two additional electrodes, as described in the subsequent section. The 60 EEG channels recorded included Fp1, Fp2, AF7, AF8, F7, F8, FT7, FT8, AF3, AF4, AFz, Fz, F1, F2, F3, F4, F5, F6, FCz, Cz, FC1, FC2, FC3, FC4, FC5, FC6, C1, C2, C3, C4, C5, C6, CPz, Pz, CP1, CP2, CP3, CP4, CP5, CP6, TP7, TP8, P1, P2, P3, P4, P5, P6, P7, P8, POz, Oz, PO3, PO4, PO7, PO8, PO9, PO10, O1, and O2.

**Figure 3:**
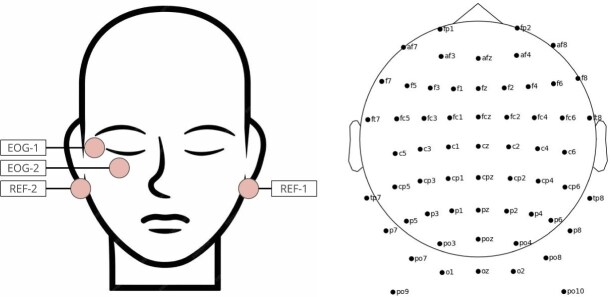
Electrode placement positions for recording EEG and EOG signal.

The FPz electrode was used as the ground electrode. Signals from the left and right earlobes were averaged and applied to other channels as references. Throughout the experiment, electrode impedance was maintained below 30 $k\Omega$, and conductive gel was applied as needed. Electrode placement is depicted in Fig. 3.

### EOG signals

The two electrodes described in the previous EEG subsection were dedicated to recording electrooculography signals. One electrode was placed on the right temple (designated as EOG-1, or hEOG, using channel #61), while the other was positioned on the right infraorbital region (designated as EOG-2, or vEOG, using channel #62), as illustrated in Fig. 3. In this study, EOG signals were used to assist in ocular artifact removal during EEG data preprocessing.

### EMG signals

**Figure 4:**
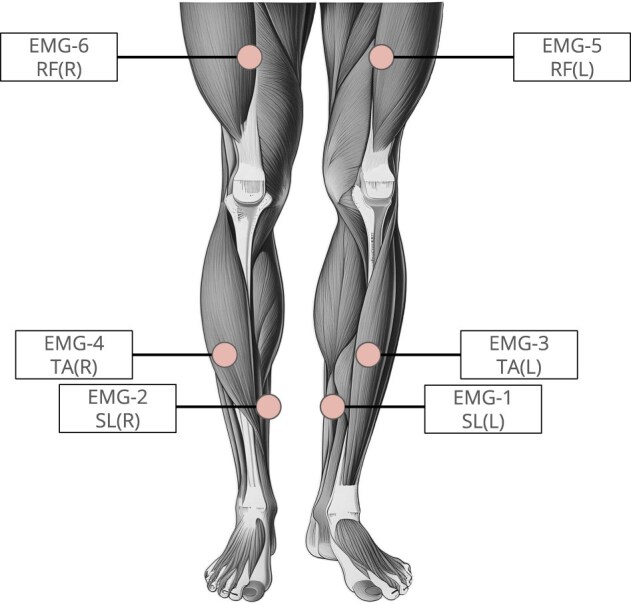
Surface EMG sensor placement positions for recording EMG signal.

Electromyography signals were collected using six surface EMG sensors (Trigno Avanti Sensor, Delsys, USA) at a sampling rate of 2,000 Hz. Sensors were attached bilaterally to three lower-limb muscles: the Soleus (SL), Tibialis Anterior (TA), and Rectus Femoris (RF). The locations of EMG recording sensors are illustrated in Fig. 4. In this study, EMG signals are used to assist in locating the movement onset of ME activities.

### Data synchronization

All recorded physiological signals (EEG, EOG, and EMG) were synchronized with timestamps generated by the data acquisition system, operating on the Windows 11 operating system, prior to preprocessing and analysis.

### Data format and structure

To enhance usability, both raw data in *.mat* format and pre-processed data in *.fif* format are provided. It is recommended to use the Python-based MNE library [9] for full access to *.fif* files. A detailed description of the datasets is presented in Table 1. This study focuses on EEG recordings collected while participants engaged in various BCI motor-related activities, including motor execution, motor imagery during sit, and MI during stand. Additionally, baseline EEG recordings were acquired during resting-state conditions, specifically during eyes-closed and eyes-opened , prior to the execution of motor tasks. These baseline recordings are included to facilitate further analysis. According to Fig. 2a, each experimental session consisted of a single run for ME, EC, and EO. In contrast, each MI condition—MI during sit and MI during stand—was recorded twice per session. Consequently, two separate files were generated for each session of MI during sit, and MI during stand, labeled as *_S1* and *_S2*. The raw EEG data for each participant are structured as a matrix of dimensions $n\_channels \times n\_timepoints$. An extra channel (channel #63) is allocated for event triggers that annotate instruction-related events and serve as ground truth labels. The processed EEG data are structured as a three-dimensional matrix of size $n\_trials \times n\_channels \times n\_timepoints$. A detailed description of event trigger codes is provided in Table 2.

**Table 1:** Data description table.

Name	Description
**Raw data**	
S<*ID*>_S<$session_{num}$ >.mat	Raw signal recorded from subject *ID* in session $session_{num}$
S01_S1.mat	*Example:* Raw signal recorded from subject 01 in session 1
**Processed data**	
S<*ID*>.fif	Pre-processed signal from subject *ID*.
S01.fif	Pre-processed signal from subject 01.

**Table 2:** Event trigger number description table.

Event	Description
1	Eyes closed (beginning of session)
2	Eyes opened (beginning of session)
10	Start of trials in ME activity
11	Start of ME_SIT_STD in ME activity
12	Start of ME_STD_SIT in ME activity
13	Start of resting task (ME_R) in ME activity
20	Start of trials in MI during sit
21	Start of MI_SIT_STD in MI during sit
22	Start of MI_SIT_SIT task in MI during sit
23	Start of resting task in MI during sit (MI_R_SIT, rest while sitting)
30	Start of trials in MI during stand
31	Start of MI_STD_STD task in MI during stand
32	Start of MI_STD_SIT in MI during stand
33	Start of resting task in MI during stand (MI_R_STD, rest while standing)

The event trigger is stored at EEG channel number #63 in the.mat file and embedded into MNE Epochs from the.fif file.

## Data validation

### Data preprocessing

This study’s EEG recordings were preprocessed to analyze brain activity during EC, EO, MI, and ME activities. Specifically, movement-related cortical potential (MRCP) features were examined, predominantly associated with ME activities. Conversely, time-frequency power distribution features were analyzed for EC, EO, and MI activities, as recommended and validated in previous studies [4, 10]. Due to these differences, the preprocessing pipelines for these activities varied slightly, as described in detail below. All preprocessing steps were conducted using the *MNE-Python* library [9].

#### Preprocessing steps for movement-related cortical potential analysis

For MRCP feature extraction in ME-based classification, the following preprocessing steps were applied:

The 60-channel EEG recordings were first filtered with a second-order Butterworth bandpass filter with a 0.2 to 3 Hz cutoff frequency. Subsequently, the signals were downsampled to 250 Hz. To remove artifacts, independent component analysis (ICA) was performed to decompose independent components (ICs) and eliminate artifacts using *mne.preprocessing.ICA* in *MNE-Python* library. To assist the ICA process, the eye-related artifacts were eliminated using recorded EOG signals, while the muscle-related artifacts were eliminated using the function *find_bads_muscle()*. Following, signals from identified bad channels were removed and interpolated using data from neighboring electrodes. To mitigate volume conduction effects and enhance spatial resolution, the current source density (CSD) transformation was applied [11]. Finally, the preprocessed signals were segmented into 4-second epochs ranging from −2 to 2 seconds relative to an onset of EMG trigger event (indicating $T=0$). (Note: The EMG data from session #2 of subject #20 is unavailable. To resolve this issue, specifically for this circumstance, the 4-second epochs were segmented using the onset of the event triggers #11 and #12 obtained from channel number #63.) Furthermore, we excluded those trials contaminated by noise and amplitude spikes with a trial rejection based on peak-to-peak (PTP) amplitude calculation of EEG signals, as those trials with large PTP amplitudes indicate the presence of artifacts. Any trials with the PTP amplitudes exceeding this $95^{th}$ percentile threshold are automatically flagged and rejected from further analysis. On average, 36 trials remained after rejection. Thus, the first 36 trials were selected for further analysis.

For ME-based classification, each trial was labeled according to a participant’s physical movement, either ME activities or resting task (ME_R). The ME resting trials are alternately separated into ME_R_SIT (rest while sitting) and ME_R_STD (rest while standing), respectively. Start from index 0, the even-numbered trials are labeled as ME_R_STD, while the odd-numbered trials are labeled as ME_R_SIT. Similarly, trials labeled as ME_SIT_STD and ME_STD_SIT correspond to a participant performing a sit-stand and stand-sit transition, respectively.

#### Preprocessing steps for time-frequency distribution analysis

Preprocessing for MI-based classification, including EC and EO, was similar to that used for MRCP analysis, with the following modifications:

Instead of using a second-order Butterworth bandpass filter (0.2–3 Hz), a sixth-order Butterworth bandpass filter with 1–40 Hz cutoff frequencies was applied. The processed signals were also segmented into trials ranging from –2 to 5 seconds relative to the event trigger onset.

In contrast to the ME task, the MI task did not involve any actual movement; therefore, no EMG onset was observed. The preprocessed signals were segmented into epochs relative to the onset of the event trigger $\#21$ and $\#32$, as described in Table 2. Each trial spanned 7 seconds (2 seconds before and 5 seconds after the event trigger onset). In order to minimize the influence of ongoing background activity, a 2-second segment before the event trigger was used for baseline correction, leaving a 5-second segment for further analysis. We excluded those trials contaminated with noise by a threshold-based trial rejection using PTP amplitude calculation, similar to the steps for MRCP analysis. On average, 36 trials remained after rejection and were selected for further analysis. For MI-based classification, trials were labeled according to participants’ current physical states. MI_R_SIT and MI_R_STD corresponded to a participant resting while sitting and standing, respectively. Similarly, trials labeled as MI_SIT_STD and MI_STD_SIT corresponded to the participant imagining sit-stand and stand-sit transitions, respectively.

**Figure 5:**
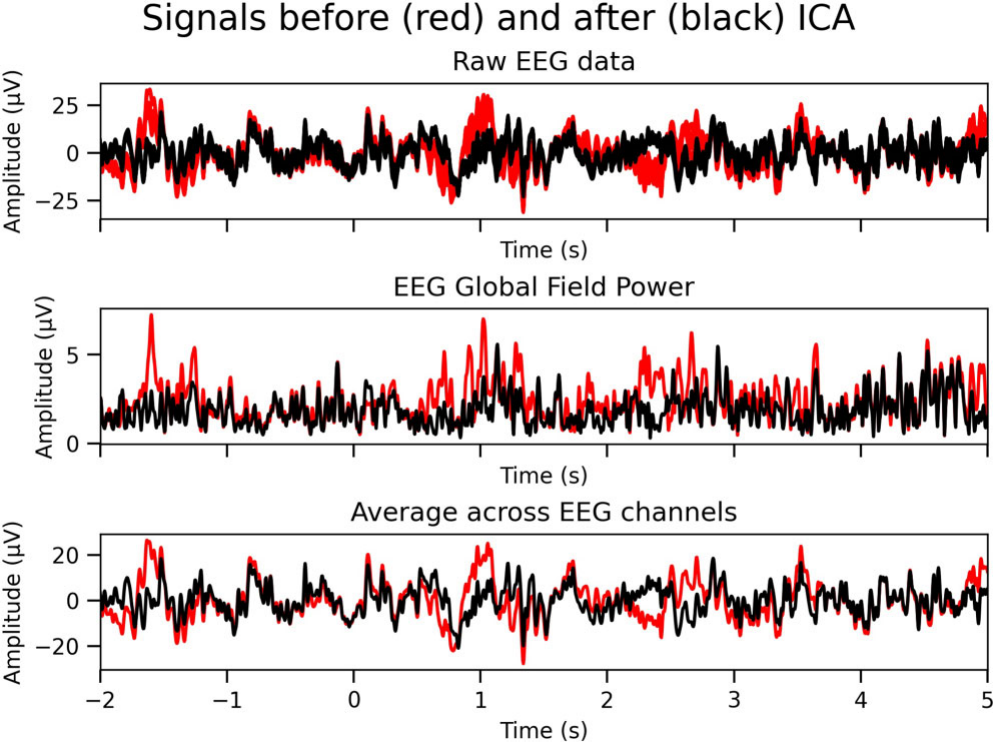
Comparison of EEG signals before (shown in red line) and after (shown in black line) independent component analysis (ICA). The top visualization compares the EEG signals from all channels. The middle visualization compares the EEG global field power from all channels. The bottom visualization compares the average of EEG signals across all channels.

It should be noted that the EEG data preprocessed with ICA and CSD were utilized for visualization, whereas data without these preprocessing steps were used for classification with CTNet, EEGNet, and TCANet models. To confirm the quality of the signals cleaned from the ICA, we compare the signals before and after the cleaning, as shown in Fig. 5. From the figure, we observed that the data after cleaning (shown in black) exhibits lower fluctuation and artifactual components compared to the data before cleaning (shown in red), which exhibits high-frequency components and appears as sharp burst-like waveforms. In addition, our deep learning-based classification experiments were conducted on segmented, filtered EEG time-series data, based on the assumption that the state-of-the-art models are designed in an end-to-end style.

### Method

#### Classification techniques and models

To validate the correctness of the proposed dataset, ME- and MI-based classification experiments were conducted using CTNet[12], EEGNet[13], and TCANet[14] models, the commonly used deep learning baseline models in BCI classification studies. EEG signals, like other biological signals, exhibit high inter-subject variability, making subject-independent classification crucial for broader BCI applications [15]. To avoid subject bias during evaluation, leave-one-subject-out cross-validation (LOSOCV) is employed to ensure the test data remain unseen during training. EEG recordings from each subject were treated as independent samples, enhancing robustness against inter-subject variability. Accordingly, the data input shape to train and validate the model is $n_{subject}-1 \times n_{trial} \times n_{channel} \times n_{timepts}$, while the data input shape to test the model is $1 \times n_{trial} \times n_{channel} \times n_{timepts}$, where $n_{subject}$ represents total number of subjects, $n_{trial}$ represents total number of trials that each subject performed, $n_{channel}$ represents total number of EEG channels, $n_{timepts}$ represents length of EEG samples. All three models were trained using cross-entropy loss for up to 200 epochs, with early stopping triggered if the loss did not improve for 10 consecutive epochs. A 5-fold cross-validation scheme was applied to ensure unbiased and optimal performance. The parameter settings for each model are as follows:

**CTNet** [12]: A convolutional transformer network designed for EEG-based classification of motor imagery. The first layer of CTNet employs a convolutional module to extract local and spatial EEG features. In contrast, the Transformer encoder module is employed in the subsequent layer to learn global dependencies in high-level EEG features. CTNet shows remarkable decoding accuracies for both subject-specific (subject-dependent) and cross-subject evaluations. Our implementation uses Python with *PyTorch* [16], *scikit-learn* [17], and *mne* [9] libraries. In this study, the parameter settings of CTNet are as follows: number of attention heads $n_{head} = 4$, embedding size $emb\_size = 40$, depth $d = 6$, kernel size $k = 64$. The optimal batch size *b* and the learning rate $lr$ are set as $b = 8$ and $lr = 1 \times 10^{-3}$, respectively.

**EEGNet-8,2**: EEGNet [13] effectively learns spatiotemporal EEG features through a compact convolutional neural network. It employs depthwise and separable convolutions to enhance feature learning while reducing trainable parameters, improving efficiency without sacrificing classification performance in EEG-based BCI tasks. Our implementation uses *Python* with *PyTorch* and *scikit-learn*. In this study, the parameter settings of EEGNet-8,2 are as follows: number of filters in the first layer $F1=8$, depth parameter $D=2$, kernel size $C1=200$, number of classes $n_{class}=2$, dropout rate $r_{dropout}=0.5$. The optimal batch size *b* and learning rate $lr$ are set to be $b=8$ and $lr=1\times 10^{-3}$, respectively.

**TCANet** [14]: A multi-scale temporal convolutional attention network designed for EEG-based classification of motor imagery. The first layer adopts a multi-scale convolutional module to extract local spatiotemporal features across different temporal resolutions. Subsequently, the temporal convolutional module combines and compresses these multi-scale features. Finally, the multi-head self-attention mechanism learns global dependency features in the EEG. Our implementation uses Python with *PyTorch, scikit-learn*, and *mne* libraries. In this study, the parameter settings of TCANet are as follows: filter size $f_1 = 16$, pooling size $pooling = 56$, number of attention heads $n_{head} = 2$, depth $d = 6$, dropout rate $r_{dropout} = 0.25$. The optimal batch size *b* and learning rate $lr$ are set to be $b = 8$ and $lr = 1 \times 10^{-3}$, respectively.

### Experiments

This study presents BCI classification in two experimental scenarios—ME ad MI—as the analysis approaches are varied.

#### Experimental design for experiment 1: the MRCP analysis for ME-based classification

For ME-based classification, this study focuses on analyzing and decoding the subjects’ movement intention from movement-related cortical potential, a low-frequency (less than 3 Hz) cortical potential characterized by a negative shift in the EEG signal. MRCP is observed before the onset of real movement and used to detect motor intention, with a direct association to primary motor and somatosensory cortices [18, 19]. As the MRCP is a premovement cortical potential, it usually occurs within 2 seconds prior to movement onset. Accordingly, the 2-second time windows before movement were selected to capture pre-movement brain activity and to evaluate their predictive performance for classification.

In this study, we concentrate on classifying the ME task during transition and resting task using premovement signals. Specifically, we classify EEG recordings in two tasks: (1) ME_SIT_STD vs. ME_R_SIT and (2) ME_STD_SIT vs. ME_R_STD. To determine the optimal EEG segment length for further applications, we trained the three baseline models on two distinct EEG time windows: one second before movement and two seconds before movement. For example, the data input shape for this binary-classification experiment for 2-second data is $22 subjects \times 72 trials\times 60 channels\times 500 timepts$.

#### Experimental design for experiment 2: The time-frequency analysis for MI-based classification

For MI-based classification, this study analyzes and decodes subjects’ movement imagination from the EEG time–frequency distribution in the 1–40 Hz range. We classify EEG recordings in two tasks: (1) MI_SIT_STD vs. MI_R_SIT, (2) MI_STD_SIT vs. MI_R_STD. To study the optimal EEG segment length and evaluate its predictive performance for classification for further applications, we trained and evaluated on five distinct EEG time windows: 1, 2, 3, 4, and 5 seconds after stimulus onset. The example of data input shape for this binary-classification experiment for 5-second data is $22 subjects \times 72 trials\times 60 channels\times 1250 timepts$.

#### Performance matrix and evaluation

In this study, classification performance is assessed and reported in terms of accuracy (ACC) to measure the correct classification rate, F1-score (F1) to assess the balance between precision and recall, and area under the curve (AUC) that provides useful insights on the model’s ability to handle robustness of class-imbalance classification. For insights on the classifier’s performance, confusion matrices of the best model are also provided.

#### Qualitative analysis

In addition to the reported classification performance, to analyze activation of EEG spatially in response to the onset of the visual stimulus, we have visualized topographical maps of transition against resting task in MI activity across different tasks and frequency rhythms, as presented in the upcoming section. These visualizations provide readers with additional insights into how the brain state changes over different time intervals compared with the baseline. Additionally, to select the suitable interval for motor classification, experiments on variations of EEG segment length provide useful insights to assist readers in further studies and analysis.

### Results and discussion

#### Performance analysis

This section presents classification performance for both ME and MI activities. For ME activity, we focused on classifying EEG signals obtained from two tasks—ME_SIT_STD vs. ME_R_SIT (classifying EEG signals between motor execution during sit-stand transition and rest while sitting) and ME_STD_SIT vs. ME_R_STD (classifying EEG signals between motor execution during stand-sit transition and rest while standing). To assess the robustness of the dataset, we evaluated performance across different EEG segment lengths using three baseline deep learning models commonly applied in BCI studies: CTNet, EEGNet, and TCANet.

We evaluated our dataset by assessing the performance of three baseline models in terms of classification results and running time, illustrated in Table 3. It can be seen that TCANet generally performs substantially better than the other methods. In ME_SIT_STD vs. ME_R_SIT task, TCANet attained the best accuracy of $81.15\pm 8.36\%$, $79.81\pm 10.38\%$ in terms of F1-score, and $0.8958\pm 0.0700$ in terms of AUC, on the 2-second segment, with the average training time $192.74\pm 19.02$ seconds per one fold and average inference time $0.10\pm 0.00$ second. In ME_STD_SIT vs. ME_R_STD task, TCANet also attained the best accuracy of $80.86\pm 8.83\%$, $79.65\pm 11.44\%$ in terms of F1-score, and $0.8993\pm 0.0770$ in terms of AUC, on the 2-second segment, with the average training time $216.13\pm 24.89$ seconds per one fold and average inference time $0.10\pm 0.00$ second.

**Table 3:** Classification performance of three baseline methods (CTNet, EEGNet, TCANet), along with training $T_{train}$ and inference $T_{infer}$ times (in seconds) per one fold, on the proposed dataset for motor execution (ME) and motor imagery (MI) tasks using different EEG segment lengths (Mean $\pm$ SD).

Experiment	Task	Segment Length (s)	Accuracy $\uparrow$	F1-score $\uparrow$	AUC $\uparrow$	$T_{train}$ (s) $\downarrow$	$T_{infer}$ (s) $\downarrow$
**Method: CTNet**							
ME	ME_SIT_STD vs. ME_R_SIT	1	$77.35 \pm 8.78*$	$74.37 \pm 12.34*$	$0.8570 \pm 0.0939*$	$\mathbf {413.00 \pm 37.31}$	$\mathbf {0.39 \pm 0.04}$
		2	$\mathbf {81.25 \pm 8.30}$	$\mathbf {80.34 \pm 9.64}$	$\mathbf {0.8996 \pm 0.0766}$	$610.73 \pm 488.64*$	$0.59 \pm 0.63$
	ME_STD_SIT vs. ME_R_STD	1	$77.60 \pm 9.57*$	$74.95 \pm 13.32$	$0.8643 \pm 0.0894*$	$\mathbf {410.64 \pm 41.86}$	$\mathbf {0.39 \pm 0.07}$
		2	$\mathbf {79.89 \pm 9.52}$	$\mathbf {78.98 \pm 11.00}$	$\mathbf {0.8912 \pm 0.0903}$	$602.05 \pm 493.91*$	$0.60 \pm 0.58*$
MI	MI_SIT_STD vs. MI_R_SIT	1	$71.41 \pm 8.48$	$68.31 \pm 13.53$	$0.7889 \pm 0.0986$	$\mathbf {160.61 \pm 9.20}$	$\mathbf {0.09 \pm 0.00}$
		2	$\mathbf {71.93 \pm 8.30}$	$\mathbf {70.19 \pm 12.01}$	$0.8053 \pm 0.0903$	$164.70 \pm 11.78$	$0.09 \pm 0.00*$
		3	$71.23 \pm 8.81$	$69.74 \pm 13.63$	$0.8082 \pm 0.0987$	$241.26 \pm 16.86*$	$0.23 \pm 0.02*$
		4	$70.31 \pm 9.80$	$68.29 \pm 16.37$	$0.7986 \pm 0.1073$	$258.53 \pm 19.47*$	$0.24 \pm 0.02*$
		5	$70.77 \pm 8.45$	$68.69 \pm 13.98$	$\mathbf {0.8136 \pm 0.0921}$	$2566.27 \pm 134.43*$	$3.89 \pm 0.46*$
	MI_STD_SIT vs. MI_R_STD	1	$72.79 \pm 8.23$	$70.24 \pm 12.43$	$0.8073 \pm 0.0829$	$\mathbf {159.37 \pm 8.15}$	$\mathbf {0.09 \pm 0.00}$
		2	$72.80 \pm 8.32$	$70.48 \pm 13.51$	$0.8157 \pm 0.0874$	$162.18 \pm 9.88$	$0.09 \pm 0.00*$
		3	$\mathbf {73.08 \pm 10.15}$	$\mathbf {70.56 \pm 17.15}$	$\mathbf {0.8240 \pm 0.0914}$	$240.23 \pm 13.91*$	$0.23 \pm 0.03*$
		4	$71.95 \pm 11.23$	$69.24 \pm 18.39$	$0.8209 \pm 0.1045$	$256.81 \pm 20.99*$	$0.23 \pm 0.02*$
		5	$71.55 \pm 11.49$	$67.35 \pm 18.94$	$0.8125 \pm 0.1073$	$2454.36 \pm 139.27*$	$3.63 \pm 0.51*$
**Method: EEGNet**							
ME	ME_SIT_STD vs. ME_R_SIT	1	$75.91 \pm 9.38$ *	$70.87 \pm 14.84$ *	$0.8210 \pm 0.1091$ *	$70.01 \pm 9.33$ *	$0.06 \pm 0.01$
		2	$\mathbf {79.85 \pm 7.99}$	$\mathbf {77.60 \pm 10.47}$	$\mathbf {0.8844 \pm 0.0751}$	$\mathbf {63.37 \pm 7.63}$	$\mathbf {0.06 \pm 0.01}$
	ME_STD_SIT vs. ME_R_STD	1	$76.02 \pm 9.68$ *	$71.68 \pm 15.62$ *	$0.8302 \pm 0.0984$ *	$\mathbf {61.40 \pm 5.53}$	$0.06 \pm 0.01$
		2	$\mathbf {79.68 \pm 8.80}$	$\mathbf {77.93 \pm 11.88}$	$\mathbf {0.8866 \pm 0.0836}$	$63.22 \pm 6.65$	$\mathbf {0.06 \pm 0.01}$
MI	MI_SIT_STD vs. MI_R_SIT	1	$69.16 \pm 8.62$	$67.48 \pm 13.28$	$0.7677 \pm 0.0981$	$\mathbf {86.96 \pm 12.95}$	$0.08 \pm 0.01$
		2	$\mathbf {69.58 \pm 8.86}$	$69.69 \pm 11.15$	$0.7994 \pm 0.0973$	$92.32 \pm 15.79$	$\mathbf {0.07 \pm 0.01}$
		3	$68.98 \pm 9.56$	$\mathbf {70.70 \pm 11.84}$	$\mathbf {0.8045 \pm 0.0975}$	$155.97 \pm 27.96$ *	$0.14 \pm 0.03$ *
		4	$67.85 \pm 10.24$	$69.58 \pm 12.91$	$0.7992 \pm 0.1016$	$168.59 \pm 32.24$ *	$0.13 \pm 0.04$ *
		5	$67.39 \pm 10.25$	$69.16 \pm 14.02$	$0.7985 \pm 0.1031$	$167.96 \pm 25.68$ *	$0.14 \pm 0.03$ *
	MI_STD_SIT vs. MI_R_STD	1	$71.37 \pm 7.44$	$68.73 \pm 13.24$	$0.7872 \pm 0.0969$	$\mathbf {83.03 \pm 11.12}$	$0.07 \pm 0.01$
		2	$71.27 \pm 8.33$	$\mathbf {70.15 \pm 13.16}$	$0.8022 \pm 0.1001$	$95.65 \pm 13.74$ *	$\mathbf {0.07 \pm 0.01}$
		3	$70.88 \pm 10.82$	$68.77 \pm 19.01$	$0.8080 \pm 0.1204$	$124.96 \pm 24.34$ *	$0.08 \pm 0.03$
		4	$\mathbf {71.42 \pm 11.13}$	$68.58 \pm 20.98$	$\mathbf {0.8149 \pm 0.1129}$	$149.74 \pm 19.01$ *	$0.07 \pm 0.01$
		5	$70.63 \pm 11.54$	$67.51 \pm 21.92$	$0.8049 \pm 0.1215$	$148.15 \pm 14.33$ *	$0.07 \pm 0.02$
**Method: TCANet**							
ME	ME_SIT_STD vs. ME_R_SIT	1	$77.93 \pm 8.95$	$74.44 \pm 12.70$	$0.8532 \pm 0.0987*$	$230.21 \pm 31.27*$	$\mathbf {0.09 \pm 0.00}$
		2	$\mathbf {81.15 \pm 8.36}$	$\mathbf {79.81 \pm 10.38}$	$\mathbf {0.8958 \pm 0.0700}$	$\mathbf {192.74 \pm 19.02}$	$0.10 \pm 0.00*$
	ME_STD_SIT vs. ME_R_STD	1	$78.69 \pm 9.88$	$74.84 \pm 15.55$	$0.8628 \pm 0.0885$	$237.31 \pm 25.10*$	$\mathbf {0.09 \pm 0.01}$
		2	$\mathbf {80.86 \pm 8.83}$	$\mathbf {79.65 \pm 11.44}$	$\mathbf {0.8993 \pm 0.0770}$	$\mathbf {216.13 \pm 24.89}$	$0.10 \pm 0.00*$
MI	MI_SIT_STD vs. MI_R_SIT	1	$70.48 \pm 9.61$	$67.88 \pm 15.24$	$0.7776 \pm 0.1059*$	$\mathbf {300.23 \pm 42.52}$	$\mathbf {0.10 \pm 0.00}$
		2	$\mathbf {72.06 \pm 7.87}$	$70.24 \pm 11.43$	$0.8090 \pm 0.0893$	$308.52 \pm 35.18$	$0.10 \pm 0.01*$
		3	$71.66 \pm 8.83$	$\mathbf {70.47 \pm 13.13}$	$\mathbf {0.8222 \pm 0.0899}$	$446.30 \pm 54.60*$	$0.24 \pm 0.02*$
		4	$70.77 \pm 9.73$	$69.40 \pm 15.25$	$0.8162 \pm 0.0908$	$416.40 \pm 49.81*$	$0.25 \pm 0.02*$
		5	$69.98 \pm 9.11$	$68.70 \pm 15.46$	$0.8102 \pm 0.0906*$	$397.43 \pm 69.92*$	$0.24 \pm 0.04*$
	MI_STD_SIT vs. MI_R_STD	1	$73.25 \pm 8.34$	$71.78 \pm 11.84$	$0.8113 \pm 0.0940$	$313.94 \pm 39.21$	$\mathbf {0.09 \pm 0.00}$
		2	$72.92 \pm 7.58$	$71.88 \pm 10.91$	$0.8162 \pm 0.0885$	$\mathbf {312.73 \pm 35.05}$	$0.10 \pm 0.00*$
		3	$\mathbf {73.60 \pm 9.33}$	$\mathbf {72.12 \pm 14.28}$	$0.8354 \pm 0.0897$	$439.43 \pm 91.45*$	$0.22 \pm 0.06*$
		4	$73.21 \pm 10.94$	$70.98 \pm 17.59$	$0.8388 \pm 0.0948$	$447.48 \pm 57.71*$	$0.24 \pm 0.02*$
		5	$73.53 \pm 10.52$	$71.23 \pm 17.18$	$\mathbf {0.8389 \pm 0.0969}$	$419.95 \pm 50.11*$	$0.24 \pm 0.02*$

Note: * indicates statistically significant difference using a *t*-test ($P \le 0.05$) compared to the best-performing setting, highlighted in bold. Segment length refers to the EEG window duration in seconds, extracted relative to stimulus onset (before onset for ME, after onset for MI).

Abbreviations: ME_SIT_STD, executing standing up from sitting; ME_R_SIT, rest while sitting; ME_STD_SIT, executing sitting down from standing; ME_R_STD, rest while standing; MI_SIT_STD, imagining standing up from sitting; MI_R_SIT, rest while sitting; MI_STD_SIT, imagining sitting down from standing; MI_R_STD, rest while standing; $T_{train}$, training time; $T_{infer}$, inference time (testing time).

Similarly, EEG signals from MI activity were classified for two binary tasks: MI_SIT_STD vs. MI_R_SIT (movement imagination of sit-stand vs. rest while sitting) and MI_STD_SIT vs. MI_R_STD (movement imagination of stand-sit vs. rest while standing). To assess robustness, we evaluated performance across varying EEG segment lengths using the TCANet model. For MI_SIT_STD vs. MI_R_SIT, TCANet network achieved its best accuracy of $72.06\pm 7.87\%$ with the 2-second segment, while the best F1-score and AUC of $70.47 \pm 13.13\%$ and $0.8222 \pm 0.0899$, respectively, were obtained with the 3-second segment. Moreover, the optimal training and inference times of $300.23 \pm 42.52$ and $0.10 \pm 0.00$ seconds, respectively, were achieved with the 1-second segment. For MI_STD_SIT vs. MI_R_STD, TCANet achieved the best accuracy of $73.60 \pm 9.33\%$ and F1-score of $72.12 \pm 14.28\%$ with the 3-second segment, while the AUC of $0.8389 \pm 0.0969$, optimal training of $312.73 \pm 35.05$ seconds, and inference times of $0.09 \pm 0.00$ seconds were obtained with the 5-second segment.

**Figure 6:**
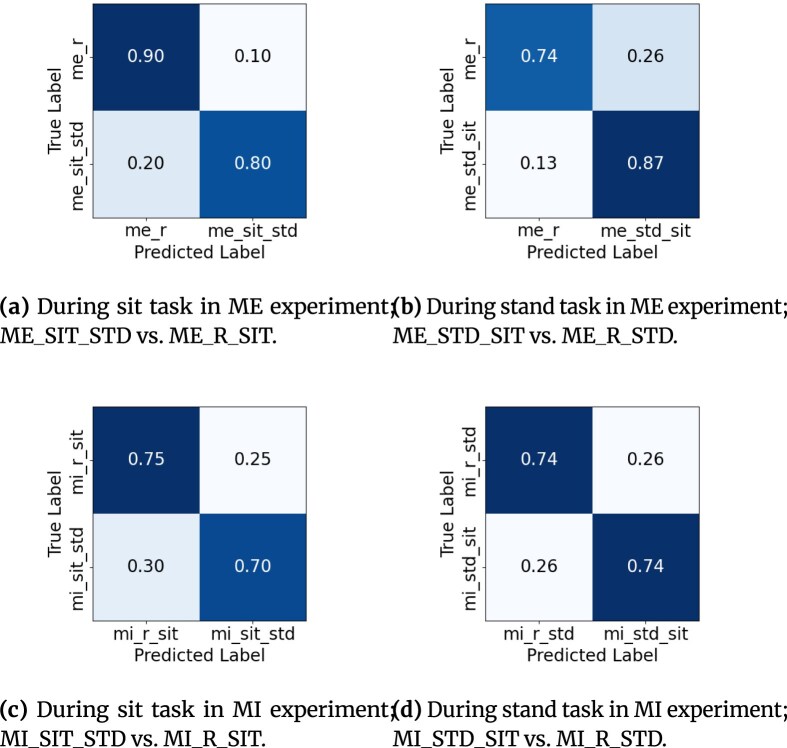
Confusion matrices of the proposed dataset for motor execution (ME) and motor imagery (MI) tasks using TCANet model.

In addition, the confusion matrices for ME- and MI-based classification on the proposed dataset using the TCANet model are presented in Fig. 6.

#### Qualitative analysis

**Figure 7:**
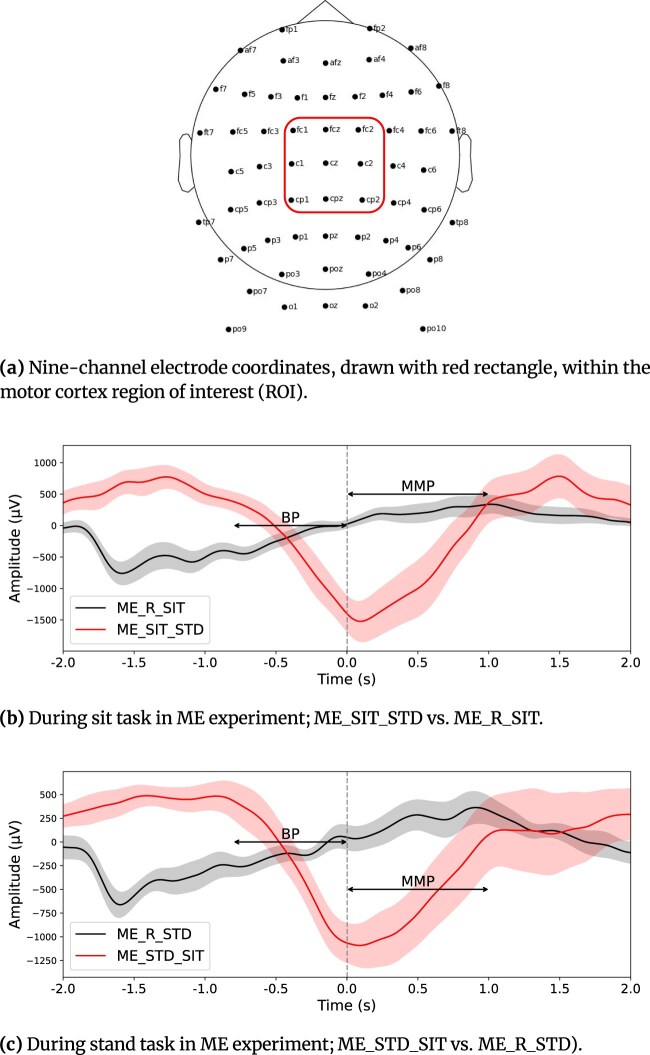
Grand-average EEG activity (with standard error) of nine motor cortex electrodes during ME experiment.

This study also performed qualitative analyses of the recorded EEG signals to confirm the correctness and enhance the explainability of the obtained data. In the ME activity, we focus on analyzing the EEG signals in terms of MRCPs, spontaneous potentials generated during person-generated movement [4, 20]. Figure 7 shows the grand average of EEG signals during ME activity for both sit and stand tasks across all subjects. The visualizations are obtained from the region of interest of 9 electrodes around the motor cortex area, in accordance with [21], including FC1, FCz, FC2, C1, Cz, C2, CP1, CPz, CP2, as shown in Fig. 7a. These nine-electrode channels were selected based on their correspondence to the scalp projection of the lower-limb (foot) representation within the primary motor cortex (M1) area, consistent with the established somatotopic organization along the precentral gyrus [22]. This anatomical relevance is further supported by the topographic distribution illustrated in [23]. During the sit task (sit-stand, or ME_SIT_STD), it is observed from Fig. 7b that, comparing to rest while sitting (ME_R_SIT), Bereitschaftspotential (BP, also known as readiness potential) exhibits slow negative EEG about 0.8 seconds (–0.8 to 0 second) before the actual onset of movement (designated by dotted gray vertical line) where the peak negativity lies around 0 second. Movement-monitoring potential (MMP), a component of MRCPs that reflects brain activity after the physical execution of a voluntary movement, relates to the brain’s monitoring of the movement precision and control, lasting about 1 second after the movement onset.

On the other hand, for the stand task (stand-sit, or ME_STD_SIT), it is observed from Fig. 7c that, compared to the rest while standing (ME_R_STD), the BP potential starts around 0.8 seconds (–0.8 second) before the actual onset of movement, gradually decreasing to the peak negativity around 0.25 seconds. The MMP lasts about 1 second after the onset of physical movement.

A small latency in the negative peak of MRCP signals is observed in ME stand-sit task (ME_STD_SIT), compared to ME sit-stand task (ME_SIT_STD). This could be related to the asymmetry of the neural preparatory process between these two tasks. The ME_SIT_STD task is considered to be a propulsive movement, requiring a rapid generation of force against gravity and inertia. This task demands a strong and immediate motor output which may requires a more decisive and rapid cortical preparation. Conversely, the ME_STD_SIT task involves a controlled descent, which requires precise and continuous modulation of muscle activity to decelerate the body and ensure a smooth, stable sitting. This control might involve a more prolonged preparatory phase, potentially delaying the MRCP negative peak [20].

**Figure 8:**
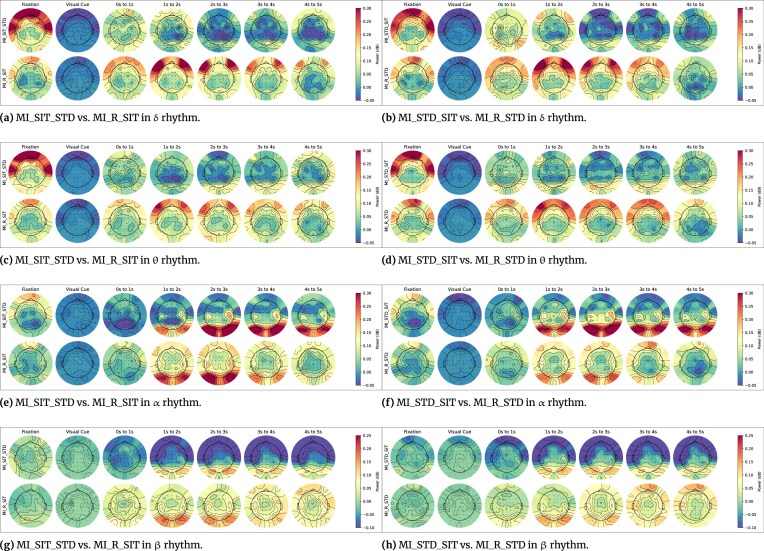
Topographical map visualizations for grand average of spectral power in $\delta$ (1–4 Hz), $\theta$ (4–8 Hz), $\alpha$ (8–13 Hz), and $\beta$ (13–30 Hz) rhythms, calculated from the Morlet wavelet across 7 seconds (2 seconds of fixation and visual cue/visual stimulus and 5 seconds window after the visual stimulus onset) during various motor imagery (MI) activities. This includes MI_SIT_STD (motor imagery during sit to stand, or standing up from sitting on a chair) vs. MI_R_SIT (resting while sitting on a chair) and MI_STD_SIT (motor imagery during stand to sit, or sitting on a chair from standing up) vs. MI_R_STD (resting while standing).

For the MI activity, we analyze EEG signals in the time–frequency domain, i.e., how spectral components evolve over time. Figure 8 presents topographical maps of spectral power in the $\delta$ (1–4 Hz), $\theta$ (4–8 Hz), $\alpha$ (8–13 Hz), and $\beta$ (13–30 Hz) bands, visualized for each second across a 5-second window. In terms of ERD/ERS, we observe pronounced ERD—a decrease in the power of an EEG rhythm—in frontal and central regions during the transition periods of both sit and stand motor-imagery tasks. This ERD is evident in the $\delta$, $\theta$, and $\alpha$ bands and is particularly strong in the $\beta$ band, consistent with increased cortical activation and sensorimotor information processing during movement-related transitions.

On the other hand, we observe strong event-related synchronization (ERS)—an increase in the power of an EEG rhythm—primarily in the parietal region during resting (non-movement) periods, especially in the $\beta$ band. In the $\delta$, $\theta$, and $\alpha$ bands, pronounced ERS is also evident in the parietal and frontal regions during resting. These observations are consistent with the findings reported by [24].

#### Discussion

This study presents and evaluates the performance of an EEG-based dataset, focusing on neural activation patterns of the sitting and standing conditions during MI and ME. As EEG has high variance across individuals [15], to support a variety of future applications on motor tasks, we train, validate, and test on a cross-subject (or subject-independent) basis.

In the ME activity, we present the classification performance for EEG signals obtained during the transition and resting periods. Since the classification focuses on training the model to learn MRCP features, we compare the models’ performance using 1-second and 2-second EEG segments prior to movement onset for training and testing. From the results shown in Table 3, we see that the results of the 2-second EEG segment from all three models give the best classification performance and training time. Considering ME_SIT_STD vs. ME_R_SIT, although the 1-second EEG segment before the movement onset gives acceptable performances for at least 77.93% on accuracy when classified with TCANet, using the 2-second EEG segment provides significantly improved performance up to at least 81.15%, with around 193 seconds of training time. This is related to the ability of the classifier to learn a longer window of BP components. Compared to the resting EEG segments, we observe from Fig. 7 that longer segments clearly show the BP components of ME activity related to the cortical excitability and readiness for the movement [25, 26].

In the MI activity, we present the classification performance for EEG signals obtained during the transition and resting periods. This classification task focuses on training the model to learn time-frequency features. We compare the models’ performance across 1-second, 2-second, 3-second, 4-second, and 5-second EEG segments, starting from the visual stimulus onset, for both training and testing. From the results shown in Table 3, we see that the results of the 2-second EEG segments generally give the optimal performance in terms of classification performance and training time. For MI_SIT_STD vs. MI_R_SIT (sit-stand motor imagery vs. sit-rest), using the 2-second EEG segment after stimulus onset generally gives the optimal performance: 72.06% accuracy and 308.52 seconds of training time with TCANet. Similarly, for MI_STD_SIT vs. MI_R_STD (stand-sit motor imagery vs. stand-rest) tasks, although the best performance was obtained with the 3-second EEG segment, considering a balance of trade-offs on the segment length, computation time, and a little performance gain, the 1-second MI EEG segment seemed to be a good option to use. The reported training time is 313.94 seconds with 73.25% accuracy. These results are in accordance with the results reported by [27, 28] on lower-limb motor imagery classification.

To support this, Fig. 8 shows topographical map visualizations for the grand average of spectral power in $\delta$, $\theta$, $\alpha$, and $\beta$ rhythms on 7-second MI EEG intervals (2 seconds of fixation and visual stimulus and 5 seconds after the visual stimulus onset). For both MI_SIT_STD vs. MI_R_SIT and MI_STD_SIT vs. MI_R_STD conditions, the differences in EEG distributions can be observed from the figure at 2 seconds after the onset of the visual stimulus. These visualizations correspond to the classification results previously reported in Table 3, ensuring the optimal selection of EEG segment lengths.

Compared to the existing studies on lower-limb-movement MI EEG datasets, our study targets transitional movement dynamics in sitting and standing movement initiation tasks. The goal of our study is to enable the exploration of neural activation patterns related to movement for supporting the development of BCI algorithms for mobility assistance and neural rehabilitation. To support this, our study includes high-density EEG recordings and tasks from ME and MI activity, enabling investigation of neural dynamics during movement transitions.

Although the highest mean classification accuracies for lower-limb MI EEG during sit-stand tasks have been reported at up to 88.51% in offline analysis and 96.56% in online analysis [5], the validation approach employed in that study raises concerns regarding practical applicability. Specifically, the researchers developed individual machine learning models tailored to each subject (within-subject or subject-dependent classification), rather than training a generalized model on a group of subjects and evaluating it on unseen subjects (cross-subject or subject-independent classification). This reliance on subject-dependent models may limit the applicability of such systems in real-world medical applications. In contrast, our study adopts a subject-independent validation framework to enhance generalizability across users. While this approach is inherently more challenging due to high inter-subject variability in EEG signals, it better reflects the conditions of real-world deployment.

#### Limitations

While this study provides a foundational characterization of movement-related neural signatures, it is important to acknowledge that this dataset is obtained from healthy young adults, which presents a significant limitation for direct clinical translation, particularly for patients such as stroke survivors. Research indicates that movement-related neural dynamics—specifically the amplitude and latency of MRCPs as well as event-related desynchronization/synchronization (ERD/ERS) patterns—undergo substantial changes due to both aging and neurological impairment [29–32]. These physiological differences create a domain shift, where models trained on healthy cohorts may fail to generalize effectively when applied to clinical cohorts for rehabilitation purposes [33, 34].

To successfully bridge the gap between these experimental findings and practical clinical applications, several mitigation strategies are required. Effective translation will depend on incorporating specialized knowledge of EEG characteristics related to lower-limb motor tasks unique to impaired populations into model design. Furthermore, technical approaches such as transfer learning, feature alignment, meta-learning, fine-tuning with condition-specific data, and applying domain adaptation will be essential to account for the altered signal distributions in clinical groups. Finally, future work may require external validation in cohorts aligned with specific rehabilitation scenarios to ensure these models remain robust and reliable in a real-world context.

Although this study employs deep learning algorithms for conducting experiments for dataset validation, this dataset is intended to be applicable for both conventional machine learning and deep learning-based lower-limb motor task classification methods. As training the deep learning model requires a large sample set size, data augmentation techniques on EEG signals in motor imagery tasks, such as those studies by [35–37], may be recommended to mitigate data scarcity concerns, providing methodological flexibility for future investigation.

## Conclusion

This study introduces a novel EEG dataset focused on sitting and standing transitions during both motor execution and motor imagery tasks. The data, including 60 EEG channels, EOG, and EMG, were obtained from 22 participants who conducted two data recording sessions focused on lower-limb movements. To ensure the usability of the proposed dataset, we have evaluated its performance using three baseline BCI classification algorithms—CTNet, EEGNet, and TCANet—on two tasks—sit-stand vs. sit-rest and stand-sit vs. stand-rest. In addition, we evaluate performance across various EEG segment lengths to suggest further BCI applications. This dataset lays a solid foundation for the development and benchmarking of future BCI algorithms, offering researchers a valuable resource for better understanding and enhancing the performance of MI-based BCIs.

## Availability of source code and requirements

Project name: eeg_sit_stand

Project homepage: https://github.com/b5510546671/eeg_sit_stand

Operating system: Platform independent (tested on Unix-based systems)

Programming language: Python (version 3.8.0)

Other requirements:

- numpy (v2.0.2)
- scipy (v1.13.1)
- scikit-learn (v1.6.1)
- torch (v2.6.0)
- braindecode (v1.3.2)
- mne (v1.8.0)

License: Apache License 2.0

## Abbreviations

EC: eyes-closed resting state; EO: eyes-opened resting state; ERD: event-related desynchronization; ERS: event-related synchronization; ERSP: event-related spectral perturbation; ME: motor execution; ME_R: resting during motor execution; ME_R_SIT: sit-resting during motor execution; ME_R_STD: stand-resting during motor execution; ME_SIT_STD: executing standing up from sitting; ME_STD_SIT: executing sitting down from standing; MI: motor imagery; MI_R: resting during motor imagery; MI_R_SIT: sit-resting during motor imagery; MI_R_STD: stand-resting during motor imagery; MI_SIT_STD: imagining standing up from sitting; MI_SIT_SIT: imagining sitting while sitting; MI_STD_SIT: imagining sitting down from standing; MI_STD_STD: imagining standing while standing; MRCP: movement-related cortical potential.

## Supplementary Material

giag065_Authors_Response_To_Reviewer_Comments_original_submission

giag065_GIGA-D-25-00472_original_submission

giag065_GIGA-D-25-00472_revision_1

giag065_Reviewer_1_Report_original_submission Reviewer 1 -- 12/16/2025

giag065_Reviewer_1_Report_revision_1 Reviewer 1 -- 4/11/2026

giag065_Reviewer_2_Report_original_submission Reviewer 2 -- 12/23/2025

## Data Availability

The raw and processed EEG data supporting the findings of this study are openly available in the Zenodo repository [38] and [39], respectively. The DOME-ML (Data, Optimisation, Model, Evaluation in Machine Learning) annotations in this study have been deposited in the DOME-ML Registry and can be accessed with the persistent identifier wnrszixlqm [40].
