# Supplementary material for: EEG-based dataset explicitly targets the transitions between sitting and standing for exploring neural activation patterns in motor imagery and execution
Source: Gigascience. 2026 May 29;15:giag065. doi: 10.1093/gigascience/giag065 (PMC13270978; doi:10.1093/gigascience/giag065)
Supplement: giag065_GIGA-D-25-00472_original_submission [file giag065_giga-d-25-00472_original_submission.pdf]

GigaScience

EEG-Based Dataset Explicitly Targeting the Transitions between Sitting and Standing for Exploring Neural Activation Patterns in Motor Imagery and Execution

--Manuscript Draft--

Manuscript Number:	GIGA-D-25-00472
Full Title:	EEG-Based Dataset Explicitly Targeting the Transitions between Sitting and Standing for Exploring Neural Activation Patterns in Motor Imagery and Execution
Article Type:	Data Note
Funding Information:	National Science Research and Innovation Fund (NSRF) Thailand (NRIIS Number: 179275) — Prof. Gun Bhakdisongkhram<br>National Science Research and Innovation Fund (NSRF), the Program Management Unit for Human Resources & Institutional Development Research and Innovation Thailand (B13F680099) — Prof. Theerawit Wilaiprasitporn
Abstract:	This study presents the first publicly accessible electroencephalography (EEG) dataset explicitly targeting sit-to-stand and stand-to-sit transitions during both motor execution (ME) and motor imagery (MI) tasks. Twenty-two healthy participants performed sitting and standing transitions under well-controlled experimental conditions while 60-channel EEG, electrooculography (EOG), and electromyography (EMG) signals were synchronously recorded. The dataset enables the exploration of neural activation patterns associated with lower-limb movements and supports the development of EEG-based brain–computer interface (BCI) algorithms for mobility assistance and rehabilitation. To validate the dataset, a benchmark classification was conducted using EEGNet, a compact convolutional neural network. Results demonstrated consistent decoding performance with mean accuracies of approximately 80% for ME and 70% for MI, indicating the reliability and usability of the dataset. Additionally, analyses of movement-related cortical potentials (MRCPs) and event-related desynchronization/synchronization (ERD/ERS) patterns revealed distinct neural signatures across the transition phases. This dataset provides a comprehensive foundation for studying lower-limb motor control, neural dynamics, and the advancement of MI-based BCIs for rehabilitation and assistive technologies.
Corresponding Author:	Theerawit Wilaiprasitporn, Ph.D.<br>Vidyasirimedhi Institute of Science and Technology<br>Rayong, Thailand THAILAND
Corresponding Author Secondary Information:	
Corresponding Author's Institution:	Vidyasirimedhi Institute of Science and Technology
Corresponding Author's Secondary Institution:	
First Author:	Benjakarn Uengsawapak
First Author Secondary Information:	
Order of Authors:	Benjakarn Uengsawapak
	Supavit Kongwudhikunakorn, Ph.D.
	Suktipol Kiatthaveephong
	Wipamas Polpakdee
	Rattanaphon Chaisaen, Ph.D.
	Poramate Manoonpong, Ph.D.
	Chanitsada Chuenchit

Powered by Editorial Manager® and ProduXion Manager® from Aries Systems Corporation

	Gun Bhakdisongkhram, M.D, Ph.D.
	Theerawit Wilaiprasitporn, Ph.D.
**Order of Authors Secondary Information:**	
**Additional Information:**	
**Question**	**Response**
Are you submitting this manuscript to a special series or article collection?	No
**Experimental design and statistics**<br><br>Full details of the experimental design and statistical methods used should be given in the Methods section, as detailed in our Minimum Standards Reporting Checklist. Information essential to interpreting the data presented should be made available in the figure legends.<br><br>Have you included all the information requested in your manuscript?	Yes
**Resources**<br><br>A description of all resources used, including antibodies, cell lines, animals and software tools, with enough information to allow them to be uniquely identified, should be included in the Methods section. Authors are strongly encouraged to cite Research Resource Identifiers (RRIDs) for antibodies, model organisms and tools, where possible.<br><br>Have you included the information requested as detailed in our Minimum Standards Reporting Checklist?	Yes
**Availability of data and materials**<br><br>All datasets and code on which the conclusions of the paper rely must be either included in your submission or deposited in publicly available repositories (where available and ethically appropriate), referencing such data using	Yes

a unique identifier in the references and in the “Availability of Data and Materials” section of your manuscript.<br><br>Have you have met the above requirement as detailed in our Minimum Standards Reporting Checklist?	
GigaScience has policies and guidelines in place for the use of generative AI-writing tools such as ChatGPT. If you have used such writing tools to assist with writing the manuscript this must be declared and cited in the text. Authors should not list AI-writing tools and other AI-assisted technologies as an author or co-author and should acknowledge that they are fully responsible for text generated or refined by AI-writing tools.<p><br><br>A summary of use (particularly in the introduction or among methods) needs to be included at the end of the paper, and the outputs should also be included as a supplementary file hosted in GigaDB or other open repositories. Please read our guidelines for more information. <p><br><br>By submitting to GigaScience, you are aware of the journal's AI-writing tools policy, and if you have declared use of such tools below, you have acknowledged this where appropriate in your manuscript and have made a summary of use and outputs available. <p><br><b>AI-assisted writing tools have been used in the preparation of this manuscript?	No

Powered by Editorial Manager® and ProduXion Manager® from Aries Systems Corporation

Manuscript

Click here to access/download;Manuscript;Benz_s_Paper (2).pdf

GigaScience, 2025, 1–10

doi: xx.xxxx/xxxx
Manuscript in Preparation
Data Note

DATA NOTE

EEG-Based Dataset Explicitly Targets the Transitions between Sitting and Standing for Exploring Neural Activation Patterns in Motor Imagery and Execution

Benjakarn Uengsawapak[1,*], Supavit Kongwudhikunakorn[2,*], Suktipol Kiatthaveephong[2], Wipamas Polpakdee[2], Rattanaphon Chaisaen[2], Poramate Manoonpong[2], Chanitsada Chuenchit[3], Gun Bhakdisongkhram[4,†] and Theerawit Wilaiprasitporn[2,†]

[1]School of Information Science and Technology (IST), Vidyasirimedhi Institute of Science and Technology (VISTEC), Rayong, Thailand,21210 and [2]Bio-inspired Robotics and Neural Engineering (BRAIN) Lab, School of Information Science and Technology (IST), Vidyasirimedhi Institute of Science and Technology (VISTEC), Rayong, Thailand,21210 and [3]Sirindhorn International Institute of Technology, Thammasat University, Pathum Thani, Thailand, 12120 and [4]School of Physical Medicine and Rehabilitation, Institute of Medicine, Suranaree University of Technology, Nakhon Ratchasima, Thailand, 30000

[*]These authors contributed equally to this work.
[†]Corresponding authors: G. Bhakdisongkhram (gunbhak@sut.ac.th) and T. Wilaiprasitporn (theerawit.w@vistec.ac.th)

Abstract

This study presents the first publicly accessible electroencephalography (EEG) dataset explicitly targeting sit-to-stand and stand-to-sit transitions during both motor execution (ME) and motor imagery (MI) tasks. Twenty-two healthy participants performed sitting and standing transitions under well-controlled experimental conditions while 60-channel EEG, electrooculography (EOG), and electromyography (EMG) signals were synchronously recorded. The dataset enables the exploration of neural activation patterns associated with lower-limb movements and supports the development of EEG-based brain–computer interface (BCI) algorithms for mobility assistance and rehabilitation. To validate the dataset, a benchmark classification was conducted using EEGNet, a compact convolutional neural network. Results demonstrated consistent decoding performance with mean accuracies of approximately 80% for ME and 70% for MI, indicating the reliability and usability of the dataset. Additionally, analyses of movement-related cortical potentials (MRCPs) and event-related desynchronization/synchronization (ERD/ERS) patterns revealed distinct neural signatures across the transition phases. This dataset provides a comprehensive foundation for studying lower-limb motor control, neural dynamics, and the advancement of MI-based BCIs for rehabilitation and assistive technologies.

Key words: EEG dataset; lower-limb motor imagery; lower-limb motor execution; sit-to-stand transition; stand-to-sit transition; brain–computer interface (BCI); event-related desynchronization (ERD); movement-related cortical potential (MRCP)

Data Description

Background and Purpose

Motor imagery (MI), the mental simulation of movement without physical execution, is a central yet challenging paradigm in

Compiled on: November 9, 2025.
Draft manuscript prepared by the author.

Key Points

- First publicly available EEG dataset for sit-to-stand and stand-to-sit transitions.
- Multi-modal recordings (EEG, EOG, EMG) validated by EEGNet for MI and ME decoding.
- Provides benchmark framework and neurophysiological insights for lower-limb MI-BCI research.

electroencephalography (EEG)-based brain–computer interfaces (BCIs). MI-BCIs harness intentional brain activity to control external devices, such as assistive tools and computers, purely through thought. This paradigm shows strong potential in rehabilitation, neuroprosthetics, and assistive technologies, particularly for individuals with motor impairments, including stroke survivors and patients with neurodegenerative diseases [1]. Typically, MI-BCIs involve imagery of specific movements, such as hand or foot actions, that activate sensorimotor brain regions. These activations produce distinctive EEG patterns in the 8–30 Hz frequency range, characterized by event-related desynchronization (ERD)—a reduction in amplitude before or during the event—and event-related synchronization (ERS)—an increase in amplitude afterward. These rhythms form the foundation for decoding motor intentions for neurorehabilitation applications.

Beyond hand and foot movements, investigating the neural signals associated with sit-to-stand (sit–stand) and stand-to-sit (stand–sit) transitions provides valuable insights for developing mobility rehabilitation protocols and assistive devices. Recent studies have demonstrated the feasibility of utilizing EEG signals during sit–stand and stand–sit imagery to design MI-BCI systems that support lower-limb movement by accurately decoding motor intentions [2, 3, 4, 5, 6]. Building on these findings, MI-BCIs for this tasks hold promise for real-world applications, allowing smoother transitions between sitting and standing positions.

Despite its potential, sit-stand and stand-sit transitions remain underrepresented in MI-BCI research. Current publicly available MI-BCI EEG datasets predominantly focus on traditional hand and foot movements due to their well-defined and distinctive neural patterns [7, 8]. While these datasets have supported the development of advanced EEG-based classification algorithms, none offers EEG recordings of sit-stand or stand-sit motor imagery. This gap underscores the need for dedicated datasets focusing on these transitions to better capture lower-limb dynamics and expand the application of MI-BCIs in rehabilitation.

To advance research in this area, we present an EEG dataset comprising recordings from 22 participants during motor imagery (MI) and motor execution (ME) of sit-stand and stand-sit transitions, featuring up to 60 EEG channels. In addition to EEG, electrooculography (EOG) was collected to support artifact removal related to eye movements during standard preprocessing, and electromyography (EMG) was recorded concurrently to precisely determine movement onset during ME trials. As far as we know, this is the first publicly accessible EEG dataset explicitly targeting sit-stand and stand-sit transitions. It provides a valuable resource for researchers investigating lower-limb motor intentions reflected in EEG activity and developing EEG-based algorithms dedicated to lower-limb MI-BCIs. This contribution marks a significant step forward in advancing MI-BCI research and applications.

Experimental Design

Participants

Twenty-three healthy participants (aged 22–28 years; fifteen males) with no known neurophysiological abnormalities were recruited for this study. One participant (S05) was excluded due to poor signal quality, resulting in a final cohort of twenty-two participants. Prior to the experiment, research staff provided both verbal and written explanations of the study objectives, protocol, questionnaire, and experimental setup to ensure participants' comprehension. Written consent form was obtained from all participants in accordance with the Declaration of Helsinki. Participants received monetary compensation for their involvement. The experimental protocol and environment were reviewed and approved by the Ethics Committee of Suranaree University of Technology, Thailand (EC-65-0031). The demographic of subject and the questionnaire used in this study are provided in the supplementary material.

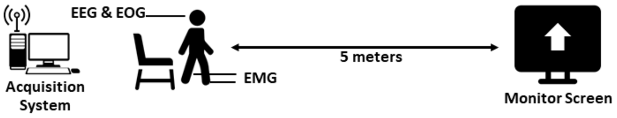

Figure 1. Experimental setup for data collection. A monitor displaying visual cues was placed in front of the participant, while the data acquisition system for synchronous EEG, EOG, and EMG recording operated by research staff was behind the participant.

Environment

All experiments were conducted in a quiet, controlled environment with only the participant and research staff present. The experimental setup is illustrated in Figure 1. For the sitting state, participants were seated approximately 5 meters from a 65-inch wall-mounted 4K LED monitor, oriented toward the monitor displaying visual cues. For the standing state, participants positioned themselves directly in front of the chair. The data acquisition system comprising a desktop computer running Windows 11, equipped with a 24-inch monitor, keyboard, and mouse, was placed on a table behind the participant. Research staff operated the system to synchronously record EEG, EOG, and EMG signals while ensuring the correct sequence of visual cues. To minimize fatigue effects, participants were instructed to rest adequately the night before, and all data collection sessions were scheduled during morning hours to ensure an optimal physiological state.

Data acquisition

Data Collection Protocol

Data acquisition was conducted in two identical sessions of motor tasks, separated by a 5–10 minute rest period. At the beginning of each session, baseline brain activity was recorded under two resting-state conditions: one minute with eyes closed (EC) followed by one minute with eyes open (EO). Participants then performed the ME task (40 trials per session), after which they completed the MI tasks. The MI tasks alternated between sitting and standing conditions and were repeated twice per session (20 trials per session). The overall procedure—including participant preparation, system setup, and data collection across both sessions—lasted approximately two hours per participant. The flow of the data collection protocol is visualized in Figure 2a.

Motor Execution (ME)
After collecting baseline EEG at the beginning of each session, participants performed the ME task, in which they carried out physical sit-stand (ME_SIT_STD) and stand-sit (ME_STD_SIT) movements. The task consisted of 40 trials, alternating between sit-to-stand and stand-to-sit transitions. For sit-stand trials, participants began seated on a chair, whereas for stand-sit trials, participants began standing in front of a chair. Each trial lasted 16 seconds, resulting in a total duration of 640 seconds (approximately 11 minutes).

The structure of each trial was as follows: a fixation cross appeared for 2 seconds to direct participants' gaze and signal trial preparation. A visual cue was then presented for 1 second: an upward arrow instructed participants to stand up from a seated position, while a downward arrow instructed them to sit down from a standing position. After the cue disappeared, participants executed the instructed movement within 5 seconds. Another fixation cross then appeared for 2 seconds, followed by a 1-second presentation of a white circle, instructing participants to rest (ME_R) in their current posture for the subsequent 5 seconds. This marked the completion of one trial.

Motor Imagery (MI)
Following the ME task, participants performed the MI task under two conditions: during sitting and during standing. In contrast to the previous task, participants were instructed to mentally simulate the sit-stand and stand-sit transitions without executing any physical movement.

Motor Imagery during Sit
In the MI during sit condition (Figure 2c), participants sat on a chair and observed sequential visual cues. Each 16-second trial began with a white cross for 2 seconds, prompting participants to focus and prepare for the upcoming cue. Subsequently, a 1-second visual cue appeared—a white up arrow indicating participants should imagine standing up from sitting (MI_SIT_STD) or a white down arrow indicating participants should imagine sitting down while already seated (MI_SIT_SIT). Participants imagined these movements for 5 seconds immediately after the cue disappeared. Another white cross appeared for 2 seconds, signaling preparation for the next cue, followed by a 1-second white circle instructing participants to rest. Participants then rested for 5 seconds (MI_R_SIT) while remaining seated to prevent fatigue.

MI during sit was divided into two nonconsecutive rounds, each consisting of 20 pseudorandomized trials (10 for MI_SIT_STD and 10 for MI_SIT_SIT). In the remaining part of this study, the data obtained during MI_SIT_SIT is not used. However, data are provided to support further exploration for potential study.

Motor Imagery during Stand
In the MI during stand condition (Figure 2d), participants stood in front of a chair while observing sequential visual cues. Each 16-second trial began with a white cross displayed for 2 seconds, indicating participants should attentively prepare for the upcoming instruction. Next, a 1-second visual cue appeared—a white down arrow instructing participants to imagine sitting down from standing (MI_STD_SIT) or a white up arrow instructing participants to imagine standing up while already standing (MI_STD_STD). Participants imagined these movements for 5 seconds immediately after cue disappearance. Another white cross appeared for 2 seconds, signaling preparation for the next cue, followed by a 1-second white circle instructing participants to rest. Participants then rested for 5 seconds (MI_R_STD) while remaining standing to avoid fatigue.

MI during stand was divided into two nonconsecutive rounds, each consisting of 20 pseudorandomized trials (10 for MI_STD_SIT and 10 for MI_STD_STD). In the remaining part of this study, the data obtained during MI_STD_STD is not used. However, data are provided to support further exploration for potential study.

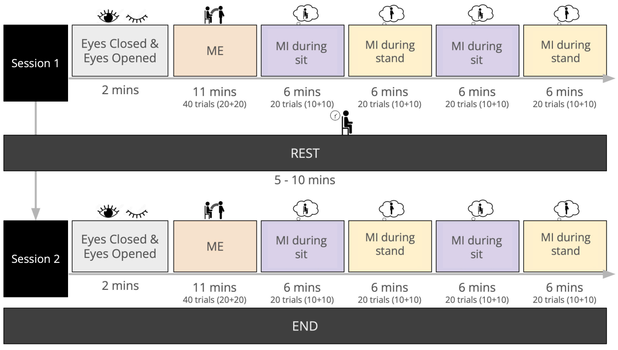

(a) Data Collection Protocol.

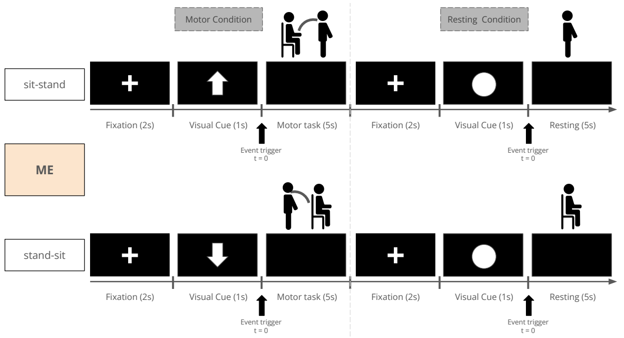

(b) Motor Execution (ME) activity. Each round alternated 20 sit-to-stand and 20 stand-to-sit trials, followed by a resting task.

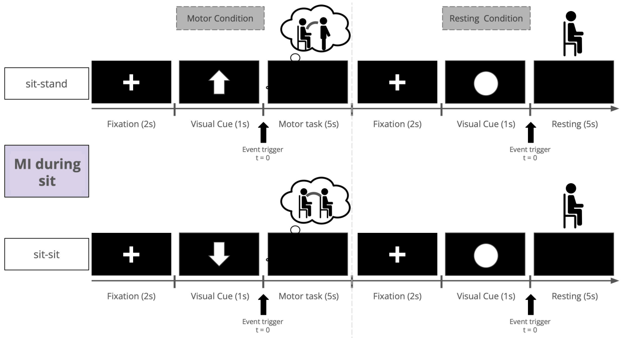

(c) Motor Imagery (MI) during sit. Each round included 10 sit-to-stand and 10 sit-to-sit trials in pseudorandom order, followed by a resting task.

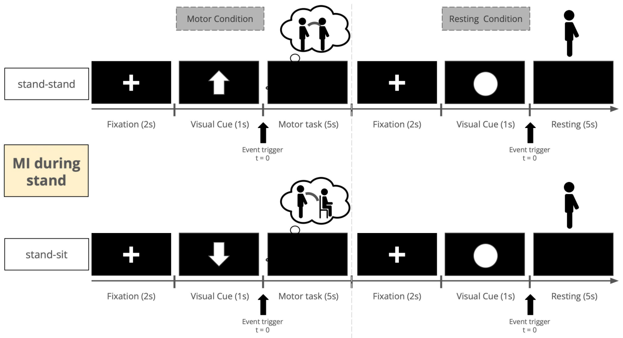

(d) Motor Imagery (MI) during stand. Each round included 10 stand-to-sit and 10 stand-to-stand trials in pseudorandom order, followed by a resting task.

Figure 2. Overview of the data collection process and task-related instructions are illustrated. Fig 2a overviews the whole process of data collection protocol sequentially. Fig 2b shows visual cue instructions in ME activity, while Fig 2c shows visual cue instructions in MI during sit, and Fig 2d shows visual cue instructions in MI during stand.

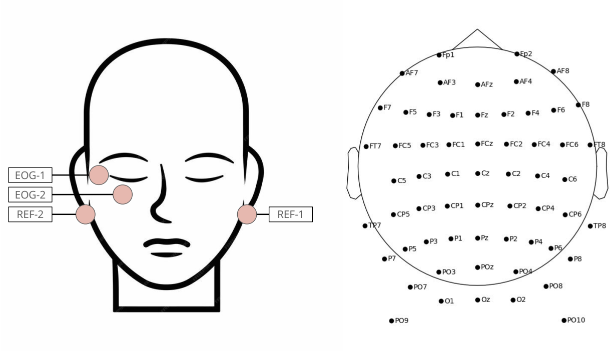

Figure 3. Electrodes placement positions for recording EEG and EOG signal.

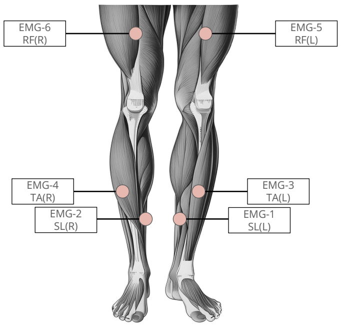

Figure 4. Surface EMG sensor placement positions for recording EMG signal.

EEG Signals

EEG recordings were acquired using a biosignal amplifier (g.HIamp, g.Tec, Austria) with a sampling rate of 1,200 Hz from 62 electrodes (60 EEG, 2 EOG) arranged according to the international 10–20 system. Of these, 60 electrodes recorded EEG signals, while two additional electrodes served as horizontal (hEOG) and vertical (vEOG) EOG signals, respectively. The 60 EEG channels recorded included Fp1, Fp2, AF7, AF8, F7, F8, FT7, FT8, AF3, AF4, AFz, Fz, F1, F2, F3, F4, F5, F6, FCz, Cz, FC1, FC2, FC3, FC4, FC5, FC6, C1, C2, C3, C4, C5, C6, CPz, Pz, CP1, CP2, CP3, CP4, CP5, CP6, TP7, TP8, P1, P2, P3, P4, P5, P6, P7, P8, POz, Oz, PO3, PO4, PO7, PO8, PO9, PO10, O1, and O2.

The FPz electrode served as the ground electrode. Signals from the left and right earlobes were averaged and applied to other channels as references. Throughout the experiment, electrode impedance was maintained below 30 $k\Omega$, and conductive gel was applied as needed. Electrode placement is depicted in Figure 3.

EOG Signals

Two electrodes were dedicated to recording electrooculography (EOG) signals to assist in ocular artifact removal from EEG data. One electrode was placed on the right temple (designated as EOG-1, or hEOG, using channel #61), while the other was positioned on the right infraorbital region (designated as EOG-2, or vEOG, using channel #62), as illustrated in Figure 3. In this study, EOG signals are used to assist in data preprocessing of EEG signals.

EMG Signals

Electromyography (EMG) signals were collected using six surface EMG sensors (Trigno Avanti Sensor, Delsys, USA) at a sampling rate of 2,000 Hz. Sensors were attached bilaterally to three lower-limb muscles: the Soleus (SL), Tibialis Anterior (TA), and Rectus Femoris (RF). The locations of EMG recording sensors are illustrated in Figure 4. In this study, EMG signals are used to assist in locating movement onset of ME activities.

Data Synchronization

All recorded physiological signals (EEG, EOG, and EMG) were synchronized with timestamps generated by the data acquisition system, operating on the Windows 11 operating system, prior to preprocessing and analysis.

Data format and structure

To enhance usability, both raw data in *.mat* format and preprocessed data in *.fif* format is provided. It is recommended to use the Python-based MNE library [9] for full access to *.fif* files. A detailed description of the datasets is presented in Table 1. This study focuses on electroencephalography (EEG) recordings collected while participants engaged in various brain-computer interface (BCI) motor-related activities, including motor execution (ME), motor imagery (MI) during sit, and MI during stand. Additionally, baseline EEG recordings were acquired during resting-state conditions, specifically during eyes-closed (EC) and eyes-opened (EO) states, prior to the execution of motor tasks. These baseline recordings are included to facilitate further analysis. According to Figure 2a, each experimental session consisted of a single run for ME, EC, and EO activities. In contrast, MI during sit and MI during stand activities were each performed twice per session. Consequently, two separate files were generated for each session of MI during sit, and MI during stand, labeled as *_S1* and *_S2*. The raw EEG data for each participant are structured as a matrix of dimensions *n_channels* × *n_timepoints*. An extra channel (channel #63) is allocated for event triggers, which annotate instruction-related events serving as ground truth labels. The processed EEG data are structured as a three-dimensional matrix of size *n_trials* × *n_channels* × *n_timepoints*. A detailed description of event trigger codes is provided in Table 2.

Data Validation

Data Preprocessing

This study's EEG recordings were preprocessed to analyze brain activity during EC, EO, MI, and ME tasks. Specifically, movement-related cortical potential (MRCP) features were examined, predominantly associated with ME activities. Conversely, time-frequency power distribution features were analyzed for EC, EO, and MI activities, as recommended and validated in previous studies [4, 10]. Due to these differences, the preprocessing pipelines for these activities varied slightly, as described in detail below. All preprocessing steps were conducted using the MNE-Python library [9].

Preprocessing Steps for Movement-Related Cortical Potential (MRCP) Analysis

For MRCP feature extraction in ME-based classification, the following preprocessing steps were applied:

The 60-channel EEG recordings were first filtered with a second-order Butterworth bandpass filter with a 0.2 to 3 Hz cutoff frequencies. Subsequently, the signals were downsampled to 250 Hz. To remove artifacts, independent component analysis (ICA) was performed to decompose independent components (ICs) and eliminate muscle- and eye-related artifacts, using recorded EMG and EOG signals to assist the ICA process. Following, signals from identified bad channels were removed and interpolated using data from neighboring electrodes. To mitigate volume conduction effects and enhance spatial resolution, the Current Source Density (CSD) trans-

Name	Description
**Raw Data**	
S<*ID*>_S<$session_{num}$>.mat	Raw signal recorded from subject *ID* in session $session_{num}$
S01_S1.mat	*Example:* Raw signal recorded from subject 01 in session 1
**Processed Data**	
S<*ID*>.fif	Pre-processed signal from subject *ID*.
S01.fif	Pre-processed signal from subject 01.

Table 1. Data description table.

Event	Description
1	Eyes closed resting state.
2	Eyes opened resting state.
10	Start of trials in ME activity.
11	Start of ME_SIT_STD in ME activity.
12	Start of ME_STD_SIT in ME activity.
13	Start of resting condition (ME_R) in ME activity.
20	Start of trials in MI during sit.
21	Start of MI_SIT_STD in MI during sit.
22	Start of MI_SIT_SIT task in MI during sit.
23	Start of resting condition in MI during sit (MI_R_SIT, rest while sitting).
30	Start of trials in MI during stand.
31	Start of MI_STD_STD task in MI during stand.
32	Start of MI_STD_SIT in MI during stand.
33	Start of resting condition in MI during stand (MI_R_STD, rest while standing).

Table 2. Event trigger number description table. The event trigger is stored at EEG channel number #63 in the .mat file and embedded into MNE Epochs from the .fif file.

formation was applied [11]. Finally, the preprocessed signals were segmented into 4-second epochs ranging from -2 to 2 seconds relative to an onset of EMG trigger event (indicating $T = 0$). (Note: The EMG data from session #2 of subject #20 is unavailable. To resolve this issue, specifically for this circumstance, the 4-second epochs were segmented using an onset of the event triggers #11 and #12 obtained from channel number #63.) Furthermore, we excluded those trials contaminated by noise and amplitude spikes with a trial rejection based on peak-to-peak (PTP) amplitude calculation of EEG signals, as those trials with large PTP amplitudes indicate the presence of artifacts. Any trials with the PTP amplitudes exceed this 95th percentile threshold is automatically flagged and rejected from further analysis. On average, 36 trials are remained after the rejection. Thus, the first 36 trials selected for further analysis.

For ME-related classification, each trial was labeled according to a participant's physical movement either performing ME task or ME resting (ME_R). The ME resting trials are alternately separated into either sit-rest (ME_R_SIT) or stand-rest (ME_R_STD) such that, start from index 0, the even-numbered trials are labeled as ME_R_STD, while the odd-numbered trials are labeled as ME_R_SIT. ME_R_SIT and ME_R_STD are corresponded to a participant resting while sitting and standing, respectively. Similarly, trials labeled as ME_SIT_STD and ME_STD_SIT are corresponded to a participant performing a sit-to-stand and stand-to-sit transitions, respectively.

Preprocessing Steps for Time-Frequency Distribution Analysis

For MI, EC, and EO activities, as well as MI-related classification, preprocessing steps were similar to those used for MRCP analysis, with the following modifications:

Instead of using a second-order Butterworth bandpass filter (0.2–3 Hz), a sixth-order Butterworth bandpass filter with 1–40 Hz cutoff frequencies was applied. The processed signals were also segmented into trials ranging from -2 to 5 seconds relative to the event trigger onset.

In contrast to the ME task, the MI task did not involve any actual movement; therefore, no EMG onset was observed. The preprocessed signals were segmented into epochs relative to the onset of the event trigger #21 and #32, as described in Table 2. Each trial spanned 7 seconds (2 seconds before and 5 seconds after the event trigger onset). In order to minimize influence of ongoing background activity, a 2-second segment before the event trigger was used for baseline correction, leaving a 5-second segment for further analysis. We excluded those trials contaminated with noise by a threshold-based trial rejection using PTP amplitude calculation, similar to the steps for MRCP analysis. On average, 36 trials are remained after the rejection and are selected for further analysis. For MI-related classification, trials were labeled according to a participant's current physical states. MI_R_SIT and MI_R_STD corresponded to a participant resting while sitting and standing, respectively. Similarly, trials labeled as MI_SIT_STD and MI_STD_SIT corresponded to a participant imagining of a sit-to-stand and stand-to-sit transitions, respectively.

It should be noted that the EEG data preprocessed with ICA and CSD were utilized for visualization, whereas data without these preprocessing steps were used for classification with EEGNet.

Method

Classification Techniques and Models

To validate the correctness of the proposed dataset, we conduct the classification experiments using EEGNet[12] model, the commonly used state-of-the-art approaches in BCI classification studies, to classify EEG recordings from ME and MI activities. EEG signals exhibit high inter-subject variability, making subject-independent classification crucial for broader BCI applications [13]. We employ leave-one-subject-out cross-validation (LOSOCV) to ensure test data remain unseen during training. Each subject's EEG recordings are treated as independent samples, enhancing robustness against inter-subject variability. The data input shape to train and validate the model is $n_{subject} - 1 \times n_{trial} \times n_{channel} \times n_{timept}$, while the data input shape to test the model is $1 \times n_{trial} \times n_{channel} \times n_{timepts}$, where $n_{subject}$ represents total number of subjects, n_{trial} represents total number of trials that each subject performed, $n_{channel}$ represents total number of EEG channels, $n_{timepts}$ represents length of EEG samples.

EEGNet-8,2: EEGNet [12] effectively learns spatiotemporal EEG features through a compact convolutional neural network. It employs depthwise and separable convolutions to enhance feature learning while reducing trainable parameters, improving efficiency without sacrificing classification performance in EEG-based BCI tasks. Our implementation uses Python with PyTorch [14] and scikit-learn [15]. The parameters settings of EEGNet-8,2 are set as follows: number of filters in the first layer $F1 = 8$, depth parameter $D = 2$, kernel size $C1 = 200$, number of classes $n_{class} = 2$, dropout rate $dropout = 0.5$. The optimal batch size b and learning rate lr are set to be $b = 8$ and $lr = 1 \times 10^{-3}$, respectively. The model is trained subjected to cross-entropy loss for 200 epochs, while the early stopping is triggered when the loss does not decrease for 10 consecutive epochs. To ensure unbiased learning, we apply five-fold cross-validation when training and validating EEGNet for an optimal model's performance.

Table 3. Classification performance of EEGNet, along with training T_{train} and inference T_{infer} times (in seconds) per one fold, on the proposed dataset for motor execution (ME) and motor imagery (MI) tasks using different EEG segment lengths (Mean ± SD).

Experiment	Task	Segment Length (s)	Accuracy ↑	F1-score ↑	AUC ↑	T_{train} (s) ↓	T_{infer} (s) ↓
ME	ME_SIT_STD vs ME_R_SIT	1	75.91 ± 9.38*	70.87 ± 14.84*	0.8210 ± 0.1091*	70.01 ± 9.33*	0.06 ± 0.01
		2	**79.85 ± 7.99**	**77.60 ± 10.47**	**0.8844 ± 0.0751**	**63.37 ± 7.63**	**0.06 ± 0.01**
	ME_STD_SIT vs ME_R_STD	1	76.02 ± 9.68*	71.68 ± 15.62*	0.8302 ± 0.0984*	**61.40 ± 5.53**	0.06 ± 0.01
		2	**79.68 ± 8.80**	**77.93 ± 11.88**	**0.8866 ± 0.0836**	63.22 ± 6.65	**0.06 ± 0.01**
MI	MI_SIT_STD vs MI_R_SIT	1	69.16 ± 8.62	67.48 ± 13.28	0.7677 ± 0.0981	**86.96 ± 12.95**	0.08 ± 0.01
		2	**69.58 ± 8.86**	69.69 ± 11.15	0.7994 ± 0.0973	92.32 ± 15.79	**0.07 ± 0.01**
		3	68.98 ± 9.56	**70.70 ± 11.84**	**0.8045 ± 0.0975**	155.97 ± 27.96*	0.14 ± 0.03*
		4	67.85 ± 10.24	69.58 ± 12.91	0.7992 ± 0.1016	168.59 ± 32.24*	0.13 ± 0.04*
		5	67.39 ± 10.25	69.16 ± 14.02	0.7985 ± 0.1031	167.96 ± 25.68*	0.14 ± 0.03*
	MI_STD_SIT vs MI_R_STD	1	71.37 ± 7.44	68.73 ± 13.24	0.7872 ± 0.0969	**83.03 ± 11.12**	0.07 ± 0.01
		2	71.27 ± 8.33	**70.15 ± 13.16**	0.8022 ± 0.1001	95.65 ± 13.74*	**0.07 ± 0.01**
		3	70.88 ± 10.82	68.77 ± 19.01	0.8080 ± 0.1204	124.96 ± 24.34*	0.08 ± 0.03
		4	**71.42 ± 11.13**	68.58 ± 20.98	**0.8149 ± 0.1129**	149.74 ± 19.01*	0.07 ± 0.01
		5	70.63 ± 11.54	67.51 ± 21.92	0.8049 ± 0.1215	148.15 ± 14.33*	0.07 ± 0.02

Note: * indicates statistically significant difference using a *t*-test ($p \leq 0.05$) compared to the best-performing setting, highlighted in bold. Segment length refers to the EEG window duration in seconds, extracted relative to stimulus onset (before onset for ME, after onset for MI).
Abbreviations: ME_SIT_STD, executing standing up from sitting; ME_R_SIT, rest while sitting; ME_STD_SIT, executing sitting down from standing; ME_R_STD, rest while standing; MI_SIT_STD, imagining standing up from sitting; MI_R_SIT, rest while sitting; MI_STD_SIT, imagining sitting down from standing; MI_R_STD, rest while standing; T_{train}, training time; T_{test}, inference time.

Experiments

This study presents BCI classification in two experimental scenarios – motor execution (ME) and motor imagery (MI) – as the analysis approaches are varied.

Experimental Design for Experiment 1: the MRCP analysis for ME-based classification

For ME-activity classification, this study focuses on analyzing and decoding subject's movement intention from Movement-Related Cortical Potential (MRCP), a low frequency (less than 3 Hz) cortical potential with negative shift in EEG signal which is observed before the onset of real movement and being used to detect motor intention, with a direct association to primary motor and somatosensory cortices [16, 17]. As the MRCP is the premovement cortical potential, it usually happens within two seconds prior the movement onset. In this study, we analyze the EEG time-series in this interval to train, validate, and test the classifier.

In the ME classification experiment, we concentrate on classifying ME activities during transition and resting stages using premovement signals. Specifically, we classify EEG recordings in two tasks – (1) movement of standing up from sitting (ME_SIT_STD) vs. rest during sitting (ME_R_SIT) and (2) movement of sitting down from standing (ME_STD_SIT) vs. rest during standing (ME_R_STD). In addition, to study the optimal segment length of EEG for usage in further applications, we trained and tested the classifier on two distinct EEG time windows – one second before movement and two seconds before movement. These time intervals were chosen to capture pre-movement brain activity and evaluate its predictive value for classification. The data input shape for this binary-classification experiment for 2-second data is 22 *subjects* × 72 *trials* × 60 *channels* × 500 *timepts*.

Experimental Design for Experiment 2: the time-frequency analysis for MI-based classification

For MI-activity classification, this study focuses on analyzing and decoding subject's movement imagination from time-frequency distribution of EEG time-series in range 1 to 40 Hz. Similar to ME, we classify EEG recordings in two tasks – (1) movement imagination of standing up from sitting (MI_SIT_STD) vs. rest during sitting (MI_R_SIT) and (2) movement imagination of sitting down from standing (MI_STD_SIT) vs. rest during standing (MI_R_STD). Furthermore, to study the optimal segment length of EEG for usage in further applications, we trained and tested the classifier on five distinct EEG time windows – one second, two seconds, three seconds, four seconds, and five seconds after the onset of stimulus. These time intervals were chosen to capture brain activity and evaluate its predictive value for classification. The data input shape for this binary-classification experiment for 5-second data is 22 *subjects* × 72 *trials* × 60 *channels* × 1250 *timepts*.

Performance Matrix and Evaluation

In this study, classification performance is assessed and reported in terms of accuracy (ACC) to measure the correct classification rate, F1-score (F1) to assess the balance between precision and recall, and area under the curve (AUC) that provides useful insights on the model's ability to handle robustness of class-imbalance classification.

Qualitative Analysis

In addition to the reported classification performance, to analyze activation of EEG spatially in response to the onset of the visual stimulus, we have visualized topographical map on transition against resting conditions in MI activity on different tasks, on different frequency rhythms, as presented in the upcoming section. These visualizations provide readers additional insights on how state of the brain changes at different time intervals, in comparison to the baseline. Additionally, to select suitable interval for motor classification, experiments on variations of EEG segment length provide useful insights to assist readers for further studies and analysis.

Results and Discussion

Performance Analysis

This section presents classification performance for both ME and MI activities. For ME activity, we focused on classifying EEG signals obtained from two tasks – ME_SIT_STD vs. ME_R_SIT (classifying EEG signals between motor execution during sit-to-stand transition and rest sitting) and ME_STD_SIT vs. ME_R_STD (classifying EEG signals between motor execution during stand-to-sit transition and rest standing). In order to assess the robustness of the models, we also conducted experiments to evaluate the models' performance on the variations of EEG segment length. Experiments in this study are carried out by using EEGNet, the state of the art deep

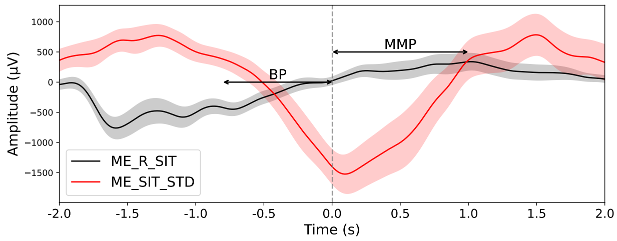

(a) During sit task in ME experiment; ME_SIT_STD vs. ME_R_SIT.

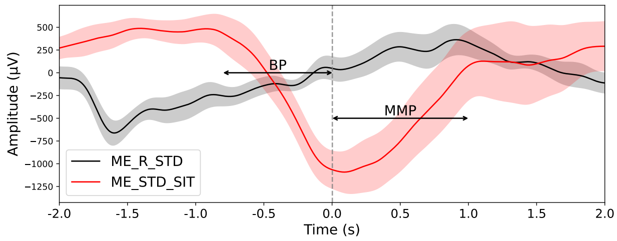

(b) During stand task in ME experiment; ME_STD_SIT vs. ME_R_STD).

Figure 5. Grand-average across all subjects with standard error of nine electrodes around motor cortex area from EEG activity during ME experiment.

learning model in BCI studies.

We observe the model's performance, in terms of both classification results and running time, from Table 3 that, in ME_SIT_STD vs. ME_R_SIT task, EEGNet has attained the best accuracy of 79.85 ± 7.99%, 77.60 ± 10.47% in terms of F1-score, and 0.8844 ± 0.0751 in terms of AUC, on 2-second segment, with the average training time 63.37 ± 7.63 seconds per one fold and average inference time 0.06 ± 0.01 second. In ME_STD_SIT vs. ME_R_STD task, EEGNet also has attained the best accuracy of 79.68 ± 8.80%, 77.93 ± 11.88% in terms of F1-score, and 0.8866 ± 0.0836 in terms of AUC, on 2-second segment, with the average training time 61.40 ± 5.53 seconds per one fold and average inference time 0.06 ± 0.01 second.

Similarly, for MI activity we classified EEG signals from two tasks: MI_SIT_STD vs. MI_R_SIT (motor imagery of sit-to-stand vs. rest while sitting) and MI_STD_SIT vs. MI_R_STD (motor imagery of stand-to-sit vs. rest while standing). To assess robustness, we evaluated performance across varying EEG segment lengths using the EEGNet model. From Table 3, for MI_SIT_STD vs. MI_R_SIT, EEGNet achieved its best accuracy of 69.58 ± 8.86% with 2-second segments, while the best F1-score and AUC—70.70 ± 11.84% and 0.8045 ± 0.0975, respectively—were obtained with 3-second segments. The most optimal training and inference times were 86.96 ± 12.95 seconds and 0.07 ± 0.01 seconds per segment, respectively. For MI_STD_SIT vs. MI_R_STD, EEGNet achieved the best accuracy of 71.42 ± 11.13% and AUC of 0.8149 ± 0.1129 with 4-second segments, F1-score of 70.15 ± 13.16% with 2-second segment, with training and inference times of 83.03 ± 11.12 seconds and 0.07 ± 0.01 seconds, respectively.

Qualitative Analysis

This study also performed qualitative analyses of the recorded EEG signals to confirm the correctness and enhance explainability of the obtained data. In the ME task, we focus on analyzing the EEG signals in terms of movement-related cortical potentials (MRCPs), spontaneous potentials generated during person-generated movement [4, 18]. Figure 5 shows grand average of EEG signals during ME activity on both during sit and stand tasks across all subjects. The visualizations are obtained from 9 electrodes around motor cortex area, including FC1, FCz, FC2, C1, Cz, C2, CP1, CPz, CP2. During the sit task (sit-to-stand, or ME_SIT_STD), it is observed from Figure 5a that, comparing to rest sitting (ME_R_SIT), Bereitschaftspotential (BP, also known as readiness potential) exhibits slow negative EEG about 0.8 second (-0.8 to 0 s) before the actual onset of movement (designated by dotted gray vertical line) where the peak negativity lies around 0 s. Movement-monitoring potential (MMP), a component of MRCPs that reflects brain activity after executing a voluntary movement relates the brain's monitoring of the physical movement precision and control, lasts for about 1 second after the physical movement onset.

On the other hand, for the stand task (stand-to-sit, or ME_STD_SIT), it is observed from Figure 5b that, comparing to the rest standing (ME_R_STD), BP potential starts around 0.8 second (-0.8 s) before the actual onset of movement, gradually decreasing to the peak negativity around 0.25 second. The MMP lasts for about 1 second after the physical movement onset.

A small latency in negative peak of MRCP signals is observed in the stand-to-sit (or ME_STD_SIT) task, compared to the sit-to-stand (or ME_SIT_STD) task. This could be related to the asymmetry of neural preparatory process between these two tasks. The ME_SIT_STD task is considered to be a propulsive movement, requiring a rapid generation of force against gravity and inertia. This task demands a strong and immediate motor output which may requires a more decisive and rapid cortical preparation. Conversely, the ME_STD_SIT task involves a controlled descent, which requires precise and continuous modulation of muscle activity to decelerate the body and ensure a smooth, stable sitting. This control might involve a more prolonged preparatory phase, potentially delaying the MRCP negative peak [18].

For the MI task, we analyze EEG signals in the time–frequency domain, i.e., how spectral components evolve over time. Figure 6 presents topographical maps of spectral power in the δ (1-4 Hz), θ (4-8 Hz), α (8-13 Hz), and β (13-30 Hz) bands, visualized for each second across a 5-second window. In terms of ERD/ERS, we observe pronounced event-related desynchronization (ERD)—a decrease in the power of an EEG rhythm—in frontal and central regions during the transition periods of both sit and stand motor-imagery tasks. This ERD is evident in the δ, θ, and α bands and is particularly strong in the β band, consistent with increased cortical activation and sensorimotor information processing during movement-related transitions.

On the other hand, we observe strong event-related synchronization (ERS)—an increase in the power of an EEG rhythm—primarily in the parietal region during resting (non-movement) periods, especially in the β band. In the δ, θ, and α bands, pronounced ERS is also evident in the parietal and frontal regions during resting. These observations are consistent with the findings reported by [19].

Discussion

This study presents and evaluates the performance of EEG-based dataset, focusing on neural activation pattern in motor imagery and execution during sitting and standing tasks. As EEG has high variance across individuals [13], to support variety of future applications on motor tasks, we train, validate, and test on cross-subject basis.

In the ME task, we present the classification performance for EEG signals obtained during the transition periods and resting. Since this classification task focuses on training the model to learn MRCP features, we compare the model's performance, taking 1-second and 2-second EEG segment prior the movement onset for training and testing. From the results shown in Table 3, we see that the classification results from the EEGNet model on 2-second EEG gives the best performance in terms of classification performance and training time. Although 1-second EEG segment before the movement onset could give the best performance for at least 75.91% on accuracy, using 2-second EEG segment provides significantly improved performance for at least 79.68%, with around 63 seconds training time. This is related to an ability of classifier to learn a longer window of BP components. Compared to the resting EEG segments, we observe from Figure 5 that longer segments

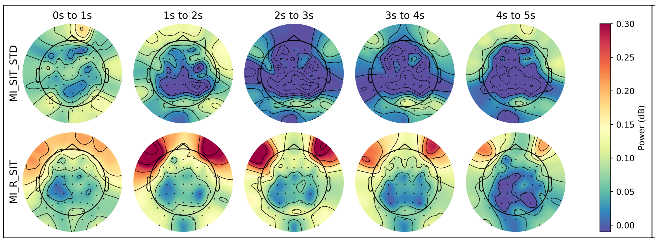

(a) MI_SIT_STD vs MI_R_SIT in δ rhythm.

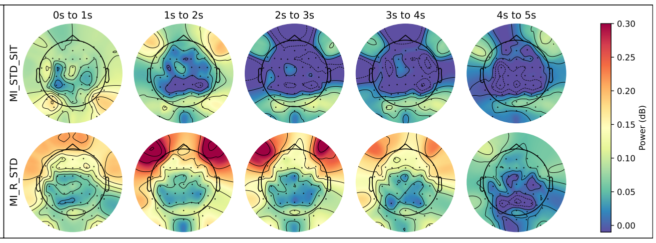

(b) MI_STD_SIT vs MI_R_STD in δ rhythm.

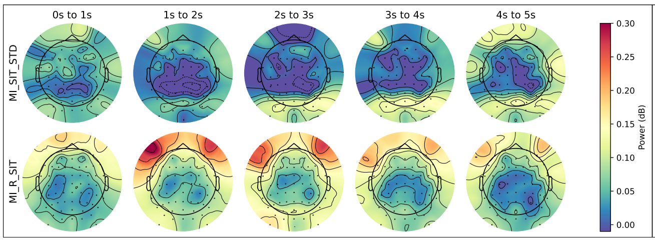

(c) MI_SIT_STD vs MI_R_SIT in θ rhythm.

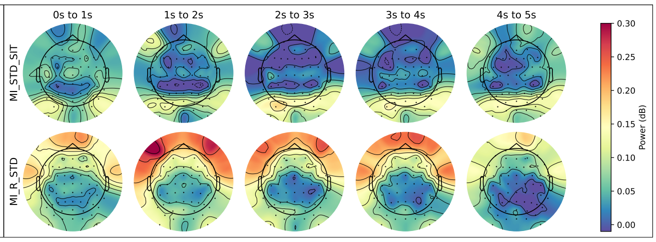

(d) MI_STD_SIT vs MI_R_STD in θ rhythm.

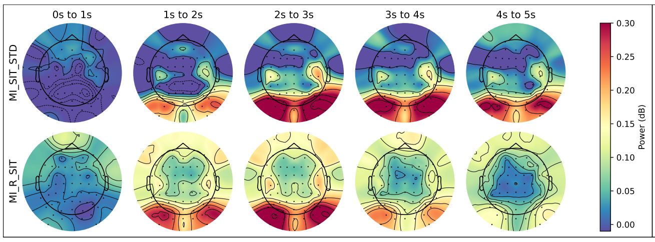

(e) MI_SIT_STD vs MI_R_SIT in α rhythm.

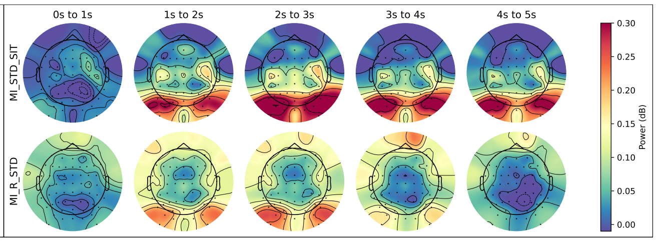

(f) MI_STD_SIT vs MI_R_STD in α rhythm.

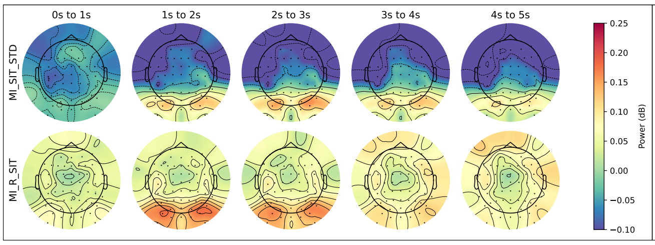

(g) MI_SIT_STD vs MI_R_SIT in β rhythm.

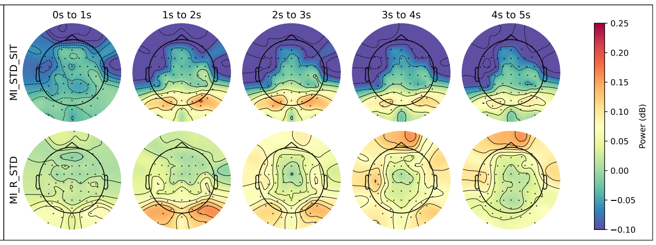

(h) MI_STD_SIT vs MI_R_STD in β rhythm.

Figure 6. Topographical map visualizations for grand average of spectral power in δ (1-4 Hz), θ (4-8 Hz), α (8-13 Hz), and β (13-30 Hz) rhythms, calculated from the Morlet wavelet across 5 seconds window after the visual stimulus onset during various motor imagery (MI) activities. This includes MI_SIT_STD (motor imagery during sit to stand, or standing up from sitting on a chair) vs MI_R_SIT (resting while sitting on a chair) and MI_STD_SIT (motor imagery during stand to sit, or sitting on a chair from standing up) vs MI_R_STD (resting while standing).

clearly show the BP components of ME activity related to the cortical excitability and readiness for the movement [20, 21].

In the MI task, we present the classification performance for EEG signals obtained during the transition periods and resting. This classification task focuses on training the model to learn time-frequency features, we compare the model's performance when taking 1-second, 2-second, 3-second, 4-second, and 5-second EEG segment after the visual stimulus onset for training and testing. From the results shown in Table 3, we see that the classification results from the EEGNet model on 2-second EEG generally gives the optimal performance in terms of classification performance and training time. For MI_SIT_STD vs. MI_R_SIT (sit-to-stand motor imagery vs sit-rest), using 2-second EEG segment after the stimulus onset generally gives the optimal performance for 69.58% on accuracy and 92.32 seconds on training time. For MI_STD_SIT vs. MI_R_STD (stand-to-sit motor imagery vs stand-rest) tasks, although the best performance can be obtained using 4-second EEG segment, considering a balance trade-offs on the segment length, computation time, and a little performance gain, using the 2-second MI EEG segment seemed to be a good option. The reported performance are 71.42% on accuracy and 95.65 seconds on the training time.

To support this, Figure 6 shows topographical map visualizations for grand average of spectral power in δ, θ, α, and β rhythms on 5-second of MI EEG intervals. We observed from the figure that, for both MI_SIT_STD vs. MI_R_SIT and MI_STD_SIT vs. MI_R_STD conditions, the differences of EEG distributions can be observed from 2 seconds onward. These visualizations correspond to the classification results previously reported in Table 3, ensuring the optimal selection of EEG segment lengths.

Conclusion

This study introduces a novel EEG dataset focused on sitting and standing transitions during both motor execution (ME) and motor imagery (MI) tasks. The data, including 60 EEG channels, EOG, and EMG, obtained from 22 participants conducted two data recording

sessions, focusing on lower-limb movements. To ensure the usability of the proposed dataset, we have evaluated its performance on EEGNet, a state-of-the-art BCI classification algorithm on two tasks – sit-to-stand vs. sit-rest and stand-to-sit vs. stand-rest. In addition, we also evaluate the performance on various EEG segment lengths to suggest further BCI applications. This dataset lays a solid foundation for the development and benchmarking of future BCI algorithms, offering researchers a valuable resource to better understand and enhance the performance of MI-based BCIs.

Abbreviations

EC: eyes-closed resting state; EO: eyes-opened resting state; ERD: event-related desynchronization; ERS: event-related synchronization; ERSP: event-related spectral perturbation; ME: motor execution; ME_R: resting during motor execution; ME_R_SIT: sit-resting during motor execution; ME_R_STD: stand-resting during motor execution; ME_SIT_STD: executing standing up from sitting; ME_STD_SIT: executing sitting down from standing; MI: motor imagery; MI_R: resting during motor imagery; MI_R_SIT: sit-resting during motor imagery; MI_R_STD: stand-resting during motor imagery; MI_SIT_STD: imagining standing up from sitting; MI_SIT_SIT: imagining sitting while sitting; MI_STD_SIT: imagining sitting down from standing; MI_STD_STD: imagining standing while standing; MRCP: movement-related cortical potential

Acknowledgment

We acknowledge the following funding and collaboration support from PTT Public Company Limited, The SCB Public Company Limited, Suranaree University of Technology (SUT), Thailand Science Research and Innovation (TSRI), and National Science, Research and Innovation Fund (NSRF), Thailand. This research has received funding support from the National Science, Research and Innovation Fund (NSRF) via the Program Management Unit for Human Resources & Institutional Development, Research and Innovation [grant number B13F680099].

Funding

National Science, Research and Innovation Fund (NSRF) Thailand, NRIIS Number: 179275, G. Bhakdisongkhram. National Science, Research and Innovation Fund (NSRF) via the Program Management Unit for Human Resources & Institutional Development, Research and Innovation Thailand, Grant Number: B13F680099, T. Wilaiprasitporn.

Data Availability Statement

The data set supporting the results of this article is available in the Zenodo repository (doi:10.5281/zenodo.17561969).

References

1. Arpaia P, Esposito A, Natalizio A, Parvis M. How to successfully classify EEG in motor imagery BCI: a metrological analysis of the state of the art. Journal of Neural Engineering 2022 jun;19(3):031002.
2. Bulea TC, Prasad S, Kilicarslan A, Contreras-Vidal JL. Sitting and standing intention can be decoded from scalp EEG recorded prior to movement execution. Frontiers in Neuroscience 2014;8.
3. Jeong JH, Kwak NS, Guan C, Lee SW. Decoding Movement-Related Cortical Potentials Based on Subject-Dependent and Section-Wise Spectral Filtering. IEEE Transactions on Neural Systems and Rehabilitation Engineering 2020;28(3):687–698.
4. Chaisaen R, Autthasan P, Mingchinda N, Leelaarporn P, Kunaseth N, Tammajarung S, et al. Decoding EEG Rhythms During Action Observation, Motor Imagery, and Execution for Standing and Sitting. IEEE Sensors Journal 2020;20(22):13776–13786.
5. Triana-Guzman N, Orjuela-Cañon AD, Jutinico AL, Mendoza-Montoya O, Antelis JM. Decoding EEG rhythms offline and online during motor imagery for standing and sitting based on a brain-computer interface. Frontiers in Neuroinformatics 2022 Sep;16.
6. Singh B, Natsume K. Readiness potential reflects the intention of sit-to-stand movement. Cognitive Neurodynamics 2023;17(3):605–620.
7. Autthasan P, Chaisaen R, Phan H, Vos MD, Wilaiprasitporn T. MixNet: Joining Force of Classical and Modern Approaches Toward the Comprehensive Pipeline in Motor Imagery EEG Classification. IEEE Internet of Things Journal 2024;11(17):28539–28554.
8. Autthasan P, Chaisaen R, Sudhawiyangkul T, Rangpong P, Kiatthaveephong S, Dilokthanakul N, et al. MIN2Net: End-to-End Multi-Task Learning for Subject-Independent Motor Imagery EEG Classification. IEEE Transactions on Biomedical Engineering 2022;69(6):2105–2118.
9. Gramfort A, Luessi M, Larson E, Engemann DA, Strohmeier D, Brodbeck C, et al. MEG and EEG Data Analysis with MNE-Python. Frontiers in Neuroscience 2013;7(267):1–13.
10. Kongwudhikunakorn S, Kiatthaveephong S, Thanontip K, Leelaarporn P, Piriyajitakonkij M, Charoenpattarawut T, et al. A Pilot Study on Visually Stimulated Cognitive Tasks for EEG-Based Dementia Recognition. IEEE Transactions on Instrumentation and Measurement 2021;70:1–10.
11. Rathee D, Raza H, Prasad G, Cecotti H. Current Source Density Estimation Enhances the Performance of Motor-Imagery-Related Brain–Computer Interface. IEEE Transactions on Neural Systems and Rehabilitation Engineering 2017;25(12):2461–2471.
12. Lawhern VJ, Solon AJ, Waytowich NR, Gordon SM, Hung CP, Lance BJ. EEGNet: a compact convolutional neural network for EEG-based brain–computer interfaces. Journal of neural engineering 2018;15(5):056013.
13. Kongwudhikunakorn S, Ponwitayarat W, Kiatthaveephong S, Polpakdee W, Yagi T, Senanarong V, et al. EEGMeNet: End-to-End Multitask Neural Network for Brain-Based Mental Workload Classification. IEEE Internet of Things Journal 2025;12(20):42573–42589.
14. Musgrave K, Belongie SJ, Lim SN. PyTorch Metric Learning. ArXiv 2020;abs/2008.09164.
15. Pedregosa F, Varoquaux G, Gramfort A, Michel V, Thirion B, Grisel O, et al. Scikit-learn: Machine Learning in Python. Journal of Machine Learning Research 2011;12(85):2825–2830.
16. Karimi F, Kofman J, Mrachacz-Kersting N, Farina D, Jiang N. Detection of Movement Related Cortical Potentials from EEG Using Constrained ICA for Brain-Computer Interface Applications. Frontiers in Neuroscience 2017 Jun;11.
17. Li C, Guan H, Huang Z, Chen W, Li J, Zhang S. Improving Movement-Related Cortical Potential Detection at the EEG Source Domain. In: 2021 10th International IEEE/EMBS Conference on Neural Engineering (NER); 2021. p. 214–217.
18. Olsen S, Alder G, Williams M, Chambers S, Jochumsen M, Signal N, et al. Electroencephalographic Recording of the Movement-Related Cortical Potential in Ecologically Valid Movements: A Scoping Review. Frontiers in Neuroscience 2021 Sep;15.
19. Jeon Y, Nam CS, Kim YJ, Whang MC. Event-related (De)synchronization (ERD/ERS) during motor imagery tasks: Implications for brain–computer interfaces. International Journal of Industrial Ergonomics 2011 Sep;41(5):428–436.

20. Di Russo F, Berchicci M, Bozzacchi C, Perri RL, Pitzalis S, Spinelli D. Beyond the "Bereitschaftspotential": Action preparation behind cognitive functions. Neuroscience and Biobehavioral Reviews 2017 Jul;78:57–81.
21. Ravi A, Wolfe P, Tung J, Jiang N. Signal Characteristics, Motor Cortex Engagement, and Classification Performance of Combined Action Observation, Motor Imagery and SSMVEP (CAMS) BCI. IEEE Transactions on Neural Systems and Rehabilitation Engineering 2025;33:1004–1013.
