# Supplementary material for: EEG-based dataset explicitly targets the transitions between sitting and standing for exploring neural activation patterns in motor imagery and execution
Source: Gigascience. 2026 May 29;15:giag065. doi: 10.1093/gigascience/giag065 (PMC13270978; doi:10.1093/gigascience/giag065)
Supplement: giag065_GIGA-D-25-00472_revision_1 [file giag065_giga-d-25-00472_revision_1.pdf]

GigaScience

EEG-Based Dataset Explicitly Targeting the Transitions between Sitting and Standing for Exploring Neural Activation Patterns in Motor Imagery and Execution

--Manuscript Draft--

**Manuscript Number:**	GIGA-D-25-00472R1
**Full Title:**	EEG-Based Dataset Explicitly Targeting the Transitions between Sitting and Standing for Exploring Neural Activation Patterns in Motor Imagery and Execution
**Article Type:**	Data Note
**Funding Information:**	National Science Research and Innovation Fund (NSRF), the Program Management Unit for Human Resources & Institutional Development Research and Innovation Thailand (B13F680099) — Prof. Theerawit Wilaiprasitporn
	National Science Research and Innovation Fund (NSRF) Thailand (NRIIS Number: 179275) — Prof. Gun Bhakdisongkhram
	Thailand Science Research and Innovation (FRB690039/0457) — Prof. Theerawit Wilaiprasitporn
**Abstract:**	This study presents the first publicly accessible electroencephalography (EEG) dataset explicitly targeting sit-to-stand and stand-to-sit transitions during both motor execution (ME) and motor imagery (MI) tasks. Twenty-two healthy participants performed sitting and standing transitions under well-controlled experimental conditions while 60-channel EEG, electrooculography (EOG), and electromyography (EMG) signals were synchronously recorded. The dataset enables the exploration of neural activation patterns associated with lower-limb movements and supports the development of EEG-based brain–computer interface (BCI) algorithms for mobility assistance and rehabilitation. To validate the dataset, benchmark classification was conducted on three baseline deep learning methods--CTNet, EEGNet, and TCANet. Given the high inter-subject variability inherent to EEG, leave-one-subject-out cross-validation (LOSOCV) is used to ensure no subject bias during evaluation. Results demonstrated consistent decoding performance with mean accuracies of approximately 81\% for ME and 73\% for MI, indicating the reliability and usability of the dataset. Additionally, analyses of movement-related cortical potentials (MRCPs) and event-related desynchronization/synchronization (ERD/ERS) patterns revealed distinct neural signatures across the transition phases. This dataset provides a comprehensive foundation for studying lower-limb motor control, neural dynamics, and the advancement of MI-based BCIs for rehabilitation and assistive technologies.
**Corresponding Author:**	Theerawit Wilaiprasitporn, Ph.D.<br>Vidyasirimedhi Institute of Science and Technology<br>Rayong, Thailand THAILAND
**Corresponding Author Secondary Information:**	
**Corresponding Author's Institution:**	Vidyasirimedhi Institute of Science and Technology
**Corresponding Author's Secondary Institution:**	
**First Author:**	Benjakarn Uengsawapak
**First Author Secondary Information:**	
**Order of Authors:**	Benjakarn Uengsawapak
	Supavit Kongwudhikunakorn, Ph.D.
	Suktipol Kiatthaveephong
	Wipamas Polpakdee

Powered by Editorial Manager® and ProduXion Manager® from Aries Systems Corporation

	Rattanaphon Chaisaen
	Chanitsada Chuenchit
	Poramate Manoonpong, Ph.D.
	Gun Bhakdisongkhram, M.D, Ph.D.
	Theerawit Wilaiprasitporn, Ph.D.

**Order of Authors Secondary Information:**	
**Response to Reviewers:**	As attached PDF
**Additional Information:**	
**Question**	**Response**
Are you submitting this manuscript to a special series or article collection?	No
**Experimental design and statistics**<br><br>Full details of the experimental design and statistical methods used should be given in the Methods section, as detailed in our Minimum Standards Reporting Checklist. Information essential to interpreting the data presented should be made available in the figure legends.<br><br>Have you included all the information requested in your manuscript?	Yes
**Resources**<br><br>A description of all resources used, including antibodies, cell lines, animals and software tools, with enough information to allow them to be uniquely identified, should be included in the Methods section. Authors are strongly encouraged to cite Research Resource Identifiers (RRIDs) for antibodies, model organisms and tools, where possible.<br><br>Have you included the information requested as detailed in our Minimum Standards Reporting Checklist?	Yes
**Availability of data and materials**<br><br>All datasets and code on which the	Yes

conclusions of the paper rely must be either included in your submission or deposited in publicly available repositories (where available and ethically appropriate), referencing such data using a unique identifier in the references and in the "Availability of Data and Materials" section of your manuscript.<br><br>Have you have met the above requirement as detected in our Minimum Standards Reporting Checklist?	
GigaScience has policies and guidelines in place for the use of generative AI-writing tools such as ChatGPT. If you have used such writing tools to assist with writing the manuscript this must be declared and cited in the text. Authors should not list AI-writing tools and other AI-assisted technologies as an author or co-author and should acknowledge that they are fully responsible for text generated or refined by AI-writing tools.<p><br><br>A summary of use (particularly in the introduction or among methods) needs to be included at the end of the paper, and the outputs should also be included as a supplementary file hosted in GigaDB or other open repositories. Please read our guidelines for more information. <p><br><br>By submitting to GigaScience, you are aware of the journal's AI-writing tools policy, and if you have declared use of such tools below, you have acknowledged this where appropriate in your manuscript and have made a summary of use and outputs available. <p><br><b>AI-assisted writing tools have been used in the preparation of this manuscript?	No

Manuscript

Click here to access/download;Manuscript;eeg_sit_stand_final_version.pdf

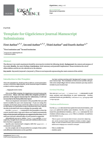

GigaScience, 2025, 1–12

doi: xx.xxxx/xxxx
Manuscript in Preparation
Data Note

DATA NOTE

EEG-Based Dataset Explicitly Targets the Transitions between Sitting and Standing for Exploring Neural Activation Patterns in Motor Imagery and Execution

Benjakarn Uengsawapak[1,*], Supavit Kongwudhikunakorn[2,*], Suktipol Kiatthaveephong[2], Wipamas Polpakdee[2], Rattanaphon Chaisaen[2], Chanitsada Chuenchit[3], Poramate Manoonpong[2], Gun Bhakdisongkhram[4,†] and Theerawit Wilaiprasitporn[2,†]

[1]School of Information Science and Technology (IST), Vidyasirimedhi Institute of Science and Technology (VISTEC), Rayong, Thailand,21210 and [2]Bio-inspired Robotics and Neural Engineering (BRAIN) Lab, School of Information Science and Technology (IST), Vidyasirimedhi Institute of Science and Technology (VISTEC), Rayong, Thailand,21210 and [3]Sirindhorn International Institute of Technology, Thammasat University, Pathum Thani, Thailand, 12120 and [4]School of Physical Medicine and Rehabilitation, Institute of Medicine, Suranaree University of Technology, Nakhon Ratchasima, Thailand, 30000

[*]These authors contributed equally to this work.
[†]Corresponding authors: G. Bhakdisongkhram (gunbhak@sut.ac.th) and T. Wilaiprasitporn (theerawit.w@vistec.ac.th)

Abstract

This study presents the first publicly accessible electroencephalography (EEG) dataset explicitly targeting sit-to-stand and stand-to-sit transitions during both motor execution (ME) and motor imagery (MI) tasks. Twenty-two healthy participants performed sitting and standing transitions under well-controlled experimental conditions while 60-channel EEG, electrooculography (EOG), and electromyography (EMG) signals were synchronously recorded. The dataset enables the exploration of neural activation patterns associated with lower-limb movements and supports the development of EEG-based brain–computer interface (BCI) algorithms for mobility assistance and rehabilitation. To validate the dataset, benchmark classification was conducted on three baseline deep learning methods–CTNet, EEGNet, and TCANet. Given the high inter-subject variability inherent to EEG, leave-one-subject-out cross-validation (LOSOCV) is used to ensure no subject bias during evaluation. Results demonstrated consistent decoding performance with mean accuracies of approximately 81% for ME and 73% for MI, indicating the reliability and usability of the dataset. Additionally, analyses of movement-related cortical potentials (MRCPs) and event-related desynchronization/synchronization (ERD/ERS) patterns revealed distinct neural signatures across the transition phases. This dataset provides a comprehensive foundation for studying lower-limb motor control, neural dynamics, and the advancement of MI-based BCIs for rehabilitation and assistive technologies.

Key words: EEG dataset; lower-limb motor imagery; lower-limb motor execution; sit-to-stand transition; stand-to-sit transition; brain–computer interface (BCI); event-related desynchronization (ERD); movement-related cortical potential (MRCP)

Data Description

Compiled on: May 23, 2026.
Draft manuscript prepared by the author.

Key Points

- The publicly available EEG dataset capturing sit-to-stand and stand-to-sit transitions, with a focus on lower-limb movement.
- Multi-modal recordings (EEG, EOG, EMG) validated by CNN-based and Transformer-based models for MI and ME decoding.
- Provides benchmark framework and neurophysiological insights for lower-limb MI-BCI research.

Background and Purpose

Motor imagery (MI), the mental simulation of movement without physical execution, is a central yet challenging paradigm in electroencephalography (EEG)-based brain–computer interfaces (BCIs). MI-BCIs harness intentional brain activity to control external devices, such as assistive tools and computers, purely through thought. This paradigm shows strong potential in rehabilitation, neuroprosthetics, and assistive technologies, particularly for individuals with motor impairments, including stroke survivors and patients with neurodegenerative diseases [1]. Typically, MI-BCIs involve imagery of specific movements, such as hand or foot actions, that activate sensorimotor brain regions. These activations produce distinctive EEG patterns in the 8–30 Hz frequency range, characterized by event-related desynchronization (ERD)—a reduction in amplitude before or during the event—and event-related synchronization (ERS)—an increase in amplitude afterward. These rhythms form the foundation for decoding motor intentions for neurorehabilitation applications.

Beyond hand and foot movements, investigating the neural signals associated with sit-to-stand (sit–stand) and stand-to-sit (stand–sit) transitions provides valuable insights for developing mobility rehabilitation protocols and assistive devices. Recent studies have demonstrated the feasibility of utilizing EEG signals during sit–stand and stand–sit imagery to design MI-BCI systems that support lower-limb movement by accurately decoding motor intentions [2, 3, 4, 5, 6]. Building on these findings, MI-BCIs for this tasks hold promise for real-world applications, allowing smoother transitions between sitting and standing positions.

Despite its potential, sit-stand and stand-sit transitions remain underrepresented in MI-BCI research. Current publicly available MI-BCI EEG datasets predominantly focus on traditional hand and foot movements due to their well-defined and distinctive neural patterns [7, 8]. While these datasets have supported the development of advanced EEG-based classification algorithms, none offers EEG recordings of transitional sit-stand or stand-sit motor imagery. The lower-limb movement datasets have been well presented in the study by Triana-Guzman et.al [5]; however, their main objective lies on classifying completed movement task. In practical BCI scenarios, distinguishing transition-related neural activity from initial resting states and detecting early movement intention are critical challenges. This gap underscores the need for dedicated datasets focusing on these transitions to better capture lower-limb dynamics and expand the application of MI-BCIs in rehabilitation.

To advance research in this area, we present an EEG dataset comprising recordings from 22 participants during motor imagery (MI) and motor execution (ME) of sit-stand and stand-sit transitions, featuring up to 60 EEG channels. In addition to EEG, electrooculography (EOG) was collected to support artifact removal related to eye movements during standard preprocessing, and electromyography (EMG) was recorded concurrently to precisely determine movement onset during ME trials. To the best of our knowledge, this is the first publicly accessible EEG dataset explicitly designed to target sit-stand and stand-sit transitions. It provides a valuable resource for researchers investigating lower-limb motor intentions reflected in EEG activity and developing EEG-based algorithms dedicated to lower-limb MI-BCIs. This dataset directly contributes a significant step in publicly available MI-BCI resources to advance lower-limb research and applications.

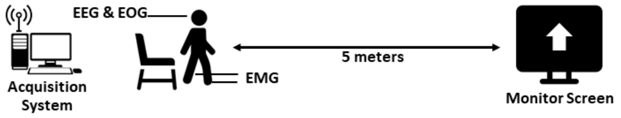

Figure 1. Experimental setup for data collection. A monitor displaying visual cues was placed in front of the participant, while the data acquisition system for synchronous EEG, EOG, and EMG recording operated by research staff was behind the participant.

Experimental Design

Participants

Twenty-three healthy participants (aged 22–28 years; fifteen males) with no known neurophysiological abnormalities were recruited for this study. One participant (S05) was excluded due to poor signal quality, resulting in a final cohort of twenty-two participants. Prior to the experiment, research staff provided both verbal and written explanations of the study objectives, protocol, questionnaire, and experimental setup to ensure participants' understanding. A written consent form was obtained from all participants in accordance with the Declaration of Helsinki. Participants received monetary compensation for their involvement. The experimental protocol and environment were reviewed and approved by the Ethics Committee of Suranaree University of Technology, Thailand (EC-65-0031). The demographic of the subject and the questionnaire used in this study are provided in the supplementary material.

Environment

All experiments were conducted in a quiet, controlled environment with only the participant and research staff present. The experimental setup is illustrated in Figure 1. For the sitting state, participants were seated approximately 5 meters from a 65-inch wall-mounted 4K LED monitor, oriented toward the monitor displaying visual cues. For the standing state, participants positioned themselves directly in front of the chair. The data acquisition system comprising a desktop computer running Windows 11, equipped with a 24-inch monitor, keyboard, and mouse, was placed on a table behind the participant. Research staff operated the system to synchronously record EEG, EOG, and EMG signals while ensuring the correct sequence of visual cues. To minimize fatigue effects, participants were instructed to rest adequately the night before, and all data collection sessions were scheduled during morning hours to ensure an optimal physiological state.

Data acquisition

Data Collection Protocol

Data acquisition was conducted in two identical sessions of motor tasks, separated by a 5–10 minute rest period. At the beginning of each session, baseline brain activity was recorded under two resting-state conditions: one minute with eyes closed (EC) followed by one minute with eyes open (EO). Participants then performed the ME task (40 trials per session), after which they completed the MI tasks. The MI tasks alternated between sitting and standing conditions and were repeated twice per session (20 trials per session). The overall procedure—including participant preparation, system setup, and data collection across both sessions—lasted approximately two hours per participant. The flow of the data collection protocol is visualized in Figure 2a.

Motor Execution (ME)

After collecting baseline EEG at the beginning of each session, participants performed the ME task, in which they carried out physical sit-stand (ME_SIT_STD) and stand-sit (ME_STD_SIT) movements. The task consisted of 40 trials, alternating between sit-stand and stand-sit transitions. For sit-stand trials, participants began seated on a chair, whereas for stand-sit trials, participants began standing in front of a chair. Each trial lasted 16 seconds, resulting in a total duration of 640 seconds (approximately 11 minutes), as shown in Figure 2b.

The structure of each trial was as follows: a fixation cross appeared for 2 seconds to direct participants' gaze and signal trial preparation. A visual cue was then presented for 1 second: an upward arrow instructed participants to stand up from a seated position, while a downward arrow instructed them to sit down from a standing position. After the cue disappeared, participants executed the instructed movement within 5 seconds. Another fixation cross then appeared for 2 seconds, followed by a 1-second presentation of a white circle, instructing participants to rest (ME_R) in their current posture for the subsequent 5 seconds. This marked the completion of one trial.

Motor Imagery (MI)

Following the ME task, participants performed the MI task under two conditions: during sitting and standing. In contrast to the previous task, participants were instructed to mentally simulate the sit-stand and stand-sit transitions without executing any physical movement.

Motor Imagery during Sit

In the MI during sit condition (Figure 2c), participants sat on a chair and observed sequential visual cues. Each 16-second trial began with a white cross for 2 seconds, prompting participants to focus and prepare for the upcoming cue. Subsequently, a 1-second visual cue appeared—a white upward arrow indicating participants should imagine standing up from sitting (MI_SIT_STD) or a white downward arrow indicating participants should imagine sitting down while already seated (MI_SIT_SIT). Participants imagined these movements for 5 seconds immediately after the cue disappeared. Another white cross appeared for 2 seconds, signaling preparation for the next cue, followed by a 1-second white circle instructing participants to rest. Participants then rested for 5 seconds (MI_R_SIT) while remaining seated to prevent fatigue.

MI during sit was divided into two nonconsecutive rounds, each consisting of 20 pseudorandomized trials (10 for MI_SIT_STD and 10 for MI_SIT_SIT). In the remaining part of this study, the MI_SIT_SIT task data is not analyzed, as they do not involve directional motor transitions and are expected to produce neural signatures closely resembling resting states. However, the data are provided to enable further exploration in potential studies, such as the detection of a user's static postural intentions.

Motor Imagery during Stand

In the MI during stand condition (Figure 2d), participants stood in front of a chair while observing sequential visual cues. Each 16-second trial began with a white cross displayed for 2 seconds, signaling participants to prepare for the upcoming instruction. Next, a 1-second visual cue appeared—a white downward arrow instructing participants to imagine sitting down from standing (MI_STD_SIT) or a white upward arrow instructing participants to imagine standing up while already standing (MI_STD_STD). Participants imagined these movements for 5 seconds immediately after the cue disappeared. Another white cross appeared for 2 seconds, signaling preparation for the next cue, followed by a 1-second white circle instructing participants to rest. Participants then rested for 5 seconds (MI_R_STD) while remaining standing to avoid fatigue.

MI during stand was divided into two nonconsecutive rounds, each consisting of 20 pseudorandomized trials (10 for MI_STD_SIT and 10 for MI_STD_STD). In the remaining part of this study, the MI_STD_STD task data is not analyzed, as they do not involve directional motor transitions and are expected to produce neural signatures closely resembling resting states. However, the data are provided to enable further exploration in potential studies, such as the detection of a user's static postural intentions.

EEG Signals

Electroencephalography (EEG) recordings were acquired using a biosignal amplifier (g.HIamp, g.Tec, Austria) with a sampling rate of 1,200 Hz from 62 electrodes (60 EEG, 2 EOG) arranged according to the international 10–20 system. Of these, EEG signals were recorded using 60 electrodes, while horizontal (hEOG) and vertical (vEOG) EOG signals were acquired from two additional electrodes, as described in the subsequent section. The 60 EEG channels recorded included Fp1, Fp2, AF7, AF8, F7, F8, FT7, FT8, AF3, AF4, AFz, Fz, F1, F2, F3, F4, F5, F6, FCz, Cz, FC1, FC2, FC3, FC4, FC5, FC6, C1, C2, C3, C4, C5, C6, CPz, Pz, CP1, CP2, CP3, CP4, CP5, CP6, TP7, TP8, P1, P2, P3, P4, P5, P6, P7, P8, POz, Oz, PO3, PO4, PO7, PO8, PO9, PO10, O1, and O2.

The FPz electrode was used as the ground electrode. Signals from the left and right earlobes were averaged and applied to other channels as references. Throughout the experiment, electrode impedance was maintained below 30 $k\Omega$, and conductive gel was applied as needed. Electrode placement is depicted in Figure 3.

EOG Signals

The two electrodes described in the previous EEG subsection were dedicated to recording electrooculography (EOG) signals. One electrode was placed on the right temple (designated as EOG-1, or hEOG, using channel #61), while the other was positioned on the right infraorbital region (designated as EOG-2, or vEOG, using channel #62), as illustrated in Figure 3. In this study, EOG signals were used to assist in ocular artifact removal during EEG data preprocessing.

EMG Signals

Electromyography (EMG) signals were collected using six surface EMG sensors (Trigno Avanti Sensor, Delsys, USA) at a sampling rate of 2,000 Hz. Sensors were attached bilaterally to three lower-limb muscles: the Soleus (SL), Tibialis Anterior (TA), and Rectus Femoris (RF). The locations of EMG recording sensors are illustrated in Figure 4. In this study, EMG signals are used to assist in locating the movement onset of ME activities.

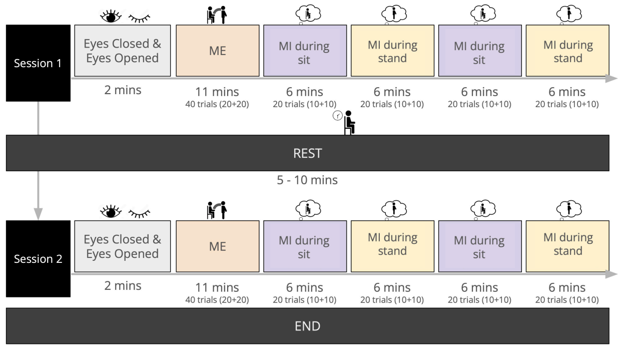

(a) Data Collection Protocol.

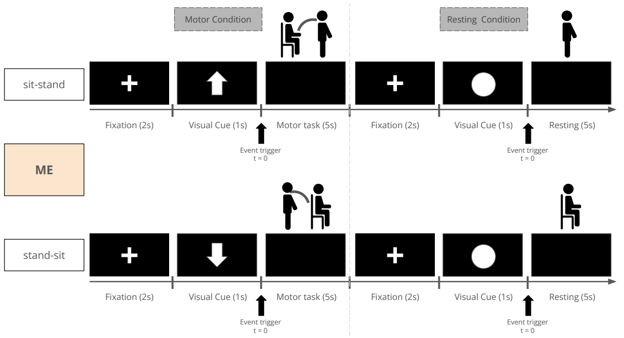

(b) Motor Execution (ME) activity. Each round alternated 20 sit-stand and 20 stand-sit trials, followed by a resting task.

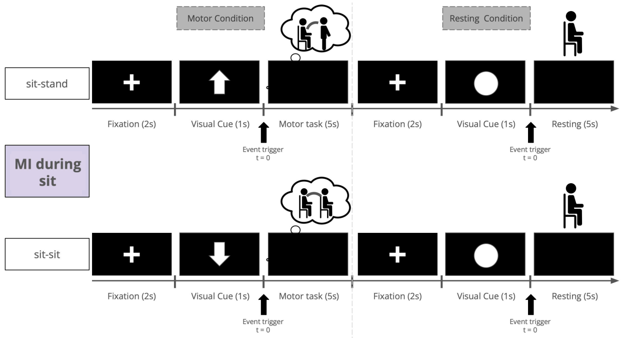

(c) Motor Imagery (MI) during sit. Each round included 10 sit-stand and 10 sit-sit trials in a pseudorandom order, followed by a resting task.

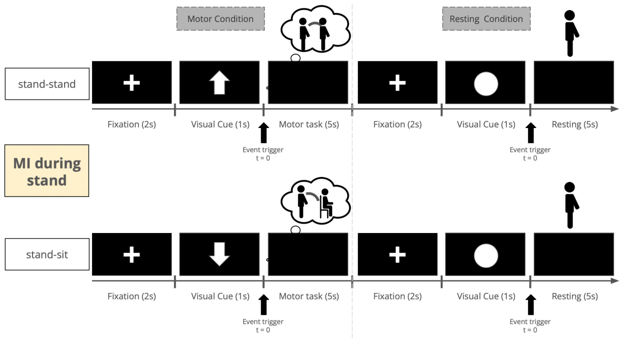

(d) Motor Imagery (MI) during stand. Each round included 10 stand-sit and 10 stand-stand trials in a pseudorandom order, followed by a resting task.

Figure 2. Overview of the data collection process and task-related instructions. Fig 2a shows overall protocol; highlighted regions are detailed in (b–d). Fig 2b shows visual cue instructions for motor execution (ME). Fig 2c shows visual cue instructions for motor imagery (MI) during sit. Fig 2d shows visual cue instructions for MI during stand.

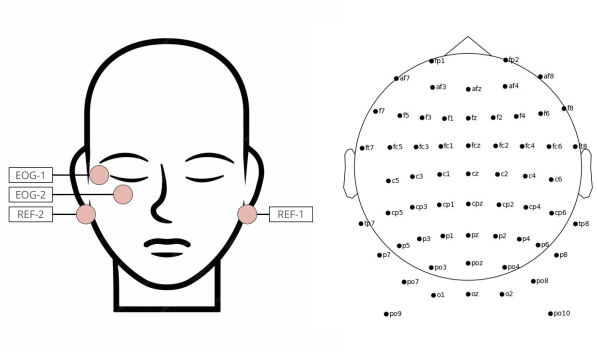

Figure 3. Electrodes placement positions for recording EEG and EOG signal.

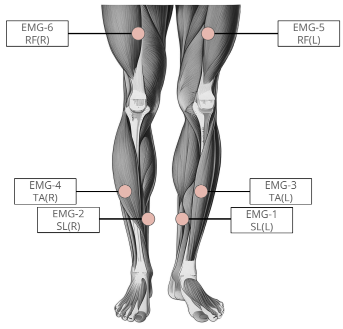

Figure 4. Surface EMG sensor placement positions for recording EMG signal.

Data Synchronization

All recorded physiological signals (EEG, EOG, and EMG) were synchronized with timestamps generated by the data acquisition system, operating on the Windows 11 operating system, prior to preprocessing and analysis.

Data format and structure

To enhance usability, both raw data in *.mat* format and preprocessed data in *.fif* format are provided. It is recommended to use the Python-based MNE library [9] for full access to *.fif* files. A detailed description of the datasets is presented in Table 1. This study focuses on EEG recordings collected while participants engaged in various BCI motor-related activities, including motor execution (ME), motor imagery (MI) during sit, and MI during stand. Additionally, baseline EEG recordings were acquired during resting-state conditions, specifically during eyes-closed (EC) and eyes-opened (EO), prior to the execution of motor tasks. These baseline recordings are included to facilitate further analysis. According to Figure 2a, each experimental session consisted of a single run for ME, EC, and EO. In contrast, each MI condition—MI during sit and MI during stand—was recorded twice per session. Consequently, two separate files were generated for each session of MI during sit, and MI during stand, labeled as *_S1* and *_S2*. The raw EEG data for each participant are structured as a matrix of dimensions $n\_channels \times n\_timepoints$. An extra channel (channel #63) is allocated for event triggers that annotate instruction-related events and serve as ground truth labels. The processed EEG data are structured as a three-dimensional matrix of size $n\_trials \times n\_channels \times n\_timepoints$. A detailed description of event trigger codes is provided in Table 2.

Name	Description
	**Raw Data**
S<ID>_S<$session_{num}$>.mat	Raw signal recorded from subject *ID* in session $session_{num}$
S01_S1.mat	*Example:* Raw signal recorded from subject 01 in session 1
	**Processed Data**
S<ID>.fif	Pre-processed signal from subject *ID*.
S01.fif	Pre-processed signal from subject 01.

Table 1. Data description table.

Event	Description
1	Eyes closed (beginning of session).
2	Eyes opened (beginning of session).
10	Start of trials in ME activity.
11	Start of ME_SIT_STD in ME activity.
12	Start of ME_STD_SIT in ME activity.
13	Start of resting task (ME_R) in ME activity.
20	Start of trials in MI during sit.
21	Start of MI_SIT_STD in MI during sit.
22	Start of MI_SIT_SIT task in MI during sit.
23	Start of resting task in MI during sit (MI_R_SIT, rest while sitting).
30	Start of trials in MI during stand.
31	Start of MI_STD_STD task in MI during stand.
32	Start of MI_STD_SIT in MI during stand.
33	Start of resting task in MI during stand (MI_R_STD, rest while standing).

Table 2. Event trigger number description table. The event trigger is stored at EEG channel number #63 in the .mat file and embedded into MNE Epochs from the .fif file.

Data Validation

Data Preprocessing

This study's EEG recordings were preprocessed to analyze brain activity during EC, EO, MI, and ME activities. Specifically, movement-related cortical potential (MRCP) features were examined, predominantly associated with ME activities. Conversely, time-frequency power distribution features were analyzed for EC, EO, and MI activities, as recommended and validated in previous studies [4, 10]. Due to these differences, the preprocessing pipelines for these activities varied slightly, as described in detail below. All preprocessing steps were conducted using the *MNE-Python* library [9].

Preprocessing Steps for Movement-Related Cortical Potential (MRCP) Analysis

For MRCP feature extraction in ME-based classification, the following preprocessing steps were applied:

The 60-channel EEG recordings were first filtered with a second-order Butterworth bandpass filter with a 0.2 to 3 Hz cutoff frequency. Subsequently, the signals were downsampled to 250 Hz. To remove artifacts, independent component analysis (ICA) was performed to decompose independent components (ICs) and eliminate artifacts using *mne.preprocessing.ICA* in *MNE-Python* library. To assist the ICA process, the eye-related artifacts were eliminated using recorded EOG signals, while the muscle-related artifacts were eliminated using the function *find_bads_muscle()*. Following, signals from identified bad channels were removed and interpolated using data from neighboring electrodes. To mitigate volume conduction effects and enhance spatial resolution, the Current Source Density (CSD) transformation was applied [11]. Finally, the preprocessed signals were segmented into 4-second epochs ranging from -2 to 2 seconds relative to an onset of EMG trigger event (indicating $T = 0$). (Note: The EMG data from session #2 of subject #20 is unavailable. To resolve this issue, specifically for this circumstance, the 4-second epochs were segmented using the onset of the event triggers #11 and #12 obtained from channel number #63.) Furthermore, we excluded those trials contaminated by noise and amplitude spikes with a trial rejection based on peak-to-peak (PTP) amplitude calculation of EEG signals, as those trials with large PTP amplitudes indicate the presence of artifacts. Any trials with the PTP amplitudes exceeding this 95th percentile threshold are automatically flagged and rejected from further analysis. On average, 36 trials remained after rejection. Thus, the first 36 trials were selected for further analysis.

For ME-based classification, each trial was labeled according to a participant's physical movement, either ME activities or resting task (ME_R). The ME resting trials are alternately separated into ME_R_SIT (rest while sitting) and ME_R_STD (rest while standing), respectively. Start from index 0, the even-numbered trials are labeled as ME_R_STD, while the odd-numbered trials are labeled as ME_R_SIT. Similarly, trials labeled as ME_SIT_STD and ME_STD_SIT correspond to a participant performing a sit-stand and stand-sit transition, respectively.

Preprocessing Steps for Time-Frequency Distribution Analysis

Preprocessing for MI-based classification, including EC and EO, was similar to that used for MRCP analysis, with the following modifications:

Instead of using a second-order Butterworth bandpass filter (0.2–3 Hz), a sixth-order Butterworth bandpass filter with 1–40 Hz cutoff frequencies was applied. The processed signals were also segmented into trials ranging from -2 to 5 seconds relative to the event trigger onset.

In contrast to the ME task, the MI task did not involve any actual movement; therefore, no EMG onset was observed. The preprocessed signals were segmented into epochs relative to the onset of the event trigger #21 and #32, as described in Table 2. Each trial spanned 7 seconds (2 seconds before and 5 seconds after the event trigger onset). In order to minimize the influence of ongoing background activity, a 2-second segment before the event trigger was used for baseline correction, leaving a 5-second segment for further analysis. We excluded those trials contaminated with noise by a threshold-based trial rejection using PTP amplitude calculation, similar to the steps for MRCP analysis. On average, 36 trials remained after rejection and were selected for further analysis. For MI-based classification, trials were labeled according to participants' current physical states. MI_R_SIT and MI_R_STD corresponded to a participant resting while sitting and standing, respectively. Similarly, trials labeled as MI_SIT_STD and MI_STD_SIT corresponded to the participant imagining sit-stand and stand-sit transitions, respectively.

It should be noted that the EEG data preprocessed with ICA and CSD were utilized for visualization, whereas data without these preprocessing steps were used for classification with CTNet, EEGNet, and TCANet models. To confirm the quality of the signals cleaned from the ICA, we compare the signals before and after the cleaning, as shown in Figure 5. From the figure, we observed that the data after cleaning (shown in black) exhibits lower fluctuation and artifactual components compared to the data before cleaning (shown in red), which exhibits high-frequency components and appears as sharp burst-like waveforms. In addition, our deep learning-based classification experiments were conducted on segmented, filtered EEG time-series data, based on the assumption that the state-of-the-art models are designed in an end-to-end style.

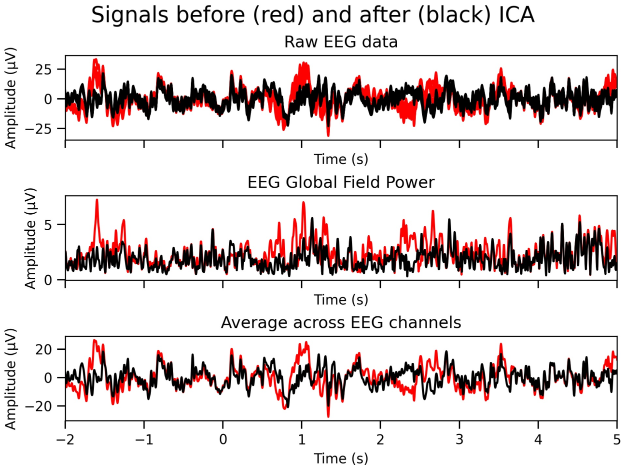

Figure 5. Comparison of EEG signals before (shown in red line) and after (shown in black line) independent component analysis (ICA). The top visualization compares the EEG signals from all channels. The middle visualization compares the EEG global field power from all channels. The bottom visualization compares the average of EEG signals across all channels.

Method

Classification Techniques and Models

To validate the correctness of the proposed dataset, ME- and MI-based classification experiments were conducted using CTNet[12], EEGNet[13], and TCANet[14] models, the commonly used deep learning baseline models in BCI classification studies. EEG signals, like other biological signals, exhibit high inter-subject variability, making subject-independent classification crucial for broader BCI applications [15]. To avoid subject bias during evaluation, leave-one-subject-out cross-validation (LOSOCV) is employed to ensure the test data remain unseen during training. EEG recordings from each subject were treated as independent samples, enhancing robustness against inter-subject variability. Accordingly, the data input shape to train and validate the model is $n_{subject} - 1 \times n_{trial} \times n_{channel} \times n_{timepts}$, while the data input shape to test the model is $1 \times n_{trial} \times n_{channel} \times n_{timepts}$, where $n_{subject}$ represents total number of subjects, n_{trial} represents total number of trials that each subject performed, $n_{channel}$ represents total number of EEG channels, $n_{timepts}$ represents length of EEG samples. All three models were trained using cross-entropy loss for up to 200 epochs, with early stopping triggered if the loss did not improve for 10 consecutive epochs. A 5-fold cross-validation scheme was applied to ensure unbiased and optimal performance. The parameter settings for each model are as follows:

CTNet [12]: A convolutional transformer network designed for EEG-based classification of motor imagery. The first layer of CTNet employs a convolutional module to extract local and spatial EEG features. In contrast, the Transformer encoder module is employed in the subsequent layer to learn global dependencies in high-level EEG features. CTNet shows remarkable decoding accuracies for both subject-specific (subject-dependent) and cross-subject evaluations. Our implementation uses Python with *PyTorch* [16], *scikit-learn* [17], and *mne* [9] libraries. In this study, the parameter settings of CTNet are as follows: number of attention heads $n_{head} = 4$, embedding size $emb\_size = 40$, depth $d = 6$, kernel size $k = 64$. The optimal batch size b and the learning rate lr are set as $b = 8$ and $lr = 1 \times 10^{-3}$, respectively.

EEGNet-8,2: EEGNet [13] effectively learns spatiotemporal EEG features through a compact convolutional neural network. It employs depthwise and separable convolutions to enhance feature learning while reducing trainable parameters, improving efficiency without sacrificing classification performance in EEG-based BCI tasks. Our implementation uses *Python* with *PyTorch* and *scikit-learn*. In this study, the parameter settings of EEGNet-8,2 are as follows: number of filters in the first layer $F1 = 8$, depth parameter $D = 2$, kernel size $C1 = 200$, number of classes $n_{class} = 2$, dropout rate $r_{dropout} = 0.5$. The optimal batch size b and learning rate lr are set to be $b = 8$ and $lr = 1 \times 10^{-3}$, respectively.

TCANet [14]: A multi-scale temporal convolutional attention network designed for EEG-based classification of motor imagery. The first layer adopts a multi-scale convolutional module to extract local spatiotemporal features across different temporal resolutions. Subsequently, the temporal convolutional module combines and compresses these multi-scale features. Finally, the multi-head self-attention mechanism learns global dependency features in the EEG. Our implementation uses Python with *PyTorch*, *scikit-learn*, and *mne* libraries. In this study, the parameter settings of TCANet are as follows: filter size $f_1 = 16$, pooling size $pooling = 56$, number of attention heads $n_{head} = 2$, depth $d = 6$, dropout rate $r_{dropout} = 0.25$. The optimal batch size b and learning rate lr are set to be $b = 8$ and $lr = 1 \times 10^{-3}$, respectively.

Experiments

This study presents BCI classification in two experimental scenarios—ME ad MI—as the analysis approaches are varied.

Experimental Design for Experiment 1: the MRCP analysis for ME-based classification

For ME-based classification, this study focuses on analyzing and decoding the subjects' movement intention from Movement-Related Cortical Potential (MRCP), a low-frequency (less than 3 Hz) cortical potential characterized by a negative shift in the EEG signal. MRCP is observed before the onset of real movement and used to detect motor intention, with a direct association to primary motor and somatosensory cortices [18, 19]. As the MRCP is a premovement cortical potential, it usually occurs within 2 seconds prior to movement onset. Accordingly, the 2-second time windows before movement were selected to capture pre-movement brain activity and to evaluate their predictive performance for classification.

In this study, we concentrate on classifying the ME task during transition and resting task using premovement signals. Specifically, we classify EEG recordings in two tasks: (1) ME_SIT_STD vs. ME_R_SIT and (2) ME_STD_SIT vs. ME_R_STD. To determine the optimal EEG segment length for further applications, we trained the three baseline models on two distinct EEG time windows: one second before movement and two seconds before movement. For example, the data input shape for this binary-classification experiment for 2-second data is 22 *subjects* × 72 *trials* × 60 *channels* × 500 *timepts*.

Experimental Design for Experiment 2: the time-frequency analysis for MI-based classification

For MI-based classification, this study analyzes and decodes subjects' movement imagination from the EEG time–frequency distribution in the 1–40 Hz range. We classify EEG recordings in two tasks: (1) MI_SIT_STD vs. MI_R_SIT, (2) MI_STD_SIT vs. MI_R_STD. To study the optimal EEG segment length and evaluate its predictive performance for classification for further applications, we trained and evaluated on five distinct EEG time windows: 1, 2, 3, 4, and 5 seconds after stimulus onset. The example of data input shape for this binary-classification experiment for 5-second data is 22 *subjects* × 72 *trials* × 60 *channels* × 1250 *timepts*.

Performance Matrix and Evaluation

In this study, classification performance is assessed and reported in terms of accuracy (ACC) to measure the correct classification rate, F1-score (F1) to assess the balance between precision and recall, and area under the curve (AUC) that provides useful insights on the model's ability to handle robustness of class-imbalance classification. For insights on the classifier's performance, confusion

Table 3. Classification performance of three baseline methods (CTNet, EEGNet, TCANet), along with training T_{train} and inference T_{infer} times (in seconds) per one fold, on the proposed dataset for motor execution (ME) and motor imagery (MI) tasks using different EEG segment lengths (Mean ± SD).

Experiment	Task	Segment Length (s)	Accuracy ↑	F1-score ↑	AUC ↑	T_{train} (s) ↓	T_{infer} (s) ↓
			**Method: CTNet**				
ME	ME_SIT_STD vs ME_R_SIT	1	77.35 ± 8.78*	74.37 ± 12.34*	0.8570 ± 0.0939*	**413.00 ± 37.31**	**0.39 ± 0.04**
		2	**81.25 ± 8.30**	**80.34 ± 9.64**	**0.8996 ± 0.0766**	610.73 ± 488.64*	0.59 ± 0.63
	ME_STD_SIT vs ME_R_STD	1	77.60 ± 9.57*	74.95 ± 13.32	0.8643 ± 0.0894*	**410.64 ± 41.86**	**0.39 ± 0.07**
		2	**79.89 ± 9.52**	**78.98 ± 11.00**	**0.8912 ± 0.0903**	602.05 ± 493.91*	0.60 ± 0.58*
MI	MI_SIT_STD vs MI_R_SIT	1	71.41 ± 8.48	68.31 ± 13.53	0.7889 ± 0.0986	**160.61 ± 9.20**	**0.09 ± 0.00**
		2	**71.93 ± 8.30**	**70.19 ± 12.01**	0.8053 ± 0.0903	164.70 ± 11.78	0.09 ± 0.00*
		3	71.23 ± 8.81	69.74 ± 13.63	0.8082 ± 0.0987	241.26 ± 16.86*	0.23 ± 0.02*
		4	70.31 ± 9.80	68.29 ± 16.37	0.7986 ± 0.1073	258.53 ± 19.47*	0.24 ± 0.02*
		5	70.77 ± 8.45	68.69 ± 13.98	**0.8136 ± 0.0921**	2566.27 ± 134.43*	3.89 ± 0.46*
	MI_STD_SIT vs MI_R_STD	1	72.79 ± 8.23	70.24 ± 12.43	0.8073 ± 0.0829	**159.37 ± 8.15**	**0.09 ± 0.00**
		2	72.80 ± 8.32	70.48 ± 13.51	0.8157 ± 0.0874	162.18 ± 9.88	0.09 ± 0.00*
		3	**73.08 ± 10.15**	**70.56 ± 17.15**	**0.8240 ± 0.0914**	240.23 ± 13.91*	0.23 ± 0.03*
		4	71.95 ± 11.23	69.24 ± 18.39	0.8209 ± 0.1045	256.81 ± 20.99*	0.23 ± 0.02*
		5	71.55 ± 11.49	67.35 ± 18.94	0.8125 ± 0.1073	2454.36 ± 139.27*	3.63 ± 0.51*
			**Method: EEGNet**				
ME	ME_SIT_STD vs ME_R_SIT	1	75.91 ± 9.38*	70.87 ± 14.84*	0.8210 ± 0.1091*	70.01 ± 9.33*	0.06 ± 0.01
		2	**79.85 ± 7.99**	**77.60 ± 10.47**	**0.8844 ± 0.0751**	**63.37 ± 7.63**	**0.06 ± 0.01**
	ME_STD_SIT vs ME_R_STD	1	76.02 ± 9.68*	71.68 ± 15.62*	0.8302 ± 0.0984*	**61.40 ± 5.53**	0.06 ± 0.01
		2	**79.68 ± 8.80**	**77.93 ± 11.88**	**0.8866 ± 0.0836**	63.22 ± 6.65	**0.06 ± 0.01**
MI	MI_SIT_STD vs MI_R_SIT	1	69.16 ± 8.62	67.48 ± 13.28	0.7677 ± 0.0981	**86.96 ± 12.95**	0.08 ± 0.01
		2	**69.58 ± 8.86**	69.69 ± 11.15	0.7994 ± 0.0973	92.32 ± 15.79	**0.07 ± 0.01**
		3	68.98 ± 9.56	**70.70 ± 11.84**	**0.8045 ± 0.0975**	155.97 ± 27.96*	0.14 ± 0.03*
		4	67.85 ± 10.24	69.58 ± 12.91	0.7992 ± 0.1016	168.59 ± 32.24*	0.13 ± 0.04*
		5	67.39 ± 10.25	69.16 ± 14.02	0.7985 ± 0.1031	167.96 ± 25.68*	0.14 ± 0.03*
	MI_STD_SIT vs MI_R_STD	1	71.37 ± 7.44	68.73 ± 13.24	0.7872 ± 0.0969	**83.03 ± 11.12**	0.07 ± 0.01
		2	71.27 ± 8.33	**70.15 ± 13.16**	0.8022 ± 0.1001	95.65 ± 13.74*	**0.07 ± 0.01**
		3	70.88 ± 10.82	68.77 ± 19.01	0.8080 ± 0.1204	124.96 ± 24.34*	0.08 ± 0.03
		4	**71.42 ± 11.13**	68.58 ± 20.98	**0.8149 ± 0.1129**	149.74 ± 19.01*	0.07 ± 0.01
		5	70.63 ± 11.54	67.51 ± 21.92	0.8049 ± 0.1215	148.15 ± 14.33*	0.07 ± 0.02
			**Method: TCANet**				
ME	ME_SIT_STD vs ME_R_SIT	1	77.93 ± 8.95	74.44 ± 12.70	0.8532 ± 0.0987*	230.21 ± 31.27*	**0.09 ± 0.00**
		2	**81.15 ± 8.36**	**79.81 ± 10.38**	**0.8958 ± 0.0700**	**192.74 ± 19.02**	0.10 ± 0.00*
	ME_STD_SIT vs ME_R_STD	1	78.69 ± 9.88	74.84 ± 15.55	0.8628 ± 0.0885	237.31 ± 25.10*	**0.09 ± 0.01**
		2	**80.86 ± 8.83**	**79.65 ± 11.44**	**0.8993 ± 0.0770**	**216.13 ± 24.89**	0.10 ± 0.00*
MI	MI_SIT_STD vs MI_R_SIT	1	70.48 ± 9.61	67.88 ± 15.24	0.7776 ± 0.1059*	**300.23 ± 42.52**	**0.10 ± 0.00**
		2	**72.06 ± 7.87**	70.24 ± 11.43	0.8090 ± 0.0893	308.52 ± 35.18	0.10 ± 0.01*
		3	71.66 ± 8.83	**70.47 ± 13.13**	**0.8222 ± 0.0899**	446.30 ± 54.60*	0.24 ± 0.02*
		4	70.77 ± 9.73	69.40 ± 15.25	0.8162 ± 0.0908	416.40 ± 49.81*	0.25 ± 0.02*
		5	69.98 ± 9.11	68.70 ± 15.46	0.8102 ± 0.0906*	397.43 ± 69.92*	0.24 ± 0.04*
	MI_STD_SIT vs MI_R_STD	1	73.25 ± 8.34	71.78 ± 11.84	0.8113 ± 0.0940	313.94 ± 39.21	**0.09 ± 0.00**
		2	72.92 ± 7.58	71.88 ± 10.91	0.8162 ± 0.0885	**312.73 ± 35.05**	0.10 ± 0.00*
		3	**73.60 ± 9.33**	**72.12 ± 14.28**	0.8354 ± 0.0897	439.43 ± 91.45*	0.22 ± 0.06*
		4	73.21 ± 10.94	70.98 ± 17.59	0.8388 ± 0.0948	447.48 ± 57.71*	0.24 ± 0.02*
		5	73.53 ± 10.52	71.23 ± 17.18	**0.8389 ± 0.0969**	419.95 ± 50.11*	0.24 ± 0.02*

Note: * indicates statistically significant difference using a t-test ($p \leq 0.05$) compared to the best-performing setting, highlighted in bold. Segment length refers to the EEG window duration in seconds, extracted relative to stimulus onset (before onset for ME, after onset for MI).
Abbreviations: ME_SIT_STD, executing standing up from sitting; ME_R_SIT, rest while sitting; ME_STD_SIT, executing sitting down from standing; ME_R_STD, rest while standing; MI_SIT_STD, imagining standing up from sitting; MI_R_SIT, rest while sitting; MI_STD_SIT, imagining sitting down from standing; MI_R_STD, rest while standing; T_{train}, training time; T_{infer}, inference time (testing time).

matrices of the best model are also provided.

Qualitative Analysis

In addition to the reported classification performance, to analyze activation of EEG spatially in response to the onset of the visual stimulus, we have visualized topographical maps of transition against resting task in MI activity across different tasks and frequency rhythms, as presented in the upcoming section. These visualizations provide readers with additional insights into how the brain state changes over different time intervals compared with the base-

line. Additionally, to select the suitable interval for motor classification, experiments on variations of EEG segment length provide useful insights to assist readers in further studies and analysis.

Results and Discussion

Performance Analysis

This section presents classification performance for both ME and MI activities. For ME activity, we focused on classifying EEG signals obtained from two tasks–ME_SIT_STD vs. ME_R_SIT (classifying EEG signals between motor execution during sit-stand transition and rest while sitting) and ME_STD_SIT vs. ME_R_STD (classifying EEG signals between motor execution during stand-sit transition and rest while standing). To assess the robustness of the dataset, we evaluated performance across different EEG segment lengths using three baseline deep learning models commonly applied in BCI studies: CTNet, EEGNet, and TCANet.

We evaluated our dataset by assessing the performance of three baseline models in terms of classification results and running time, illustrated in Table 3. It can be seen that TCANet generally performs substantially better than the other methods. In ME_SIT_STD vs. ME_R_SIT task, TCANet attained the best accuracy of $81.15 \pm 8.36\%$, $79.81 \pm 10.38\%$ in terms of F1-score, and 0.8958 ± 0.0700 in terms of AUC, on the 2-second segment, with the average training time 192.74 ± 19.02 seconds per one fold and average inference time 0.10 ± 0.00 second. In ME_STD_SIT vs. ME_R_STD task, TCANet also attained the best accuracy of $80.86 \pm 8.83\%$, $79.65 \pm 11.44\%$ in terms of F1-score, and 0.8993 ± 0.0770 in terms of AUC, on the 2-second segment, with the average training time 216.13 ± 24.89 seconds per one fold and average inference time 0.10 ± 0.00 second.

Similarly, EEG signals from MI activity were classified for two binary tasks: MI_SIT_STD vs. MI_R_SIT (movement imagination of sit-stand vs. rest while sitting) and MI_STD_SIT vs. MI_R_STD (movement imagination of stand-sit vs. rest while standing). To assess robustness, we evaluated performance across varying EEG segment lengths using the TCANet model. For MI_SIT_STD vs. MI_R_SIT, TCANet network achieved its best accuracy of $72.06 \pm 7.87\%$ with the 2-second segment, while the best F1-score and AUC of $70.47 \pm 13.13\%$ and 0.8222 ± 0.0899, respectively, were obtained with the 3-second segment. Moreover, the optimal training and inference times of 300.23 ± 42.52 and 0.10 ± 0.00 seconds, respectively, were achieved with the 1-second segment. For MI_STD_SIT vs. MI_R_STD, TCANet achieved the best accuracy of $73.60 \pm 9.33\%$ and F1-score of $72.12 \pm 14.28\%$ with the 3-second segment, while the AUC of 0.8389 ± 0.0969, optimal training of 312.73 ± 35.05 seconds, and inference times of 0.09 ± 0.00 seconds were obtained with the 5-second segment.

In addition, the confusion matrices for ME- and MI-based classification on the proposed dataset using the TCANet model are presented in Figure 6.

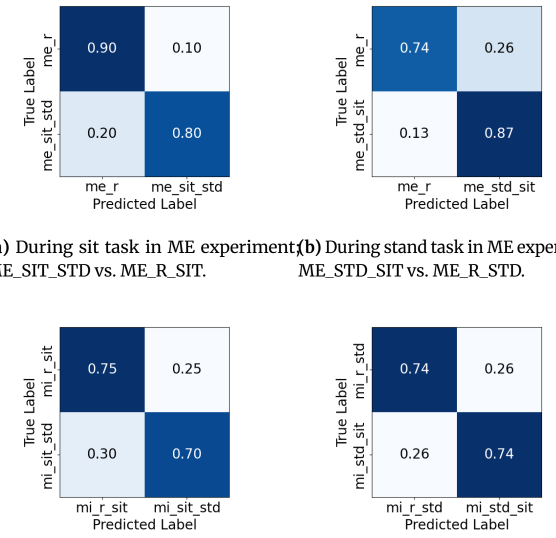

(a) During sit task in ME experiment; ME_SIT_STD vs. ME_R_SIT.

(b) During stand task in ME experiment; ME_STD_SIT vs. ME_R_STD.

(c) During sit task in MI experiment; MI_SIT_STD vs. MI_R_SIT.

(d) During stand task in MI experiment; MI_STD_SIT vs. MI_R_STD.

Figure 6. Confusion matrices of the proposed dataset for motor execution (ME) and motor imagery (MI) tasks using TCANet model.

Qualitative Analysis

This study also performed qualitative analyses of the recorded EEG signals to confirm the correctness and enhance the explainability of the obtained data. In the ME activity, we focus on analyzing the EEG signals in terms of movement-related cortical potentials (MRCPs), spontaneous potentials generated during person-generated movement [4, 20]. Figure 7 shows the grand average of EEG signals during ME activity for both sit and stand tasks across all subjects. The visualizations are obtained from the region of interest of 9 electrodes around the motor cortex area, in accordance with [21], including FC1, FCz, FC2, C1, Cz, C2, CP1, CPz, CP2, as shown in Figure 7a. These nine-electrode channels were selected based on their correspondence to the scalp projection of the lower-limb (foot) representation within the primary motor cortex (M1) area, consistent with the established somatotopic organization along the precentral gyrus [22]. This anatomical relevance is further supported by the topographic distribution illustrated in [23]. During the sit task (sit-stand, or ME_SIT_STD), it is observed from Figure 7b that, comparing to rest while sitting (ME_R_SIT), Bereitschaftspotential (BP, also known as readiness potential) exhibits slow negative EEG about 0.8 seconds (-0.8 to 0 s) before the actual onset of movement (designated by dotted gray vertical line) where the peak negativity lies around 0 s. Movement-monitoring potential (MMP), a component of MRCPs that reflects brain activity after the physical execution of a voluntary movement, relates to the brain's monitoring of the movement precision and control, lasting about 1 second after the movement onset.

On the other hand, for the stand task (stand-sit, or ME_STD_SIT), it is observed from Figure 7c that, compared to the rest while standing (ME_R_STD), the BP potential starts around 0.8 seconds (-0.8 s) before the actual onset of movement, gradually decreasing to the peak negativity around 0.25 seconds. The MMP lasts about 1 second after the onset of physical movement.

A small latency in the negative peak of MRCP signals is observed in ME stand-sit task (ME_STD_SIT), compared to ME sit-stand task (ME_SIT_STD). This could be related to the asymmetry of the neural preparatory process between these two tasks. The ME_SIT_STD task is considered to be a propulsive movement, requiring a rapid generation of force against gravity and inertia. This task demands a strong and immediate motor output which may requires a more decisive and rapid cortical preparation. Conversely, the ME_STD_SIT task involves a controlled descent, which requires precise and continuous modulation of muscle activity to decelerate the body and ensure a smooth, stable sitting. This control might involve a more prolonged preparatory phase, potentially delaying the MRCP negative peak [20].

For the MI activity, we analyze EEG signals in the time–frequency domain, i.e., how spectral components evolve over time. Figure 8 presents topographical maps of spectral power in the δ (1-4 Hz), θ (4-8 Hz), α (8-13 Hz), and β (13-30 Hz) bands, visualized for each second across a 5-second window. In terms of ERD/ERS, we observe pronounced event-related desynchronization (ERD)—a decrease in the power of an EEG rhythm—in frontal and central regions during the transition periods of both sit and stand motor-imagery tasks. This ERD is evident in the δ, θ, and α bands and is particularly strong in the β band, consistent with increased cor-

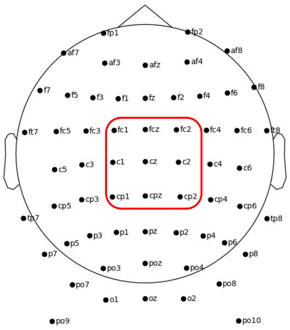

(a) Nine-channel electrode coordinates, drawn with red rectangle, within the motor cortex region of interest (ROI).

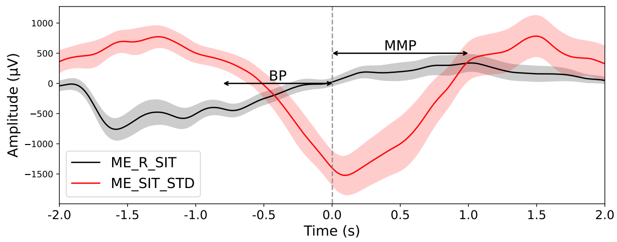

(b) During sit task in ME experiment; ME_SIT_STD vs. ME_R_SIT.

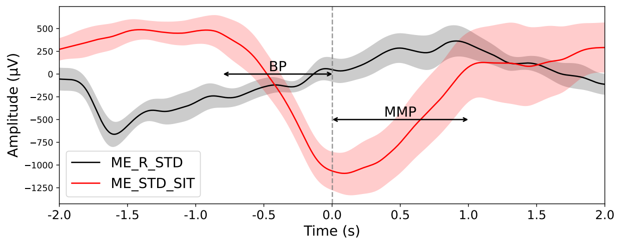

(c) During stand task in ME experiment; ME_STD_SIT vs. ME_R_STD).

Figure 7. Grand-average EEG activity (with standard error) of nine motor cortex electrodes during ME experiment.

tical activation and sensorimotor information processing during movement-related transitions.

On the other hand, we observe strong event-related synchronization (ERS)—an increase in the power of an EEG rhythm—primarily in the parietal region during resting (non-movement) periods, especially in the β band. In the δ, θ, and α bands, pronounced ERS is also evident in the parietal and frontal regions during resting. These observations are consistent with the findings reported by [24].

Discussion

This study presents and evaluates the performance of an EEG-based dataset, focusing on neural activation patterns of the sitting and standing conditions during MI and ME. As EEG has high variance across individuals [15], to support a variety of future applications on motor tasks, we train, validate, and test on a cross-subject (or subject-independent) basis.

In the ME activity, we present the classification performance for EEG signals obtained during the transition and resting periods. Since the classification focuses on training the model to learn MRCP features, we compare the models' performance using 1-second and 2-second EEG segments prior to movement onset for training and testing. From the results shown in Table 3, we see that the results of the 2-second EEG segment from all three models give the best classification performance and training time. Considering ME_SIT_STD vs. ME_R_SIT, although the 1-second EEG segment before the movement onset gives acceptable performances for at least 77.93% on accuracy when classified with TCANet, using the 2-second EEG segment provides significantly improved performance up to at least 81.15%, with around 193 seconds of training time. This is related to the ability of the classifier to learn a longer window of BP components. Compared to the resting EEG segments, we observe from Figure 7 that longer segments clearly show the BP components of ME activity related to the cortical excitability and readiness for the movement [25, 26].

In the MI activity, we present the classification performance for EEG signals obtained during the transition and resting periods. This classification task focuses on training the model to learn time-frequency features. We compare the models' performance across 1-second, 2-second, 3-second, 4-second, and 5-second EEG segments, starting from the visual stimulus onset, for both training and testing. From the results shown in Table 3, we see that the results of the 2-second EEG segments generally give the optimal performance in terms of classification performance and training time. For MI_SIT_STD vs. MI_R_SIT (sit-stand motor imagery vs. sit-rest), using the 2-second EEG segment after stimulus onset generally gives the optimal performance: 72.06% accuracy and 308.52 seconds of training time with TCANet. Similarly, for MI_STD_SIT vs. MI_R_STD (stand-sit motor imagery vs. stand-rest) tasks, although the best performance was obtained with the 3-second EEG segment, considering a balance of trade-offs on the segment length, computation time, and a little performance gain, the 1-second MI EEG segment seemed to be a good option to use. The reported training time is 313.94 seconds with 73.25% accuracy. These results are in accordance with the results reported by [27, 28] on lower-limb motor imagery classification.

To support this, Figure 8 shows topographical map visualizations for the grand average of spectral power in δ, θ, α, and β rhythms on 7-second MI EEG intervals (2 seconds of fixation and visual stimulus and 5 seconds after the visual stimulus onset). For both MI_SIT_STD vs. MI_R_SIT and MI_STD_SIT vs. MI_R_STD conditions, the differences in EEG distributions can be observed from the figure at 2 seconds after the onset of the visual stimulus. These visualizations correspond to the classification results previously reported in Table 3, ensuring the optimal selection of EEG segment lengths.

Compared to the existing studies on lower-limb-movement MI EEG datasets, our study targets transitional movement dynamics in sitting and standing movement initiation tasks. The goal of our study is to enable the exploration of neural activation patterns related to movement for supporting the development of BCI algorithms for mobility assistance and neural rehabilitation. To support this, our study includes high-density EEG recordings and tasks from ME and MI activity, enabling investigation of neural dynamics during movement transitions.

Although the highest mean classification accuracies for lower-limb MI EEG during sit-stand tasks have been reported at up to 88.51% in offline analysis and 96.56% in online analysis [5], the validation approach employed in that study raises concerns regarding practical applicability. Specifically, the researchers developed individual machine learning models tailored to each subject (within-subject or subject-dependent classification), rather than training a generalized model on a group of subjects and evaluating it on unseen subjects (cross-subject or subject-independent classification). This reliance on subject-dependent models may limit the applicability of such systems in real-world medical applications. In contrast, our study adopts a subject-independent validation framework to enhance generalizability across users. While this approach is inherently more challenging due to high inter-subject variability in EEG signals, it better reflects the conditions of real-world deployment.

Limitations

While this study provides a foundational characterization of movement-related neural signatures, it is important to acknowl-

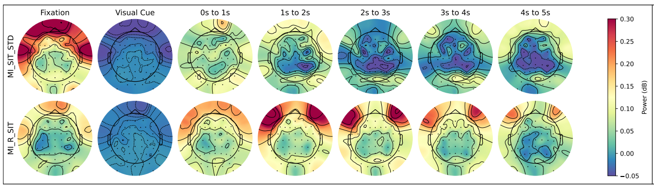

(a) MI_SIT_STD vs. MI_R_SIT in δ rhythm.

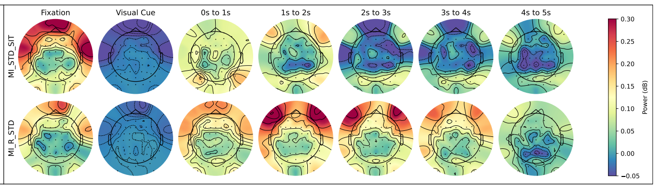

(b) MI_STD_SIT vs. MI_R_STD in δ rhythm.

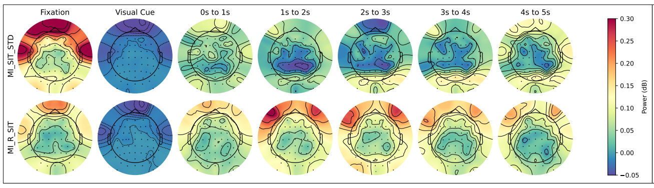

(c) MI_SIT_STD vs. MI_R_SIT in θ rhythm.

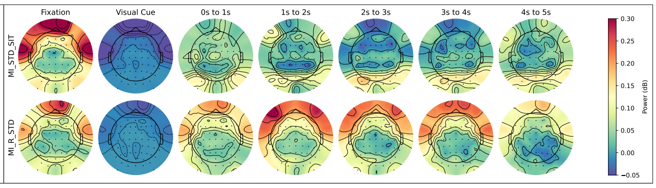

(d) MI_STD_SIT vs. MI_R_STD in θ rhythm.

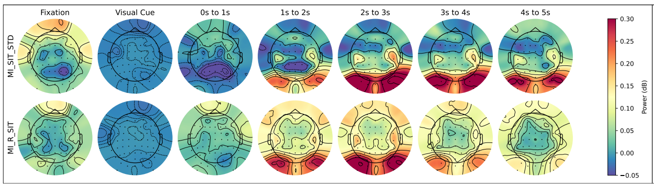

(e) MI_SIT_STD vs. MI_R_SIT in α rhythm.

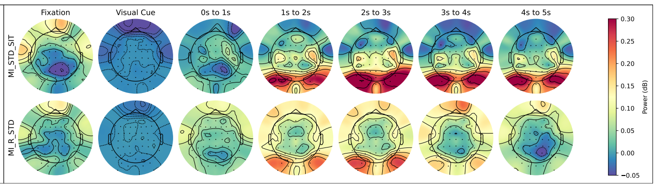

(f) MI_STD_SIT vs. MI_R_STD in α rhythm.

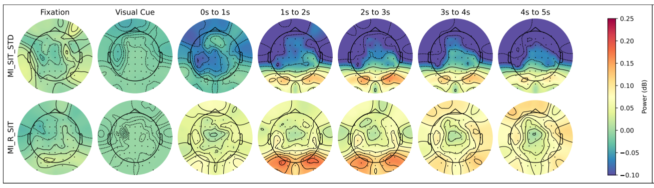

(g) MI_SIT_STD vs. MI_R_SIT in β rhythm.

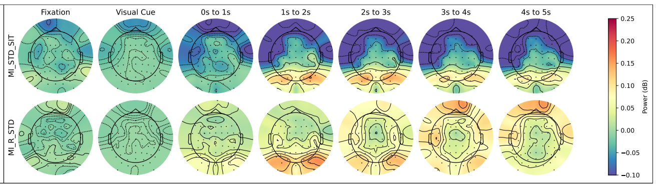

(h) MI_STD_SIT vs. MI_R_STD in β rhythm.

Figure 8. Topographical map visualizations for grand average of spectral power in δ (1-4 Hz), θ (4-8 Hz), α (8-13 Hz), and β (13-30 Hz) rhythms, calculated from the Morlet wavelet across 7 seconds (2 seconds of fixation and visual cue/visual stimulus and 5 seconds window after the visual stimulus onset) during various motor imagery (MI) activities. This includes MI_SIT_STD (motor imagery during sit to stand, or standing up from sitting on a chair) vs. MI_R_SIT (resting while sitting on a chair) and MI_STD_SIT (motor imagery during stand to sit, or sitting on a chair from standing up) vs. MI_R_STD (resting while standing).

edge that this dataset is obtained from healthy young adults, which presents a significant limitation for direct clinical translation, particularly for patients such as stroke survivors. Research indicates that movement-related neural dynamics—specifically the amplitude and latency of Movement-Related Cortical Potentials (MRCPs) as well as Event-Related Desynchronization/Synchronization (ERD/ERS) patterns—undergo substantial changes due to both aging and neurological impairment [29, 30, 31, 32]. These physiological differences create a domain shift, where models trained on healthy cohorts may fail to generalize effectively when applied to clinical cohorts for rehabilitation purposes [33, 34].

To successfully bridge the gap between these experimental findings and practical clinical applications, several mitigation strategies are required. Effective translation will depend on incorporating specialized knowledge of EEG characteristics related to lower-limb motor tasks unique to impaired populations into model design. Furthermore, technical approaches such as transfer learning, feature alignment, meta-learning, fine-tuning with condition-specific data, and applying domain adaptation will be essential to account for the altered signal distributions in clinical groups. Finally, future work may require external validation in cohorts aligned with specific rehabilitation scenarios to ensure these models remain robust and reliable in a real-world context.

Although this study employs deep learning algorithms for conducting experiments for dataset validation, this dataset is intended to be applicable for both conventional machine learning and deep learning-based lower-limb motor task classification methods. As training the deep learning model requires a large sample set size, data augmentation techniques on EEG signals in motor imagery tasks, such as those studies by [35, 36, 37], may be recommended to mitigate data scarcity concerns, providing methodological flexibility for future investigation.

Conclusion

This study introduces a novel EEG dataset focused on sitting and standing transitions during both motor execution (ME) and motor imagery (MI) tasks. The data, including 60 EEG channels, EOG, and EMG, were obtained from 22 participants who conducted two data recording sessions focused on lower-limb movements. To ensure the usability of the proposed dataset, we have evaluated its performance using three baseline BCI classification algorithms–CTNet, EEGNet, and TCANet–on two tasks–sit-stand vs. sit-rest and stand-sit vs. stand-rest. In addition, we evaluate performance across various EEG segment lengths to suggest further BCI applications. This dataset lays a solid foundation for the development and benchmarking of future BCI algorithms, offering researchers a valuable resource for better understanding and enhancing the performance of MI-based BCIs.

Abbreviations

EC: eyes-closed resting state; EO: eyes-opened resting state; ERD: event-related desynchronization; ERS: event-related synchronization; ERSP: event-related spectral perturbation; ME: motor execution; ME_R: resting during motor execution; ME_R_SIT: sit-resting during motor execution; ME_R_STD: stand-resting during motor execution; ME_SIT_STD: executing standing up from sitting; ME_STD_SIT: executing sitting down from standing; MI: motor imagery; MI_R: resting during motor imagery; MI_R_SIT: sit-resting during motor imagery; MI_R_STD: stand-resting during motor imagery; MI_SIT_STD: imagining standing up from sitting; MI_SIT_SIT: imagining sitting while sitting; MI_STD_SIT: imagining sitting down from standing; MI_STD_STD: imagining standing while standing; MRCP: movement-related cortical potential

Acknowledgment

We acknowledge the following funding and collaboration support from PTT Public Company Limited, The SCB Public Company Limited, Suranaree University of Technology (SUT), Thailand Science Research and Innovation (TSRI), and National Science, Research and Innovation Fund (NSRF), Thailand. This research has received funding support from the National Science, Research and Innovation Fund (NSRF) via the Program Management Unit for Human Resources & Institutional Development, Research and Innovation [grant number B13F680099].

Funding

National Science, Research and Innovation Fund (NSRF) Thailand, NRIIS Number: 195618 and 179275, G. Bhakdisongkhram. National Science, Research and Innovation Fund (NSRF) via the Program Management Unit for Human Resources & Institutional Development, Research and Innovation Thailand, Grant Number: B13F680099, T. Wilaiprasitporn, Thailand Science Research and Innovation (TSRI), Grant No.: FRB690039/0457, T. Wilaiprasitporn.

Data Availability

The raw and processed EEG data supporting the findings of this study are openly available in the Zenodo repository [38] and [39], respectively. The DOME-ML (Data, Optimisation, Model, Evaluation in Machine Learning) annotations in this study have been deposited in the DOME-ML Registry and can be accessed with the persistent identifier wnrszixlqm [40].

Availability of Source Code and Requirements

Project name: eeg_sit_stand
Project homepage: https://github.com/b5510546671/eeg_sit_stand
Operating system: Platform independent (tested on Unix-based systems)
Programming language: Python (version 3.8.0)
Other requirements:

- numpy (v2.0.2)
- scipy (v1.13.1)
- scikit-learn (v1.6.1)
- torch (v2.6.0)
- braindecode (v1.3.2)
- mne (v1.8.0)

License: Apache License 2.0

References

1. Arpaia P, Esposito A, Natalizio A, Parvis M. How to successfully classify EEG in motor imagery BCI: a metrological analysis of the state of the art. Journal of Neural Engineering 2022 jun;19(3):031002.
2. Bulea TC, Prasad S, Kilicarslan A, Contreras-Vidal JL. Sitting and standing intention can be decoded from scalp EEG recorded prior to movement execution. Frontiers in Neuroscience 2014;8.
3. Jeong JH, Kwak NS, Guan C, Lee SW. Decoding Movement-Related Cortical Potentials Based on Subject-Dependent and Section-Wise Spectral Filtering. IEEE Transactions on Neural Systems and Rehabilitation Engineering 2020;28(3):687–698.
4. Chaisaen R, Autthasan P, Mingchinda N, Leelaarporn P, Kunaseth N, Tammajarung S, et al. Decoding EEG Rhythms During Action Observation, Motor Imagery, and Execution for Standing and Sitting. IEEE Sensors Journal 2020;20(22):13776–13786.
5. Triana-Guzman N, Orjuela-Cañon AD, Jutinico AL, Mendoza-Montoya O, Antelis JM. Decoding EEG rhythms offline and online during motor imagery for standing and sitting based on a brain-computer interface. Frontiers in Neuroinformatics 2022 Sep;16.
6. Singh B, Natsume K. Readiness potential reflects the intention of sit-to-stand movement. Cognitive Neurodynamics 2023;17(3):605–620.
7. Autthasan P, Chaisaen R, Phan H, Vos MD, Wilaiprasitporn T. MixNet: Joining Force of Classical and Modern Approaches Toward the Comprehensive Pipeline in Motor Imagery EEG Classification. IEEE Internet of Things Journal 2024;11(17):28539–28554.
8. Autthasan P, Chaisaen R, Sudhawiyangkul T, Rangpong P, Kiatthaveephong S, Dilokthanakul N, et al. MIN2Net: End-to-End Multi-Task Learning for Subject-Independent Motor Imagery EEG Classification. IEEE Transactions on Biomedical Engineering 2022;69(6):2105–2118.
9. Gramfort A, Luessi M, Larson E, Engemann DA, Strohmeier D, Brodbeck C, et al. MEG and EEG Data Analysis with MNE-Python. Frontiers in Neuroscience 2013;7(267):1–13.
10. Kongwudhikunakorn S, Kiatthaveephong S, Thanontip K, Leelaarporn P, Piriyajitakonkij M, Charoenpattarawut T, et al. A Pilot Study on Visually Stimulated Cognitive Tasks for EEG-Based Dementia Recognition. IEEE Transactions on Instrumentation and Measurement 2021;70:1–10.
11. Rathee D, Raza H, Prasad G, Cecotti H. Current Source Density Estimation Enhances the Performance of Motor-Imagery-Related Brain–Computer Interface. IEEE Transactions on Neural Systems and Rehabilitation Engineering 2017;25(12):2461–2471.
12. Zhao W, Jiang X, Zhang B, Xiao S, Weng S. CTNet: a convolutional transformer network for EEG-based motor imagery classification. Scientific Reports 2024 Aug;14(1).
13. Lawhern VJ, Solon AJ, Waytowich NR, Gordon SM, Hung CP, Lance BJ. EEGNet: a compact convolutional neural network for EEG-based brain–computer interfaces. Journal of neural engineering 2018;15(5):056013.
14. Zhao W, Lu H, Zhang B, Zheng X, Wang W, Zhou H. TCANet: a temporal convolutional attention network for motor imagery EEG decoding. Cognitive Neurodynamics 2025 Jun;19(1).
15. Kongwudhikunakorn S, Ponwitayarat W, Kiatthaveephong S, Polpakdee W, Yagi T, Senanarong V, et al. EEGMeNet: End-to-End Multitask Neural Network for Brain-Based Mental Workload Classification. IEEE Internet of Things Journal 2025;12(20):42573–42589.
16. Musgrave K, Belongie SJ, Lim SN. PyTorch Metric Learning. ArXiv 2020;abs/2008.09164.
17. Pedregosa F, Varoquaux G, Gramfort A, Michel V, Thirion B, Grisel O, et al. Scikit-learn: Machine Learning in Python. Jour-

nal of Machine Learning Research 2011;12(85):2825–2830.
18. Karimi F, Kofman J, Mrachacz-Kersting N, Farina D, Jiang N. Detection of Movement Related Cortical Potentials from EEG Using Constrained ICA for Brain-Computer Interface Applications. Frontiers in Neuroscience 2017 Jun;11.
19. Li C, Guan H, Huang Z, Chen W, Li J, Zhang S. Improving Movement-Related Cortical Potential Detection at the EEG Source Domain. In: 2021 10th International IEEE/EMBS Conference on Neural Engineering (NER); 2021. p. 214–217.
20. Olsen S, Alder G, Williams M, Chambers S, Jochumsen M, Signal N, et al. Electroencephalographic Recording of the Movement-Related Cortical Potential in Ecologically Valid Movements: A Scoping Review. Frontiers in Neuroscience 2021 Sep;15.
21. Peng X, Liu J, Huang Y, Mao Y, Li D. Classification of lower limb motor imagery based on iterative EEG source localization and feature fusion. Neural Computing and Applications 2022 Jan;35(19):13711–13724.
22. Gordon EM, Chauvin RJ, Van AN, Rajesh A, Nielsen A, Newbold DJ, et al. A somato-cognitive action network alternates with effector regions in motor cortex. Nature 2023 Apr;617(7960):351–359.
23. Liu Y, Gui Z, Yan D, Wang Z, Gao R, Han N, et al. Lower limb motor imagery EEG dataset based on the multi-paradigm and longitudinal-training of stroke patients. Scientific Data 2025 Feb;12(1).
24. Jeon Y, Nam CS, Kim YJ, Whang MC. Event-related (De)synchronization (ERD/ERS) during motor imagery tasks: Implications for brain–computer interfaces. International Journal of Industrial Ergonomics 2011 Sep;41(5):428–436.
25. Di Russo F, Berchicci M, Bozzacchi C, Perri RL, Pitzalis S, Spinelli D. Beyond the "Bereitschaftspotential": Action preparation behind cognitive functions. Neuroscience and Biobehavioral Reviews 2017 Jul;78:57–81.
26. Ravi A, Wolfe P, Tung J, Jiang N. Signal Characteristics, Motor Cortex Engagement, and Classification Performance of Combined Action Observation, Motor Imagery and SSMVEP (CAMS) BCI. IEEE Transactions on Neural Systems and Rehabilitation Engineering 2025;33:1004–1013.
27. Sun Z, Mu A, Qian Y, Sun X. Bridging minds and limbs: novel hybrid deep learning approach for low-cost EEG-based lower limb motor imagery classification. Biomedical Signal Processing and Control 2026 Feb;112:108528.
28. Wang W, Shi B, Wang D, Wang J, Liu G. Enhanced lower-limb motor imagery by kinesthetic illusion. Frontiers in Neuroscience 2023 Jun;17.
29. Hirano D, Wada M, Kimura N, Jinnai D, Goto Y, Taniguchi T. Effects of divided attention on movement-related cortical potential in community-dwelling elderly adults: A preliminary study. Heliyon 2024 Jul;10(13).
30. Sailer A, Dichgans J, Gerloff C. The influence of normal aging on the cortical processing of a simple motor task. Neurology 2000 Oct;55(7):979–985.
31. Phang CR, Su KH, Cheng YY, Chen CH, Ko LW. Time synchronization between parietal–frontocentral connectivity with MRCP and gait in post-stroke bipedal tasks. Journal of NeuroEngineering and Rehabilitation 2024 Jun;21(1).
32. Vatinno AA, Simpson A, Ramakrishnan V, Bonilha HS, Bonilha L, Seo NJ. The Prognostic Utility of Electroencephalography in Stroke Recovery: A Systematic Review and Meta-Analysis. Neurorehabilitation and Neural Repair 2022 Mar;36(4–5):255–268.
33. Yang H, Wan J, Jin Y, Yu X, Fang Y. EEG- and EMG-Driven Poststroke Rehabilitation: A Review. IEEE Sensors Journal 2022 Dec;22(24):23649–23660.
34. Pirasteh A, Shamseini Ghiyasvand M, Pouladian M. EEG-based brain-computer interface methods with the aim of rehabilitating advanced stage ALS patients. Disability and Rehabilitation: Assistive Technology 2024 Feb;19(8):3183–3193.
35. Zhang Z, Duan F, Solé-Casals J, Dinarès-Ferran J, Cichocki A, Yang Z, et al. A Novel Deep Learning Approach With Data Augmentation to Classify Motor Imagery Signals. IEEE Access 2019;7:15945–15954.
36. George O, Smith R, Madiraju P, Yahyasoltani N, Ahamed SI. Data augmentation strategies for EEG-based motor imagery decoding. Heliyon 2022 Aug;8(8):e10240.
37. Rommel C, Paillard J, Moreau T, Gramfort A. Data augmentation for learning predictive models on EEG: a systematic comparison. Journal of Neural Engineering 2022 Nov;19(6):066020.
38. Leelakittisin B, Kongwudhikunakorn S, Kiatthaveephong S, Polpakdee W, Chaisaen R, Manoonpong P, et al., EEG-based dataset explicitly targets the transitions between sitting and standing for exploring neural activation patterns in motor imagery and execution. Zenodo; 2025. https://doi.org/10.5281/zenodo.20348444.
39. Leelakittisin B, Kongwudhikunakorn S, Kiatthaveephong S, Polpakdee W, Chaisaen R, Manoonpong P, et al., EEG-based dataset explicitly targets the transitions between sitting and standing for exploring neural activation patterns in motor imagery and execution [preprocessed dataset]. Zenodo; 2025. https://doi.org/10.5281/zenodo.20348464.
40. Leelakittisin B, Kongwudhikunakorn S, Kiatthaveephong S, Polpakdee W, Chaisaen R, Manoonpong P, et al., EEG-Based Dataset Explicitly Targets the Transitions between Sitting and Standing for Exploring Neural Activation Patterns in Motor Imagery and Execution. [DOME-ML Annotations]. DOME-ML Registry; 2026. https://registry.dome-ml.org/review/wnrszixlqm.

Dear Prof. Hongfang Zhang, Editor of GigaScience Journal,

We would like to express our sincere gratitude for the opportunity to revise and resubmit our manuscript entitled: “EEG-Based Dataset Explicitly Targeting the Transitions between Sitting and Standing for Exploring Neural Activation Patterns in Motor Imagery and Execution”
(Paper: GIGA-D-25-00472).

We appreciate your precious time and effort in reviewing our manuscript, providing fruitful comments, and motivating us to carefully revise our manuscript.

We appreciate all two reviewer’s valuable comments and suggestions. Their constructive comments have helped us to substantially improve the quality, clarity, and depth of our work. In response, we have carefully revised our manuscript in every section–from Abstract to Conclusion, including the references–has been refined to address the reviewers’ concerns. The parts that we have modified are indicated in red text, as in the attached revised manuscript in PDF file.

In summary, key improvements include:

- Beyond EEGNet, we conducted additional experiments using CTNet and TCANet on both MI and ME datasets, and added confusion matrix analysis from the best-performing model (TCANet) to provide deeper insight into classification error patterns.
- We carefully revised to clearly differentiate our dataset by highlighting our unique focus on transitional movement dynamics and movement initiation.
- To support reproducibility, we publicly released our full source code on GitHub.
- We improved the visual clarity by clearly labeling the ROI region, expanding the topographic maps to include fixation and cue-stage brain activity, and adding before-and-after ICA visualizations to better illustrate the effectiveness of our artifact removal procedure.

We have also included a detailed point-by-point response letter addressing each reviewer’s comments and outlining the corresponding revisions in the manuscript. We believe that these extensive revisions have significantly strengthened our work, and we respectfully resubmit the revised manuscript for

your kind consideration. We hope the revised version meets the standards of your esteemed journal and look forward to your favorable response.

We would like to correspond to the reviewers' obliging comments, as detailed below. Due to our template being in a LaTex format (not MS Word), we kindly ask you to check the revised manuscript in the attached PDF version.

Again, we highly appreciate your valuable time and effort for reviewing this study.

Thank you very much,
Best regards,

Theerawit Wilaiprasitporn, Ph.D.
Associate Professor of Computer Engineering at the School of Information Science
and Technology (IST), Vidyasirimedhi Institute of Science & Technology (VISTEC),
Thailand
Associate Editor, IEEE Sensors Journal
Associate Editor, IEEE Internet of Things Journal

Contact: +66982736144

Original Comments

Reviewer: 1

The study presents a novel contribution, but several important issues must be addressed to strengthen the manuscript:

Dear Reviewer 1,

Thank you very much for your time and effort in reviewing our manuscript. We sincerely appreciate your insightful and constructive comments, which have been invaluable in guiding us to improve the quality and clarity of our work. We have carefully considered all your suggestions and have thoroughly revised the manuscript accordingly. Below, we provide detailed responses to each of your comments and explain the corresponding modifications made to the manuscript.

Reviewer Comment 1.1

1. The research aims to support rehabilitation for clinical populations (e.g., stroke survivors), yet the dataset is derived from 22 healthy young adults (22-28 years). Neural dynamics like MRCPs likely differ significantly in older or impaired populations. The Discussion must explicitly acknowledge this major limitation and discuss the challenges of translating models trained on this data to target clinical groups.

Author's Responses:
We sincerely thank the reviewer for highlighting these important issues and for the valuable suggestions. We acknowledge that our dataset comprises healthy young adults only which may limit direct translation of neural dynamics to clinical populations. In the revised manuscript, we have explicitly discussed this limitation, techniques, and challenges of translating the models trained on EEG recordings obtained from healthy participants to the impaired populations. Our suggestion for further alleviating this issue includes performing domain adaptation, transfer learning, feature alignment, and meta-learning on EEG recordings, as presented in section Limitations (as captured below).

Limitations

While this study provides a foundational characterization of movement-related neural signatures, it is important to acknowl-

edge that this dataset is obtained from healthy young adults, which presents a significant limitation for direct clinical translation, particularly for patients such as stroke survivors. Research indicates that movement-related neural dynamics—specifically the amplitude and latency of Movement-Related Cortical Potentials (MRCPs) as well as Event-Related Desynchronization/Synchronization (ERD/ERS) patterns—undergo substantial changes due to both aging and neurological impairment [29, 30, 31, 32]. These physiological differences create a domain shift, where models trained on healthy cohorts may fail to generalize effectively when applied to clinical cohorts for rehabilitation purposes [33, 34].

To successfully bridge the gap between these experimental findings and practical clinical applications, several mitigation strategies are required. Effective translation will depend on incorporating specialized knowledge of EEG characteristics related to lower-limb motor tasks unique to impaired populations into model design. Furthermore, technical approaches such as transfer learning, feature alignment, meta-learning, fine-tuning with condition-specific data, and applying domain adaptation will be essential to account for the altered signal distributions in clinical groups. Finally, future work may require external validation in cohorts aligned with specific rehabilitation scenarios to ensure these models remain robust and reliable in a real-world context.

Although this study employs deep learning algorithms for conducting experiments for dataset validation, this dataset is intended to be applicable for both conventional machine learning and deep learning-based lower-limb motor task classification methods. As training the deep learning model requires a large sample set size, data augmentation techniques on EEG signals in motor imagery tasks, such as those studies by [35, 36, 37], may be recommended to mitigate data scarcity concerns, providing methodological flexibility for future investigation.

Reviewer Comment 1.2

2. With only 40 ME or MI trials per session, the total per-class samples per subject are relatively low for deep learning. Please add a discussion on the potential impact of sample size on DL-based model performance and possible mitigation strategies.

Author's Responses:

We sincerely thank the reviewer for highlighting these important issues and for the valuable suggestions. This point has been discussed in the section Limitations of the revised manuscript. Although each session contains 40 ME or MI trials, each subject completed two sessions, resulting in 80 trials per subject. In this study, model training was conducted on a cross-subject basis, yielding a total of 1,760 trials for training and evaluation. This aggregation substantially increases the effective sample size compared with single-session or subject-specific settings, partially alleviating concerns regarding limited trials per subject. Furthermore, similar per-subject trial numbers have been reported in recent EEG datasets [1].

Nevertheless, we acknowledged in section Limitations (as captured below) that the dataset size may still pose challenges for deep learning models. In practice, several strategies may help mitigate this issue, such as data augmentation (e.g., sliding-window segmentation) and other techniques commonly used in EEG-based motor imagery studies [2–3], which may be explored in future work. Importantly, the dataset is not restricted to deep learning approaches and can also support conventional machine learning methods, providing flexibility for future methodological exploration.

Although this study employs deep learning algorithms for conducting experiments for dataset validation, this dataset is intended to be applicable for both conventional machine learning and deep learning-based lower-limb motor task classification methods. As

training the deep learning model requires a large sample set size, data augmentation techniques on EEG signals in motor imagery tasks, such as those studies by [35, 36, 37], may be recommended to mitigate data scarcity concerns, providing methodological flexibility for future investigation.

Reviewer Comment 1.3

3. The paper notes that MI_SIT_SIT and MI_STD_STD conditions were recorded but not used in the analysis. Please briefly justify their inclusion and subsequent exclusion for transparency.

Author's Responses:

We sincerely thank the reviewer for highlighting this point and for the valuable suggestion. We have revised the manuscript in section Data Collection Protocol, as captured below.

To address your concern, MI_SIT_SIT and MI_STD_STD are static imagery conditions in which participants imagine a posture that already matches their current physical state—sitting while seated or standing while standing—and therefore do not involve any directional motor transition. Consequently, their neural signatures are expected to closely resemble those of the resting conditions, making them difficult to distinguish meaningfully and unsuitable for the transition-focused classification framework adopted in this study. These conditions were originally included in the data collection protocol to broadly explore posture-related motor imagery paradigms for potential BCI applications. However, since the primary objective of the present study is to characterize the phase-specific neural dynamics of actual postural transitions relative to resting, these static conditions fall outside the intended scope and were excluded from the final analyses.

Nevertheless, their recorded data remain publicly available to support future investigations. Characterizing neural activity differences between static posture motor imagery and resting is itself a meaningful and underexplored research problem. For instance, detecting a user's static postural intention could serve as an early BCI trigger prior to movement execution, which is particularly relevant for severely impaired patients, such as those with advanced ALS or late-stage stroke, who may be unable to perform physical transitions at all. We therefore regard this as a promising direction for subsequent research.

MI during sit was divided into two nonconsecutive rounds, each consisting of 20 pseudorandomized trials (10 for MI_SIT_STD and 10 for MI_SIT_SIT). In the remaining part of this study, the data obtained during MI_SIT_SIT is not used, as they do not involve directional motor transitions and are expected to produce neural signatures closely resembling resting states. However, the data are provided to enable further exploration in potential studies, such as the detection of a user's static postural intentions.

MI during stand was divided into two nonconsecutive rounds, each consisting of 20 pseudorandomized trials (10 for MI_STD_SIT and 10 for MI_STD_STD). In the remaining part of this study, the data obtained during MI_STD_STD is not used, as they do not involve directional motor transitions and are expected to produce neural signatures closely resembling resting states. However, the data are provided to enable further exploration in potential studies, such as the detection of a user's static postural intentions.

Reviewer Comment 1.4

4. The claims of being the "first" public dataset for sit-to-stand/stand-to-sit transitions and that "none offers EEG recordings..." are incorrect and must be revised. A similar public dataset exists (Triana-Guzman et al., 2024, OpenNeuro ds005342). The authors must correct these statements and discuss how their dataset differs from this existing resource.
Triana-Guzman N, Orjuela-Cañon AD, Jutinico AL, Mendoza-Montoya O and Antelis JM (2024). EEG data offline and online during motor imagery for standing and sitting. OpenNeuro Dataset ds005342. doi: doi:10.18112/openneuro.ds005342.v1.0.3

Author's Responses:

We sincerely thank the reviewer for highlighting these important issues and for the valuable suggestions. While the dataset by Triana-Guzman et al. 2024 involves sit-to-stand and stand-to-sit–related tasks, the two datasets differ in their objectives. Specifically, Triana-Guzman et al. 2024 primarily focuses on MI classification of completed standing and sitting states, whereas our dataset is designed to investigate transitional movement dynamics—particularly the neural signatures associated with movement initiation—which are critical for rehabilitation-oriented BCI applications.

Furthermore, our dataset offers high-density EEG recordings of up to 60 channels and includes both motor execution (ME) and motor imagery (MI) tasks. These features enable researchers to explore neural dynamics during movement transitions with higher spatial resolution, and facilitate studies on ME–MI relationships as well as model development for transition-based BCI control. This design additionally supports investigation of the neural mechanisms underlying movement initiation and transitional control, which are essential considerations for developing rehabilitation-oriented BCI systems targeting functional movements such as sit-to-stand transitions.

To address the reviewer's concern, we have carefully reviewed the study by Triana-Guzman et al. 2024 and revised the manuscript accordingly to better clarify the positioning of our dataset relative to theirs. The differences between the two studies are now explicitly discussed and summarized in both the Introduction (top image) and Discussion (bottom image) sections of the revised manuscript, as shown in the captured images below.

Despite its potential, sit-stand and stand-sit transitions remain underrepresented in MI-BCI research. Current publicly available MI-BCI EEG datasets predominantly focus on traditional hand and foot movements due to their well-defined and distinctive neural patterns [7, 8]. While these datasets have supported the development of advanced EEG-based classification algorithms, none offers EEG recordings of sit-stand or stand-sit motor imagery. The lower-limb movement datasets have been well presented in the study by Triana-Guzman et.al [5]; however, their main objective lies on classifying completed movement task. In practical BCI scenarios, distinguishing transition-related neural activity from initial resting states and detecting early movement intention are critical challenges. This gap underscores the need for dedicated datasets focusing on these transitions to better capture lower-limb dynamics and expand the application of MI-BCIs in rehabilitation.

Compared to the existing studies on lower-limb-movement motor-imagery EEG dataset, our study targets transitional movement dynamics in sitting and standing movement initiation tasks. The goal of our study is to enable the exploration of neural activation patterns related to the movement for supporting the development of BCI algorithms for mobility assistance and neural rehabilitation. To support this, our study includes high-density EEG recordings and both motor execution (ME) and motor imagery (MI) tasks, enabling investigation of neural dynamics during movement transitions.

Reviewer Comment 1.5

5. Describing EEGNet as the "state of the art" is misleading; it is a well-established baseline model. To better frame the work, please briefly discuss some recent advanced architectures that combine EEGNet with temporal modeling (such as EEG-TCNet, CTNet, TCANet), which may achieve higher decoding accuracy.

Ingolfsson T M, Hersche M, Wang X, et al. EEG-TCNet: An accurate temporal convolutional network for embedded motor-imagery brain-machine interfaces[C]//2020 IEEE International Conference on Systems, Man, and Cybernetics (SMC). IEEE, 2020: 2958-2965.

Zhao W, Jiang X, Zhang B, et al. CTNet: a convolutional transformer network for EEG-based motor imagery classification[J]. Scientific reports, 2024, 14(1): 20237.

Zhao W, Lu H, Zhang B, et al. TCANet: a temporal convolutional attention network for motor imagery EEG decoding[J]. Cognitive Neurodynamics, 2025, 19(1): 91.

Author's Responses:

We sincerely thank the reviewer for highlighting these important issues and for the valuable suggestions. To address your concern, we have further conducted similar experiments using CTNet and TCANet models on both MI and ME datasets, in which the experiment results are reported in Table 3 (captured below). The detailed description of both models are presented in section Method (as captured below). To avoid the misleading issue, we have carefully revised the statement, refraining from stating that the EEGNet is the "state-of-the-art" model.

CTNet [12]: A convolutional transformer network designed for EEG-based classification of motor imagery. The first layer of CTNet employs convolutional module for local and spatial EEG features extraction, while Transformer encoder module is employed in the subsequent layer to learn global dependencies in high-level EEG features. CTNet shows remarkable decoding accuracies for both subject-specific and cross-subject evaluations. Our implementation uses Python with *PyTorch* [16], *scikit-learn* [17], and *mne* [9] libraries. In this study, the parameters settings are set as follows: number of attention heads $heads = 4$, embedding size $emb\_size = 40$, depth $d = 6$, kernel size $kernel = 64$. The optimal batch size b and the learning rate lr are set as $b = 8$ and $lr = 1 \times 10^{-3}$, respectively. The model is trained subjected to cross-entropy loss for 200 epochs, while the early stopping is triggered when the loss does not improve for 10 consecutive epochs. To ensure unbiased learning, we apply five-fold cross-validation technique while training and validating for an optimal model's performance.

EEGNet-8,2: EEGNet [13] effectively learns spatiotemporal EEG features through a compact convolutional neural network. It employs depthwise and separable convolutions to enhance feature learning while reducing trainable parameters, improving efficiency without sacrificing classification performance in EEG-based BCI tasks. Our implementation uses *Python* with *PyTorch* and *scikit-learn*. The parameters settings of EEGNet-8,2 are set as follows: number of filters in the first layer $F1 = 8$, depth parameter $D = 2$, kernel size $C1 = 200$, number of classes $n_{class} = 2$, dropout rate $r_{dropout} = 0.5$. The optimal batch size b and learning rate lr are set to be $b = 8$ and $lr = 1 \times 10^{-3}$, respectively. The model is trained subjected to cross-entropy loss for 200 epochs, while the early stopping is triggered when the loss does not decrease for 10 consecutive epochs. To ensure unbiased learning, we apply five-fold cross-validation when training and validating EEGNet for an optimal model's performance.

TCANet [14]: A multi-scale temporal convolutional attention network designed for EEG-based classification of motor imagery task. The first layer adopts multi-scale convolutional module to extract local spatiotemporal features across different temporal resolutions. Subsequently, the temporal convolutional module combines and compresses these multi-scale features. Finally, the multi-head self-attention mechanism learns global dependencies features in the EEG. Our implementation uses Python with *PyTorch*, *scikit-learn*, and *mne* libraries. In this study, the parameters settings of TCANet are set as follows: filter size $f_1 = 16$, pooling size $pooling = 56$, number of attention heads $heads = 2$, depth $d = 6$, dropout rate $r_{dropout} = 0.25$. The optimal batch size b and learning rate lr are set to be $b = 8$ and $lr = 1 \times 10^{-3}$, respectively. The model is trained subjected to cross-entropy loss for 200 epochs, while the early stopping is triggered when the loss does not improve for 10 consecutive epochs. To ensure unbiased learning, we apply five-fold cross-validation technique while training and validating for an optimal model's performance.

Table 3. Classification performance of three baseline methods (CTNet, EEGNet, TCANet), along with training T_{train} and inference T_{infer} times (in seconds) per one fold, on the proposed dataset for motor execution (ME) and motor imagery (MI) tasks using different EEG segment lengths (Mean ± SD).

Experiment	Task	Segment Length (s)	Accuracy ↑	F1-score ↑	AUC ↑	T_{train} (s) ↓	T_{infer} (s) ↓
			**Method: CTNet**				
ME	ME_SIT_STD vs ME_R_SIT	1	77.35 ± 8.78*	74.37 ± 12.34*	0.8570 ± 0.0939*	**413.00 ± 37.31**	**0.39 ± 0.04**
		2	**81.25 ± 8.30**	**80.34 ± 9.64**	**0.8996 ± 0.0766**	610.73 ± 488.64*	0.59 ± 0.63
	ME_STD_SIT vs ME_R_STD	1	77.60 ± 9.57*	74.95 ± 13.32	0.8643 ± 0.0894*	**410.64 ± 41.86**	**0.39 ± 0.07**
		2	**79.89 ± 9.52**	**78.98 ± 11.00**	**0.8912 ± 0.0903**	602.05 ± 493.91*	0.60 ± 0.58*
MI	MI_SIT_STD vs MI_R_SIT	1	71.41 ± 8.48	68.31 ± 13.53	0.7889 ± 0.0986	**160.61 ± 9.20**	**0.09 ± 0.00**
		2	**71.93 ± 8.30**	**70.19 ± 12.01**	0.8053 ± 0.0903	164.70 ± 11.78	0.09 ± 0.00*
		3	71.23 ± 8.81	69.74 ± 13.63	0.8082 ± 0.0987	241.26 ± 16.86*	0.23 ± 0.02*
		4	70.31 ± 9.80	68.29 ± 16.37	0.7986 ± 0.1073	258.53 ± 19.47*	0.24 ± 0.02*
		5	70.77 ± 8.45	68.69 ± 13.98	**0.8136 ± 0.0921**	2566.27 ± 134.43*	3.89 ± 0.46*
	MI_STD_SIT vs MI_R_STD	1	72.79 ± 8.23	70.24 ± 12.43	0.8073 ± 0.0829	**159.37 ± 8.15**	**0.09 ± 0.00**
		2	72.80 ± 8.32	70.48 ± 13.51	0.8157 ± 0.0874	162.18 ± 9.88	0.09 ± 0.00*
		3	**73.08 ± 10.15**	**70.56 ± 17.15**	**0.8240 ± 0.0914**	240.23 ± 13.91*	0.23 ± 0.03*
		4	71.95 ± 11.23	69.24 ± 18.39	0.8209 ± 0.1045	256.81 ± 20.99*	0.23 ± 0.02*
		5	71.55 ± 11.49	67.35 ± 18.94	0.8125 ± 0.1073	2454.36 ± 139.27*	3.63 ± 0.51*
			**Method: EEGNet**				
ME	ME_SIT_STD vs ME_R_SIT	1	75.91 ± 9.38*	70.87 ± 14.84*	0.8210 ± 0.1091*	70.01 ± 9.33*	0.06 ± 0.01
		2	**79.85 ± 7.99**	**77.60 ± 10.47**	**0.8844 ± 0.0751**	**63.37 ± 7.63**	**0.06 ± 0.01**
	ME_STD_SIT vs ME_R_STD	1	76.02 ± 9.68*	71.68 ± 15.62*	0.8302 ± 0.0984*	**61.40 ± 5.53**	0.06 ± 0.01
		2	**79.68 ± 8.80**	**77.93 ± 11.88**	**0.8866 ± 0.0836**	63.22 ± 6.65	**0.06 ± 0.01**
MI	MI_SIT_STD vs MI_R_SIT	1	69.16 ± 8.62	67.48 ± 13.28	0.7677 ± 0.0981	**86.96 ± 12.95**	0.08 ± 0.01
		2	**69.58 ± 8.86**	69.69 ± 11.15	0.7994 ± 0.0973	92.32 ± 15.79	**0.07 ± 0.01**
		3	68.98 ± 9.56	**70.70 ± 11.84**	**0.8045 ± 0.0975**	155.97 ± 27.96*	0.14 ± 0.03*
		4	67.85 ± 10.24	69.58 ± 12.91	0.7992 ± 0.1016	168.59 ± 32.24*	0.13 ± 0.04*
		5	67.39 ± 10.25	69.16 ± 14.02	0.7985 ± 0.1031	167.96 ± 25.68*	0.14 ± 0.03*
	MI_STD_SIT vs MI_R_STD	1	71.37 ± 7.44	68.73 ± 13.24	0.7872 ± 0.0969	**83.03 ± 11.12**	0.07 ± 0.01
		2	71.27 ± 8.33	**70.15 ± 13.16**	0.8022 ± 0.1001	95.65 ± 13.74*	**0.07 ± 0.01**
		3	70.88 ± 10.82	68.77 ± 19.01	0.8080 ± 0.1204	124.96 ± 24.34*	0.08 ± 0.03
		4	**71.42 ± 11.13**	68.58 ± 20.98	**0.8149 ± 0.1129**	149.74 ± 19.01*	0.07 ± 0.01
		5	70.63 ± 11.54	67.51 ± 21.92	0.8049 ± 0.1215	148.15 ± 14.33*	0.07 ± 0.02
			**Method: TCANet**				
ME	ME_SIT_STD vs ME_R_SIT	1	77.93 ± 8.95	74.44 ± 12.70	0.8532 ± 0.0987*	230.21 ± 31.27*	**0.09 ± 0.00**
		2	**81.15 ± 8.36**	**79.81 ± 10.38**	**0.8958 ± 0.0700**	**192.74 ± 19.02**	0.10 ± 0.00*
	ME_STD_SIT vs ME_R_STD	1	78.69 ± 9.88	74.84 ± 15.55	0.8628 ± 0.0885	237.31 ± 25.10*	**0.09 ± 0.01**
		2	**80.86 ± 8.83**	**79.65 ± 11.44**	**0.8993 ± 0.0770**	**216.13 ± 24.89**	0.10 ± 0.00*
MI	MI_SIT_STD vs MI_R_SIT	1	70.48 ± 9.61	67.88 ± 15.24	0.7776 ± 0.1059*	**300.23 ± 42.52**	**0.10 ± 0.00**
		2	**72.06 ± 7.87**	70.24 ± 11.43	0.8090 ± 0.0893	308.52 ± 35.18	0.10 ± 0.01*
		3	71.66 ± 8.83	**70.47 ± 13.13**	**0.8222 ± 0.0899**	446.30 ± 54.60*	0.24 ± 0.02*
		4	70.77 ± 9.73	69.40 ± 15.25	0.8162 ± 0.0908	416.40 ± 49.81*	0.25 ± 0.02*
		5	69.98 ± 9.11	68.70 ± 15.46	0.8102 ± 0.0906*	397.43 ± 69.92*	0.24 ± 0.04*
	MI_STD_SIT vs MI_R_STD	1	73.25 ± 8.34	71.78 ± 11.84	0.8113 ± 0.0940	313.94 ± 39.21	**0.09 ± 0.00**
		2	72.92 ± 7.58	71.88 ± 10.91	0.8162 ± 0.0885	**312.73 ± 35.05**	0.10 ± 0.00*
		3	**73.60 ± 9.33**	**72.12 ± 14.28**	0.8354 ± 0.0897	439.43 ± 91.45*	0.22 ± 0.06*
		4	73.21 ± 10.94	70.98 ± 17.59	0.8388 ± 0.0948	447.48 ± 57.71*	0.24 ± 0.02*
		5	73.53 ± 10.52	71.23 ± 17.18	**0.8389 ± 0.0969**	419.95 ± 50.11*	0.24 ± 0.02*

Note: * indicates statistically significant difference using a *t*-test ($p \leq 0.05$) compared to the best-performing setting, highlighted in bold. Segment length refers to the EEG window duration in seconds, extracted relative to stimulus onset (before onset for ME, after onset for MI).

Abbreviations: ME_SIT_STD, executing standing up from sitting; ME_R_SIT, rest while sitting; ME_STD_SIT, executing sitting down from standing; ME_R_STD, rest while standing; MI_SIT_STD, imagining standing up from sitting; MI_R_SIT, rest while sitting; MI_STD_SIT, imagining sitting down from standing; MI_R_STD, rest while standing; T_{train}, training time; T_{test}, inference time.

Reviewer Comment 1.6

6. Providing only accuracy/F1 scores is insufficient. Adding a confusion matrix analysis would greatly help evaluate data quality. Future researchers would benefit from knowing the specific error patterns.

Author's Responses:
We sincerely thank the reviewer for insightful comments and highlighting these important issues. In addition to the existing reported accuracies, F1-scores, and AUC, training time, and inference time, we have reported the results from the best performing model (TCANet) in terms of confusion matrices, as shown in Figure 6. By including this confusion matrix may greatly be beneficial for the future researchers on deep diving the classification results and extending analysis.

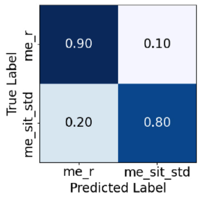

(a) During sit task in ME experiment; ME_SIT_STD vs. ME_R_SIT.

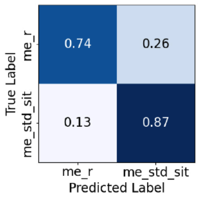

(b) During stand task in ME experiment; ME_STD_SIT vs. ME_R_STD.

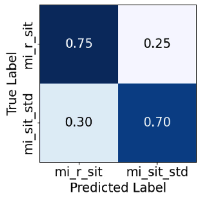

(c) During sit task in MI experiment; MI_SIT_STD vs. MI_R_SIT.

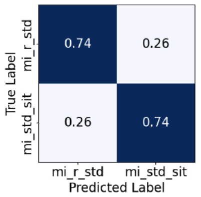

(d) During stand task in MI experiment; MI_STD_SIT vs. MI_R_STD.

Figure 6. Confusion matrices of the proposed dataset for motor execution (ME) and motor imagery (MI) tasks using TCANet model.

Reviewer Comment 1.7

7. To ensure reproducibility and maximize the dataset's utility, I strongly recommend publicly releasing the complete code (e.g., on GitHub). This should include:
Full data loading/preprocessing scripts (for both MRCP and time-frequency pipelines).
The exact EEGNet implementation used, including LOSOCV split, training loop, and evaluation.
Scripts to reproduce the key results (Table 3, Figures 5/6).

Author's Responses:
We sincerely thank the reviewer for addressing this issue. We totally agree with your comments to publish the full source code for enhancing reproducibility and maximize the dataset's utility. The source code has been published to the Github repository (https://github.com/b5510546671/eeg_sit_stand) and has been explicitly stated in the Source Code Availability statement.

Source Code Availability Statement

The source code supporting the experiments of this article is available in the Github repository (https://github.com/b5510546671/eeg_sit_stand).

Reviewer Comment 1.8

8. Minor Typo: On Page 8, "dropout rate dropout = 0.5" should be "dropout rate = 0.5".

Author's Responses:
We sincerely thank the reviewer for addressing this issue. We have fixed the typo errors and thoroughly checked the whole manuscript to avoid any overlooked mistakes.

head self-attention mechanism learns global dependencies features in the EEG. Our implementation uses Python with *PyTorch*, *scikit-learn*, and *mne* libraries. In this study, the parameters settings of TCANet are set as follows: filter size $f_1 = 16$, pooling size $pooling = 56$, number of attention heads $heads = 2$, depth $d = 6$, dropout rate $r_{dropout} = 0.25$. The optimal batch size b and learning rate lr are set to be $b = 8$ and $lr = 1 \times 10^{-3}$, respectively. The model is trained subjected to cross-entropy loss for 200 epochs, while the early stopping is triggered when the loss does not improve for 10 consecutive epochs. To ensure unbiased learning, we apply five-fold cross-validation technique while training and validating for an optimal model's performance.

Reviewer #2

There are few MI EEG datasets on sit-to-stand transitions, which makes this study valuable for BCI research. The data collected from 20 subjects, covering both MI and ME paradigms, is particularly meaningful. Overall, the paper is logically structured and the data quality appears good.

Dear Reviewer 2,

Thank you very much for your time and effort in reviewing our manuscript. We sincerely appreciate your insightful and constructive comments, which have been invaluable in guiding us to improve the quality and clarity of our work. We have carefully considered all your suggestions and have thoroughly revised the manuscript accordingly. Below, we provide detailed responses to each of your comments and explain the corresponding modifications made to the manuscript.

Reviewer Comment 2.1

1. Considering that sit-to-stand ME involves significant body posture changes, it is likely to introduce much greater motion artifacts compared to upper limb movements such as those of the hands. Although ME typically elicits stronger EEG responses than MI, I doubt that ICA assisted only by EMG and EOG can fully remove the influence of motion artifacts. Please support this with relevant references or provide a more detailed explanation of your data processing procedure.

Author's Responses:
We sincerely thank the reviewer for addressing this significant issue. In the revised manuscript, to better support our explanation on data preprocessing steps, we have visualized the EEG signals observed before and after performing independent component analysis (ICA) for ocular- and muscular-related artifactual components removal, as shown in Figure 5 (as captured below). Three visualizations are presented: the top visualization compares EEG signals from all channels, the middle compares EEG global field power from all channels, and the bottom compares the average of EEG signals across all channels. It is observed that the data after cleaning (shown in black) exhibits lower fluctuation and fewer artifactual components compared to before cleaning (shown in red), which exhibits high-frequency components appearing as sharp burst-like waveforms. To enhance clarity of the manuscript, we have also revised the Data Preprocessing section accordingly (as captured below).

It should also be noted that in this study, the ICA-cleaned EEG signals are used for visualization purposes only. Since the current deep learning baseline models are designed in an end-to-end style, the EEG time series used to train these models are preprocessed by time-locked segmentation and bandpass filtering only.

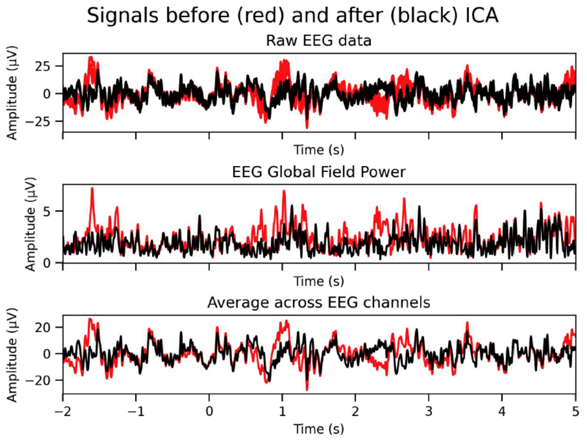

Figure 5. Comparison of EEG signals before (shown in red line) and after (shown in black line) independent component analysis (ICA). The top visualization compares the EEG signals from all channels. The middle visualization compares the EEG global field power from all channels. The bottom visualization compares the average of EEG signals across all channels.

Data Preprocessing

This study's EEG recordings were preprocessed to analyze brain activity during EC, EO, MI, and ME activities. Specifically, movement-related cortical potential (MRCP) features were examined, predominantly associated with ME activities. Conversely, time-frequency power distribution features were analyzed for EC, EO, and MI activities, as recommended and validated in previous studies [4, 10]. Due to these differences, the preprocessing pipelines for these activities varied slightly, as described in detail below. All preprocessing steps were conducted using the *MNE-Python* library [9].

Preprocessing Steps for Movement-Related Cortical Potential (MRCP) Analysis

For MRCP feature extraction in ME-based classification, the following preprocessing steps were applied:

The 60-channel EEG recordings were first filtered with a second-order Butterworth bandpass filter with a 0.2 to 3 Hz cutoff frequency. Subsequently, the signals were downsampled to 250 Hz. To remove artifacts, independent component analysis (ICA) was performed to decompose independent components (ICs) and eliminate artifacts using *mne.preprocessing.ICA* in *MNE-Python* library. To assist the ICA process, the eye-related artifacts were eliminated using recorded EOG signals, while the muscle-related artifacts were eliminated using the function *find_bads_muscle()*. Following, signals from identified bad channels were removed and interpolated using data from neighboring electrodes. To mitigate volume conduction effects and enhance spatial resolution, the Current Source Density (CSD) transformation was applied [11]. Finally, the preprocessed signals were segmented into 4-second epochs ranging from -2 to 2 seconds relative to an onset of EMG trigger event (indicating $T = 0$). (Note: The EMG data from session #2 of subject #20 is unavailable. To resolve this issue, specifically for this circumstance, the 4-second epochs were segmented using the onset of the event triggers #11 and #12 obtained from channel number #63.) Furthermore, we

excluded those trials contaminated by noise and amplitude spikes with a trial rejection based on peak-to-peak (PTP) amplitude calculation of EEG signals, as those trials with large PTP amplitudes indicate the presence of artifacts. Any trials with the PTP amplitudes exceeding this 95th percentile threshold are automatically flagged and rejected from further analysis. On average, 36 trials remained after rejection. Thus, the first 36 trials were selected for further analysis.

For ME-based classification, each trial was labeled according to a participant's physical movement, either ME activities or resting task (ME_R). The ME resting trials are alternately separated into ME_R_SIT (rest while sitting) and ME_R_STD (rest while standing), respectively. Start from index 0, the even-numbered trials are labeled as ME_R_STD, while the odd-numbered trials are labeled as ME_R_SIT. Similarly, trials labeled as ME_SIT_STD and ME_STD_SIT correspond to a participant performing a sit-stand and stand-sit transition, respectively.

Preprocessing Steps for Time-Frequency Distribution Analysis

Preprocessing for MI-based classification, including EC and EO, was similar to that used for MRCP analysis, with the following modifications:

Instead of using a second-order Butterworth bandpass filter (0.2–3 Hz), a sixth-order Butterworth bandpass filter with 1–40 Hz cutoff frequencies was applied. The processed signals were also segmented into trials ranging from -2 to 5 seconds relative to the event trigger onset.

In contrast to the ME task, the MI task did not involve any actual movement; therefore, no EMG onset was observed. The preprocessed signals were segmented into epochs relative to the onset of the event trigger #21 and #32, as described in Table 2. Each trial spanned 7 seconds (2 seconds before and 5 seconds after the event trigger onset). In order to minimize the influence of ongoing background activity, a 2-second segment before the event trigger was used for baseline correction, leaving a 5-second segment for further analysis. We excluded those trials contaminated with noise by a threshold-based trial rejection using PTP amplitude calculation, similar to the steps for MRCP analysis. On average, 36 trials remained after rejection and were selected for further analysis. For MI-based classification, trials were labeled according to participants' current physical states. MI_R_SIT and MI_R_STD corresponded to a participant resting while sitting and standing, respectively. Similarly, trials labeled as MI_SIT_STD and MI_STD_SIT corresponded to the participant imagining sit-stand and stand-sit transitions, respectively.

It should be noted that the EEG data preprocessed with ICA and CSD were utilized for visualization, whereas data without these preprocessing steps were used for classification with CTNet, EEGNet, and TCANet models. To confirm the quality of the signals cleaned from the ICA, we compare the signals before and after the cleaning, as shown in Figure 5. From the figure, we observed that the data after cleaning (shown in black) exhibits lower fluctuation and artifactual components compared to the data before cleaning (shown in red), which exhibits high-frequency components and appears as sharp burst-like waveforms. In addition, our deep learning-based classification experiments were conducted on segmented, filtered EEG time-series data, based on the assumption that the state-of-the-art models are designed in an end-to-end style.

To further address the reviewer's concern regarding movement-related artifacts in sit-to-stand ME, we provide additional evidence from the grand average EEG activity shown in Figure 7 (as captured below). From the

ICA-assisted preprocessed EEG signals, the ME sit-to-stand (ME_SIT_STD) condition reveals a prominent Bereitschaftspotential (BP) component **right before any motor execution occurs**—clearly observable around **0.8 seconds prior to movement onset**—distinctly confirming that this preparatory neural signal genuinely precedes the act of moving, with peak negativity reached near time zero. Following onset, a movement-monitoring potential (MMP) persists for approximately 1 second. The presence of these well-characterized task-related neural signatures, low-amplitude slow cortical potentials highly susceptible to contamination, demonstrates that the ICA-based artifact removal was effective in suppressing motion-related interference while preserving meaningful EEG activity associated with the sit-to-stand movement, consistent with previous studies on MRCP detection during lower-limb motor tasks [4,5,6].

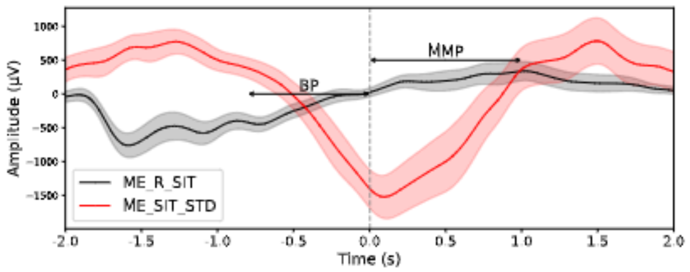

(b) During sit task in ME experiment; ME_SIT_STD vs. ME_R_SIT.

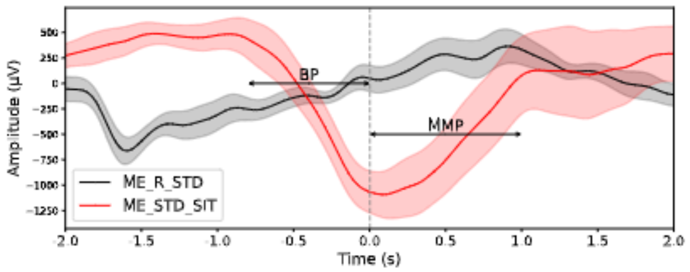

(c) During stand task in ME experiment; ME_STD_SIT vs. ME_R_STD).

Figure 7. Grand-average EEG activity (with standard error) of nine motor cortex electrodes during ME experiment.

Reviewer Comment 2.2

2. I understand that classification accuracy for lower limb MI is objectively relatively low. However, the MI classification accuracy reported in this paper still seems somewhat unsatisfactory. The authors could cite results from other lower limb MI studies, perform classification on publicly available lower limb MI datasets for comparison, or provide a separate explanation to better demonstrate the data quality of this study.

Author's Responses:

We appreciate the reviewer's constructive feedback. To address this concern, we expanded our evaluation beyond the existing EEGNet model by conducting additional experiments with CTNet and TCANet models. **The best classification accuracy for lower limb MI across these models was 73.60 ± 9.33%**, as reported in Table 3 (captured below).

Experiment	Task	Segment Length (s)	Accuracy ↑	F1-score ↑	AUC ↑	T_{train} (s) ↓	T_{infer} (s) ↓
			**Method: CTNet**				
ME	ME_SIT_STD vs ME_R_SIT	1	77.35 + 8.78*	74.37 + 12.34*	0.8570 + 0.0939*	**413.00 + 37.31**	**0.39 + 0.04**
		2	**81.25 + 8.30**	**80.34 + 9.64**	**0.8996 + 0.0766**	610.73 + 488.64*	0.59 + 0.63
	ME_STD_SIT vs ME_R_STD	1	77.60 + 9.57*	74.95 + 13.32	0.8643 + 0.0894*	**410.64 + 41.86**	**0.39 + 0.07**
		2	**79.89 + 9.52**	**78.98 + 11.00**	**0.8912 + 0.0903**	602.05 + 493.91*	0.60 + 0.58*
MI	MI_SIT_STD vs MI_R_SIT	1	71.41 + 8.48	68.31 + 13.53	0.7889 + 0.0986	**160.61 + 9.20**	**0.09 + 0.00**
		2	**71.93 + 8.30**	**70.19 + 12.01**	0.8053 + 0.0903	164.70 + 11.78	0.09 + 0.00*
		3	71.23 + 8.81	69.74 + 13.63	0.8082 + 0.0987	241.26 + 16.86*	0.23 + 0.02*
		4	70.31 + 9.80	68.29 + 16.37	0.7986 + 0.1073	258.53 + 19.47*	0.24 + 0.02*
		5	70.77 + 8.45	68.69 + 13.98	**0.8136 + 0.0921**	2566.27 + 134.43*	3.89 + 0.46*
	MI_STD_SIT vs MI_R_STD	1	72.79 + 8.23	70.24 + 12.43	0.8073 + 0.0829	**159.37 + 8.15**	**0.09 + 0.00**
		2	72.80 + 8.32	70.48 + 13.51	0.8157 + 0.0874	162.18 + 9.88	0.09 + 0.00*
		3	**73.08 + 10.15**	**70.56 + 17.15**	**0.8240 + 0.0914**	240.23 + 13.91*	0.23 + 0.03*
		4	71.95 + 11.23	69.24 + 18.39	0.8209 + 0.1045	256.81 + 20.99*	0.23 + 0.02*
		5	71.55 + 11.49	67.35 + 18.94	0.8125 + 0.1073	2454.36 + 139.27*	3.63 + 0.51*
			**Method: EEGNet**				
ME	ME_SIT_STD vs ME_R_SIT	1	75.91 + 9.38*	70.87 + 14.84*	0.8210 + 0.1091*	70.01 + 9.33*	0.06 + 0.01
		2	**79.85 + 7.99**	**77.60 + 10.47**	**0.8844 + 0.0751**	**63.37 + 7.63**	**0.06 + 0.01**
	ME_STD_SIT vs ME_R_STD	1	76.02 + 9.68*	71.68 + 15.62*	0.8302 + 0.0984*	**61.40 + 5.53**	0.06 + 0.01
		2	**79.68 + 8.80**	**77.93 + 11.88**	**0.8866 + 0.0836**	63.22 + 6.65	**0.06 + 0.01**
MI	MI_SIT_STD vs MI_R_SIT	1	69.16 + 8.62	67.48 + 13.28	0.7677 + 0.0981	**86.96 + 12.95**	0.08 + 0.01
		2	**69.58 + 8.86**	69.69 + 11.15	0.7994 + 0.0973	92.32 + 15.79	**0.07 + 0.01**
		3	68.98 + 9.56	**70.70 + 11.84**	**0.8045 + 0.0975**	155.97 + 27.96*	0.14 + 0.03*
		4	67.85 + 10.24	69.58 + 12.91	0.7992 + 0.1016	168.59 + 32.24*	0.13 + 0.04*
		5	67.39 + 10.25	69.16 + 14.02	0.7985 + 0.1031	167.96 + 25.68*	0.14 + 0.03*
	MI_STD_SIT vs MI_R_STD	1	71.37 + 7.44	68.73 + 13.24	0.7872 + 0.0969	**83.03 + 11.12**	0.07 + 0.01
		2	71.27 + 8.33	**70.15 + 13.16**	0.8022 + 0.1001	95.65 + 13.74*	**0.07 + 0.01**
		3	70.88 + 10.82	68.77 + 19.01	0.8080 + 0.1204	124.96 + 24.34*	0.08 + 0.03
		4	**71.42 + 11.13**	68.58 + 20.98	**0.8149 + 0.1129**	149.74 + 19.01*	0.07 + 0.01
		5	70.63 + 11.54	67.51 + 21.92	0.8049 + 0.1215	148.15 + 14.33*	0.07 + 0.02
			**Method: TCANet**				
ME	ME_SIT_STD vs ME_R_SIT	1	77.93 + 8.95	74.44 + 12.70	0.8532 + 0.0987*	230.21 + 31.27*	**0.09 + 0.00**
		2	**81.15 + 8.36**	**79.81 + 10.38**	**0.8958 + 0.0700**	**192.74 + 19.02**	0.10 + 0.00*
	ME_STD_SIT vs ME_R_STD	1	78.69 + 9.88	74.84 + 15.55	0.8628 + 0.0885	237.31 + 25.10*	**0.09 + 0.01**
		2	**80.86 + 8.83**	**79.65 + 11.44**	**0.8993 + 0.0770**	**216.13 + 24.89**	0.10 + 0.00*
MI	MI_SIT_STD vs MI_R_SIT	1	70.48 + 9.61	67.88 + 15.24	0.7776 + 0.1059*	**300.23 + 42.52**	**0.10 + 0.00**
		2	**72.06 + 7.87**	70.24 + 11.43	0.8090 + 0.0893	308.52 + 35.18	0.10 + 0.01*
		3	71.66 + 8.83	**70.47 + 13.13**	**0.8222 + 0.0899**	446.30 + 54.60*	0.24 + 0.02*
		4	70.77 + 9.73	69.40 + 15.25	0.8162 + 0.0908	416.40 + 49.81*	0.25 + 0.02*
		5	69.98 + 9.11	68.70 + 15.46	0.8102 + 0.0906*	397.43 + 69.92*	0.24 + 0.04*
	MI_STD_SIT vs MI_R_STD	1	73.25 + 8.34	71.78 + 11.84	0.8113 + 0.0940	313.94 + 39.21	**0.09 + 0.00**
		2	72.92 + 7.58	71.88 + 10.91	0.8162 + 0.0885	**312.73 + 35.05**	0.10 + 0.00*
		3	**73.60 + 9.33**	**72.12 + 14.28**	0.8354 + 0.0897	439.43 + 91.45*	0.22 + 0.06*
		4	73.21 + 10.94	70.98 + 17.59	0.8388 + 0.0948	447.48 + 57.71*	0.24 + 0.02*
		5	73.53 + 10.52	71.23 + 17.18	**0.8389 + 0.0969**	419.95 + 50.11*	0.24 + 0.02*

It is worth emphasizing that our study is designed to assess the robustness of the trained classifier to unseen subjects. In contrast to the majority of existing work, which focuses on subject-dependent (within-subject) classification and consequently reports higher accuracy scores, we adopt a subject-independent (cross-subject) evaluation framework—a more challenging yet clinically realistic setting. Our results show that TCANet exhibits strong tolerance to inter-subject EEG variability, achieving the highest classification accuracy of 73.60%. This demonstrates that our approach offers greater practical applicability compared to subject-dependent paradigms.

In the revised manuscript, a paragraph has been incorporated into the Discussion section to address the classification performance reported in a related study, as presented below.

Although the highest mean classification accuracies for lower-limb motor-imagery EEG during sit-stand tasks have been reported at up to 88.51% in offline analysis and 96.56% in online analysis [5], the validation approach employed in that study raises concerns regarding practical applicability. Specifically, the researchers developed individual machine learning models tailored to each subject (within-subject or subject-dependent classification), rather than training a generalized model on a group of subjects and evaluating it on unseen subjects (cross-subject or subject-independent classification). This reliance on subject-dependent models may limit the practicability of such systems in real-world medical applications. In contrast, our study adopts a subject-independent validation framework to enhance generalizability across users. While this approach is inherently more challenging due to high inter-subject variability in EEG signals, it better reflects the conditions of real-world deployment.

Reviewer Comment 2.3

3. 9-electrode ROI mentioned in the Qualitative Analysis actually corresponds to the lower limb-related motor cortex area. Please label this clearly and cite relevant papers.

Author's Responses:
We sincerely thank the reviewer for the valuable recommendation. The selected 9-electrode ROI corresponds to the motor cortex area associated with lower-limb representation. This selection is supported by EEG source localization results reported in [7]. To enhance clarification, in the revised version of the manuscript, we have revised the section Qualitative Analysis, clearly labeled this 9-electrode ROI region (as captured below), as shown in the section, and added the corresponding references to support further readings.

Qualitative Analysis
This study also performed qualitative analyses of the recorded EEG signals to confirm the correctness and enhance the explainability of the obtained data. In the ME activity, we focus on analyzing the EEG signals in terms of movement-related cortical potentials (MR-CPs), spontaneous potentials generated during person-generated movement [4, 20]. Figure 7 shows the grand average of EEG signals during ME activity for both sit and stand tasks across all subjects. The visualizations are obtained from the region of interest of 9 electrodes around the motor cortex area, in accordance with [21], including FC1, FCz, FC2, C1, Cz, C2, CP1, CPz, CP2, as shown in Figure 7a. These nine-electrode channels were selected based on their correspondence to the scalp projection of the lower-limb (foot) representation within the primary motor cortex (M1) area, consistent with the established somatotopic organization along the precentral gyrus [22]. This anatomical relevance is further supported by

the topographic distribution illustrated in [23]. During the sit task (sit-stand, or ME_SIT_STD), it is observed from Figure 7b that, comparing to rest while sitting (ME_R_SIT), Bereitschaftspotential (BP, also known as readiness potential) exhibits slow negative EEG about 0.8 seconds (-0.8 to 0 s) before the actual onset of movement (designated by dotted gray vertical line) where the peak negativity lies around 0 s. Movement-monitoring potential (MMP), a component of MRCPs that reflects brain activity after the physical execution of a voluntary movement, relates to the brain's monitoring of the movement precision and control, lasting about 1 second after the movement onset.

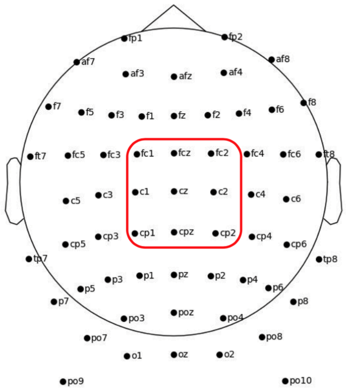

(a) Nine-channel electrode coordinates, drawn with red rectangle, within the motor cortex region of interest (ROI).

Reviewer Comment 2.4

4. Please add fixation and cue-stage topographic maps to Figure 6, and circle the ROI mentioned earlier in the Qualitative Analysis section.

Author's Responses:

We sincerely thank the reviewer for the valuable recommendation. In the revised manuscript, we have thoroughly modified the topographic maps by adding the brain activity during the 2-second period of fixation and cue-stage prior the motor tasks to Figure 8 (previously Figure 6, as captured below). From the figures, we observe strong ERD responses (power decreases) during visual cue observation, indicating active cortical processing during motor task preparation.

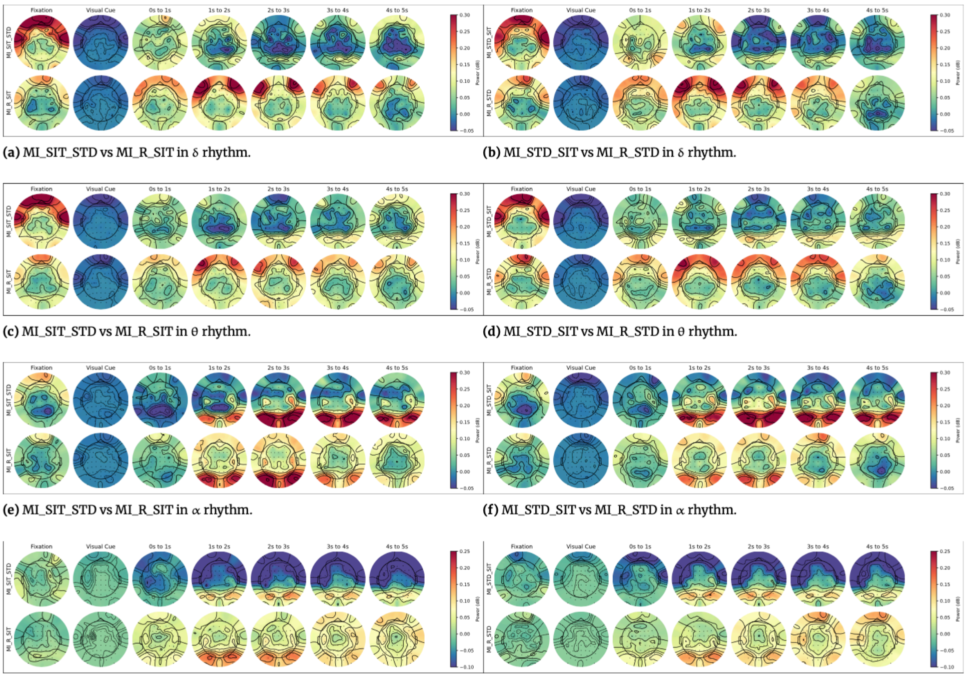

(a) MI_SIT_STD vs MI_R_SIT in δ rhythm. **(b)** MI_STD_SIT vs MI_R_STD in δ rhythm.

(c) MI_SIT_STD vs MI_R_SIT in θ rhythm. **(d)** MI_STD_SIT vs MI_R_STD in θ rhythm.

(e) MI_SIT_STD vs MI_R_SIT in α rhythm. **(f)** MI_STD_SIT vs MI_R_STD in α rhythm.

(g) MI_SIT_STD vs MI_R_SIT in β rhythm. **(h)** MI_STD_SIT vs MI_R_STD in β rhythm.

Figure 8. Topographical map visualizations for grand average of spectral power in δ (1-4 Hz), θ (4-8 Hz), α (8-13 Hz), and β (13-30 Hz) rhythms, calculated from the Morlet wavelet across 7 seconds (2 seconds of fixation and visual cue/visual stimulus and 5 seconds window after the visual stimulus onset) during various motor imagery (MI) activities. This includes MI_SIT_STD (motor imagery during sit to stand, or standing up from sitting on a chair) vs MI_R_SIT (resting while sitting on a chair) and MI_STD_SIT (motor imagery during stand to sit, or sitting on a chair from standing up) vs MI_R_STD (resting while standing).

To clarify and prevent any misunderstandings of the region of interest (ROI) mentioned in the manuscript, this indicates that the MRCP plot visualized in Figure 7 (captured below) was obtained from the EEG recordings of 9 electrodes around motor cortex area (ROI, highlighted with red square) which clearly shows the BP components of ME activity related to the readiness for the movement.

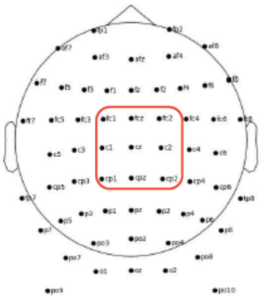

(a) Nine-channel electrode coordinates, drawn with red rectangle, within the motor cortex region of interest (ROI).

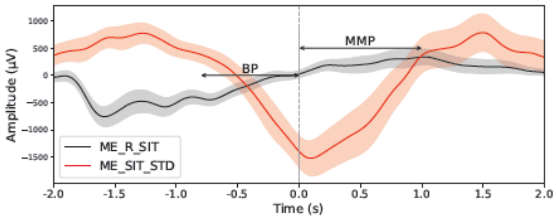

(b) During sit task in ME experiment; ME_SIT_STD vs. ME_R_SIT.

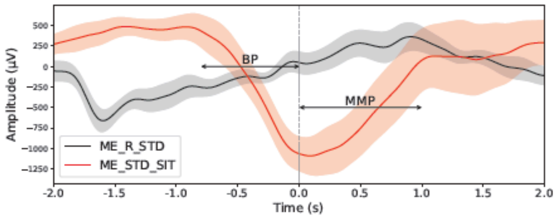

(c) During stand task in ME experiment; ME_STD_SIT vs. ME_R_STD).

Figure 7. Grand-average EEG activity (with standard error) of nine motor cortex electrodes during ME experiment.

In summary, Figure 8 presents the topographical distribution of EEG power in response to visual stimulus onset, while Figure 7 illustrates the distribution of averaged MRCP signals recorded from 9 electrodes over the motor cortex region during motor preparation preceding the visual stimulus.

References

[1] Liu, Y., Gui, Z., Yan, D., Wang, Z., Gao, R., Han, N., ... & Ming, D. (2025). Lower limb motor imagery EEG dataset based on the multi-paradigm and longitudinal-training of stroke patients. Scientific Data, 12(1), 314.
[2] Anjerani, M., Pedram, M. M., & Mirzarezaee, M. (2025). Data augmentation and feature extraction using deep learning for motor imagery EEG-based brain–computer interface classification. Neural Computing and Applications, 37(23), 19339-19369.
[3] George, O., Smith, R., Madiraju, P., Yahyasoltani, N., & Ahamed, S. I. (2022). Data augmentation strategies for EEG-based motor imagery decoding. Heliyon, 8(8).
[4] Chaisaen R, Autthasan P, Mingchinda N, Leelaarporn P, Kunaseth N, Tammajarung S, et al. (2020). Decoding EEG Rhythms During Action Observation, Motor Imagery, and Execution for Standing and Sitting. IEEE Sensors Journal, 20(22):13776–13786.
[5] Singh B, Natsume K. Readiness potential reflects the intention of sit-to-stand movement. (2023). Cognitive Neurodynamics;17(3):605–620.
[6] Karimi F, Kofman J, Mrachacz-Kersting N, Farina D, Jiang N. (2017). Detection of Movement Related Cortical Potentials from EEG Using Constrained ICA for Brain-Computer Interface Applications. Frontiers in Neuroscience, 11.
[7] Peng, X., Liu, J., Huang, Y., Mao, Y., & Li, D. (2023). Classification of lower limb motor imagery based on iterative EEG source localization and feature fusion. Neural Computing and Applications, 35(19), 13711-13724.
