# Supplementary material for: EEG-based dataset explicitly targets the transitions between sitting and standing for exploring neural activation patterns in motor imagery and execution
Source: Gigascience. 2026 May 29;15:giag065. doi: 10.1093/gigascience/giag065 (PMC13270978; doi:10.1093/gigascience/giag065)
Supplement: giag065_Reviewer_1_Report_original_submission — Reviewer 1 -- 12/16/2025 [file giag065_reviewer_1_report_original_submission.pdf]

Reviewer Report

Title: EEG-Based Dataset Explicitly Targeting the Transitions between Sitting and Standing for Exploring Neural Activation Patterns in Motor Imagery and Execution

Version: Original Submission **Date:** 12/16/2025

Reviewer name: Reviewer 1

Reviewer Comments to Author:

The study presents a novel contribution, but several important issues must be addressed to strengthen the manuscript:1. The research aims to support rehabilitation for clinical populations (e.g., stroke survivors), yet the dataset is derived from 22 healthy young adults (22-28 years). Neural dynamics like MRCPs likely differ significantly in older or impaired populations. The Discussion must explicitly acknowledge this major limitation and discuss the challenges of translating models trained on this data to target clinical groups.2. With only 40 ME or MI trials per session, the total per-class samples per subject are relatively low for deep learning. Please add a discussion on the potential impact of sample size on DL-based model performance and possible mitigation strategies.3. The paper notes that MI_SIT_SIT and MI_STD_STD conditions were recorded but not used in the analysis. Please briefly justify their inclusion and subsequent exclusion for transparency.4. The claims of being the "first" public dataset for sit-to-stand/stand-to-sit transitions and that "none offers EEG recordings..." are incorrect and must be revised. A similar public dataset exists (Triana-Guzman et al., 2024, OpenNeuro ds005342). The authors must correct these statements and discuss how their dataset differs from this existing resource.Triana-Guzman N, Orjuela-CaÃ±on AD, Jutinico AL, Mendoza-Montoya O and Antelis JM (2024). EEG data offline and online during motor imagery for standing and sitting. OpenNeuro Dataset ds005342. doi: doi:10.18112/openneuro.ds005342.v1.0.35. Describing EEGNet as the "state of the art" is misleading; it is a well-established baseline model. To better frame the work, please briefly discuss some recent advanced architectures that combine EEGNet with temporal modeling (such as EEG-TCNet, CTNet, TCANet), which may achieve higher decoding accuracy.Ingolfsson T M, Hersche M, Wang X, et al. EEG-TCNet: An accurate temporal convolutional network for embedded motor-imagery brain-machine interfaces[C]//2020 IEEE International Conference on Systems, Man, and Cybernetics (SMC). IEEE, 2020: 2958-2965.Zhao W, Jiang X, Zhang B, et al. CTNet: a convolutional transformer network for EEG-based motor imagery classification[J]. Scientific reports, 2024, 14(1): 20237.Zhao W, Lu H, Zhang B, et al. TCANet: a temporal convolutional attention network for motor imagery EEG decoding[J]. Cognitive Neurodynamics, 2025, 19(1): 91.6. Providing only accuracy/F1 scores is insufficient. Adding a confusion matrix analysis would greatly help evaluate data quality. Future researchers would benefit from knowing the specific error patterns.7. To ensure reproducibility and maximize the dataset's utility, I strongly recommend publicly releasing the complete code (e.g., on GitHub). This should include:Full data loading/preprocessing scripts (for both MRCP and time-frequency pipelines).The exact EEGNet implementation used, including LOSOCV split, training loop, and evaluation.Scripts to reproduce the key results (Table 3, Figures 5/6).8. Minor Typo: On Page 8, "dropout rate dropout = 0.5" should be "dropout rate = 0.5".

Level of Interest

Please indicate how interesting you found the manuscript: Choose an item.

Quality of Written English

Please indicate the quality of language in the manuscript: Choose an item.

Declaration of Competing Interests

Please complete a declaration of competing interests, considering the following questions:

- Have you in the past five years received reimbursements, fees, funding, or salary from an organisation that may in any way gain or lose financially from the publication of this manuscript, either now or in the future?
- Do you hold any stocks or shares in an organisation that may in any way gain or lose financially from the publication of this manuscript, either now or in the future?
- Do you hold or are you currently applying for any patents relating to the content of the manuscript?
- Have you received reimbursements, fees, funding, or salary from an organization that holds or has applied for patents relating to the content of the manuscript?
- Do you have any other financial competing interests?
- Do you have any non-financial competing interests in relation to this paper?

If you can answer no to all of the above, write 'I declare that I have no competing interests' below. If your reply is yes to any, please give details below.

I declare that I have no competing interests

I agree to the open peer review policy of the journal. I understand that my name will be included on my report to the authors and, if the manuscript is accepted for publication, my named report including any attachments I upload will be posted on the website along with the authors' responses. I agree for my report to be made available under an Open Access Creative Commons CC-BY license (http://creativecommons.org/licenses/by/4.0/). I understand that any comments which I do not wish to be included in my named report can be included as confidential comments to the editors, which will not be published.

Choose an item.

To further support our reviewers, we have joined with Publons, where you can gain additional credit to further highlight your hard work (see: https://publons.com/journal/530/gigascience). On publication of

this paper, your review will be automatically added to Publons, you can then choose whether or not to claim your Publons credit. I understand this statement.

Yes Choose an item.
