# Supplementary material for: EEG-based dataset explicitly targets the transitions between sitting and standing for exploring neural activation patterns in motor imagery and execution
Source: Gigascience. 2026 May 29;15:giag065. doi: 10.1093/gigascience/giag065 (PMC13270978; doi:10.1093/gigascience/giag065)
Supplement: giag065_Reviewer_2_Report_original_submission — Reviewer 2 -- 12/23/2025 [file giag065_reviewer_2_report_original_submission.pdf]

Reviewer Report

Title: EEG-Based Dataset Explicitly Targeting the Transitions between Sitting and Standing for Exploring Neural Activation Patterns in Motor Imagery and Execution

Version: Original Submission **Date:** 12/23/2025

Reviewer name: Reviewer 2

Reviewer Comments to Author:

There are few MI EEG datasets on sit-to-stand transitions, which makes this study valuable for BCI research. The data collected from 20 subjects, covering both MI and ME paradigms, is particularly meaningful. Overall, the paper is logically structured and the data quality appears good.1. Considering that sit-to-stand ME involves significant body posture changes, it is likely to introduce much greater motion artifacts compared to upper limb movements such as those of the hands. Although ME typically elicits stronger EEG responses than MI, I doubt that ICA assisted only by EMG and EOG can fully remove the influence of motion artifacts. Please support this with relevant references or provide a more detailed explanation of your data processing procedure.2. I understand that classification accuracy for lower limb MI is objectively relatively low. However, the MI classification accuracy reported in this paper still seems somewhat unsatisfactory. The authors could cite results from other lower limb MI studies, perform classification on publicly available lower limb MI datasets for comparison, or provide a separate explanation to better demonstrate the data quality of this study.3. 9-electrode ROI mentioned in the Qualitative Analysis actually corresponds to the lower limb-related motor cortex area. Please label this clearly and cite relevant papers.4. Please add fixation and cue-stage topographic maps to Figure 6, and circle the ROI mentioned earlier in the Qualitative Analysis section.

Level of Interest

Please indicate how interesting you found the manuscript: Choose an item.

Quality of Written English

Please indicate the quality of language in the manuscript: Choose an item.

Declaration of Competing Interests

Please complete a declaration of competing interests, considering the following questions:

- Have you in the past five years received reimbursements, fees, funding, or salary from an organisation that may in any way gain or lose financially from the publication of this manuscript, either now or in the future?
- Do you hold any stocks or shares in an organisation that may in any way gain or lose financially from the publication of this manuscript, either now or in the future?

- Do you hold or are you currently applying for any patents relating to the content of the manuscript?
- Have you received reimbursements, fees, funding, or salary from an organization that holds or has applied for patents relating to the content of the manuscript?
- Do you have any other financial competing interests?
- Do you have any non-financial competing interests in relation to this paper?

If you can answer no to all of the above, write 'I declare that I have no competing interests' below. If your reply is yes to any, please give details below.

I declare that I have no competing interests

I agree to the open peer review policy of the journal. I understand that my name will be included on my report to the authors and, if the manuscript is accepted for publication, my named report including any attachments I upload will be posted on the website along with the authors' responses. I agree for my report to be made available under an Open Access Creative Commons CC-BY license (http://creativecommons.org/licenses/by/4.0/). I understand that any comments which I do not wish to be included in my named report can be included as confidential comments to the editors, which will not be published.

Choose an item.

To further support our reviewers, we have joined with Publons, where you can gain additional credit to further highlight your hard work (see: https://publons.com/journal/530/gigascience). On publication of this paper, your review will be automatically added to Publons, you can then choose whether or not to claim your Publons credit. I understand this statement.

Yes Choose an item.
